# Sonographic normal values for the cross-sectional area of the ulnar nerve: a systematic review and meta-analysis

**DOI:** 10.1007/s40477-022-00661-8

**Published:** 2022-02-19

**Authors:** Nadine Boers, Enrico Martin, Marc Mazur, David D. Krijgh, Monique H. M. Vlak, Godard C. W. de Ruiter, H. Stephan Goedee, J. Henk Coert

**Affiliations:** 1 Department of Plastic Surgery, Utrecht Medical Center, Utrecht, The Netherlands; 2 Department of Neurology, Haaglanden Medical Center, The Hague, The Netherlands; 3 Department of Neurosurgery, Haaglanden Medical Center, The Hague, The Netherlands; 4 Department of Neurology, Utrecht Medical Center, Utrecht, The Netherlands; 5 Department of Plastic, Reconstructive, and Hand Surgery, University Medical Center Utrecht, Heidelberglaan 100, 3584 CX Utrecht, The Netherlands

**Keywords:** Ultrasound, Ulnar nerve, Cross-sectional area, Reference values, Normal values

## Abstract

**Purpose:**

Nerve size is a commonly used sonographic parameter when assessing suspected entrapment of the ulnar nerve. We aimed to create a robust set of normal values, based on a critical review of published normal values.

**Methods:**

We performed a systematic evaluation of studies on normal ulnar nerve sizes, identified in PubMed, Embase, and Cochrane databases. Using meta-analyses, we determined pooled mean cross-sectional area (CSA) values for different anatomical locations of the ulnar nerve throughout the arm. Subgroup analyses were performed for gender, probe frequency, in- or exclusion of diabetic patients, position of the elbow and Asian versus other populations.

**Results:**

We identified 90 studies of which 77 studies were included for further analyses after quality review, resulting in data from 5772 arms of 3472 participants. Subgroup analyses show lower CSA values at at the wrist crease and proximal to the wrist crease when using low frequency probes (< 15 MHz) and at the wrist crease, proximal to the wrist crease, proximal forearm and the distal upper arm in Asians. CSA values were lower when in flexed position compared to extended position for the cubital tunnel inlet only. No difference was found for gender.

**Conclusions:**

Our systematic review provides a comprehensive set of normal values at sites along the entire length of the ulnar nerve. This provides a foundation for clinical practise and upon which future studies could be more systematically compared.

**Supplementary Information:**

The online version contains supplementary material available at 10.1007/s40477-022-00661-8.

## Introduction

Ultrasound is increasingly used in the diagnosis of neuropathies, including ulnar nerve entrapment, and importantly complementing electrodiagnostic studies. As point of care device, advantages of nerve ultrasound include low cost, practical non-invasive bedside testing, wide availability and flexible field of view [1]. It can provide essential diagnostic information on relevant morphological changes, such as precise anatomical site and exact nature [2–4]. Several sonographic findings have been proposed to aid in diagnosis of ulnar nerve entrapment: swollen segment surrounding the nerve, increased intraneural vascularity, reduced mobility, blurred margins, and a loss of the fascicular pattern [5]. At present, the nerve size, expressed in cross-sectional area (CSA), appears to be the most robust sonographic parameter. An expert panel recently agreed that ultrasound of the ulnar nerve should include assessment of the CSA and nerve mobility at the elbow and that the entire ulnar nerve from wrist to axilla should be imaged [6].

Unfortunately, previous studies used diverse ultrasound protocols in evaluation of ulnar neuropathy. Consequently, diagnostic cut-off values for CSA measurements vary between studies, resulting in different sensitivities and specificities [3]. Besides, most of the reported variability in literature stem from a lack of standardized normal values for the ulnar nerve. In diagnostic accuracy studies, control groups are often small, comparable to the size of the symptomatic group. The few studies that provide reference values ha for only four locations of the ulnar nerve [7]. Adding data from control groups makes it possible to create reference values at more locations along the ulnar nerve. Therefore, we aimed to systematically evaluate published data on sonographic normal values for ulnar nerve size to create a robust set of reference values which can be used in clinical and research settings.

## Methods

We performed a review in accordance to the PRISMA (Preferred Reporting Items for Systematic Reviews and Meta-Analysis) guidelines, registered in PROSPERO, the International Prospective Register of Systematic Reviews before conduction (registration number: CRD42021232492) [8].

### Search strategy

We performed databases searches on January 21st 2021 in PubMed, Embase, and Cochrane Library using synonyms, key terms, and MeSH descriptors or Emtree terms for the following words: ‘ulnar’, ‘ultrasound’, ‘cross-sectional area’, and ‘reference’. Conference abstracts were excluded. Full search strategies for all three databases are shown in Online Supplementary File A, Table 1. After screening of titles and abstracts, we screened full-text articles for final eligibility. We searched trough reference for additional papers. Screening was performed by two independent researchers (N.B. and M.M.) and discrepancies in selected papers were discussed with a third reviewer (S.G.) until consensus was reached.

### Study selection

We included studies when the following criteria were met: (1) direct CSA measurements of the ulnar nerve were performed in healthy participants; (2) clear description of the study population and sonographic protocol for image acquisition, including the anatomic locations of the ulnar nerve measurements. Exclusion criteria were lack of full text, case reports, conference abstracts, reviews and language other than English.

### Data extraction

**Fig. 1:**
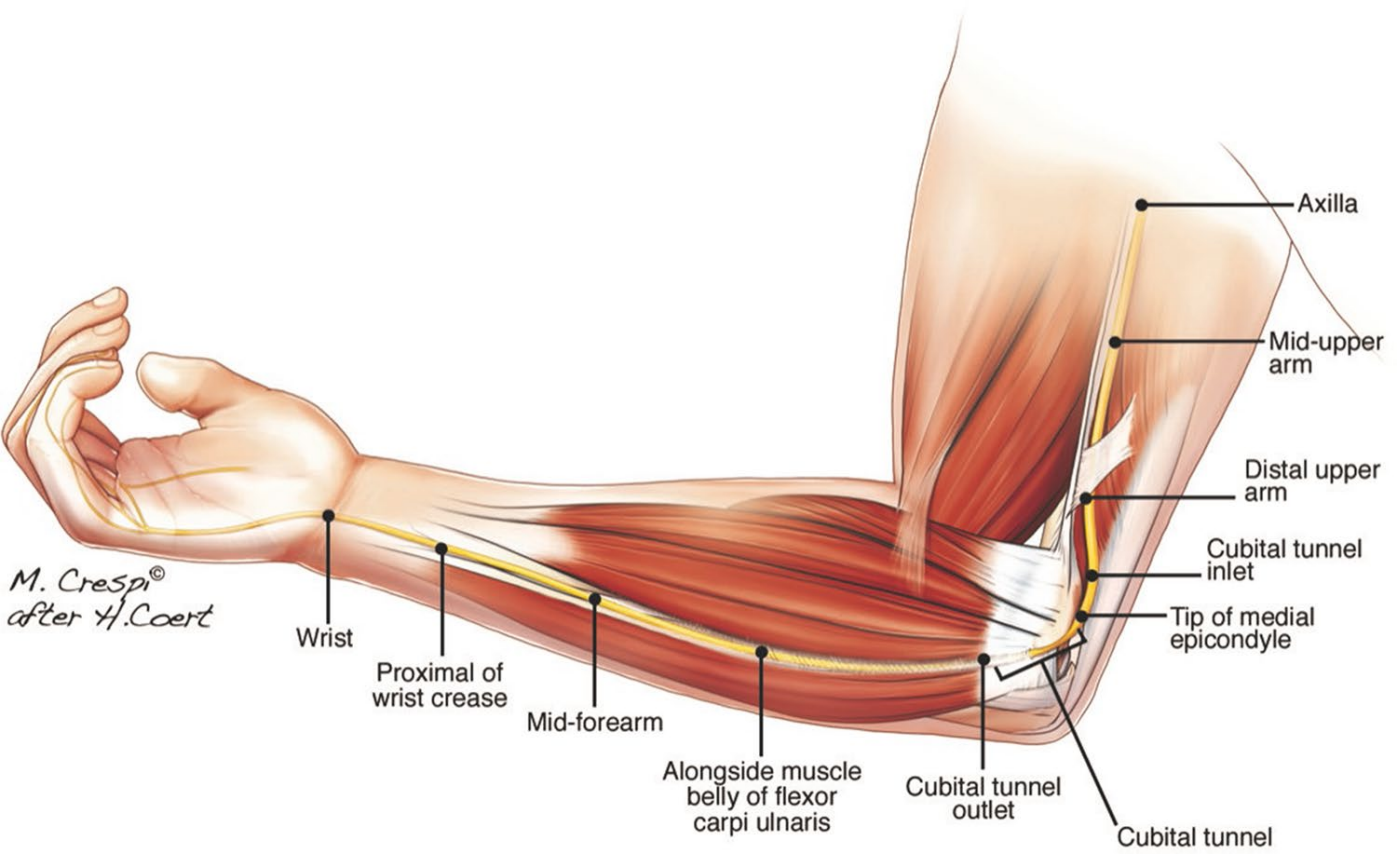
Anatomical landmarks. 11 anatomical locations: the wrist crease (the distal wrist crease and Guyon’s canal), proximal of wrist crease (from 2 cm proximal to distal crease to distal 1/3rd of the forearm), mid-forearm (where the ulnar artery and nerve make contact), proximal forearm (2 cm proximal to the contact point of ulnar artery and nerve to 3 cm distal to tip of the medial epicondyle), cubital tunnel outlet (1–2 cm distal to medial epicondyle, between the two heads of the flexor carpi ulnaris muscle), cubital tunnel (maximal CSA measurements between cubital tunnel inlet and outlet), tip of the medial epicondyle, cubital tunnel inlet (1–2 cm proximal to medial epicondyle), distal upper arm (4–5 cm proximal to tip of the medial epicondyle), mid-upper arm and axilla. The authors hold the copyright for this figure as it was made specifically for this article

**Fig. 2:**
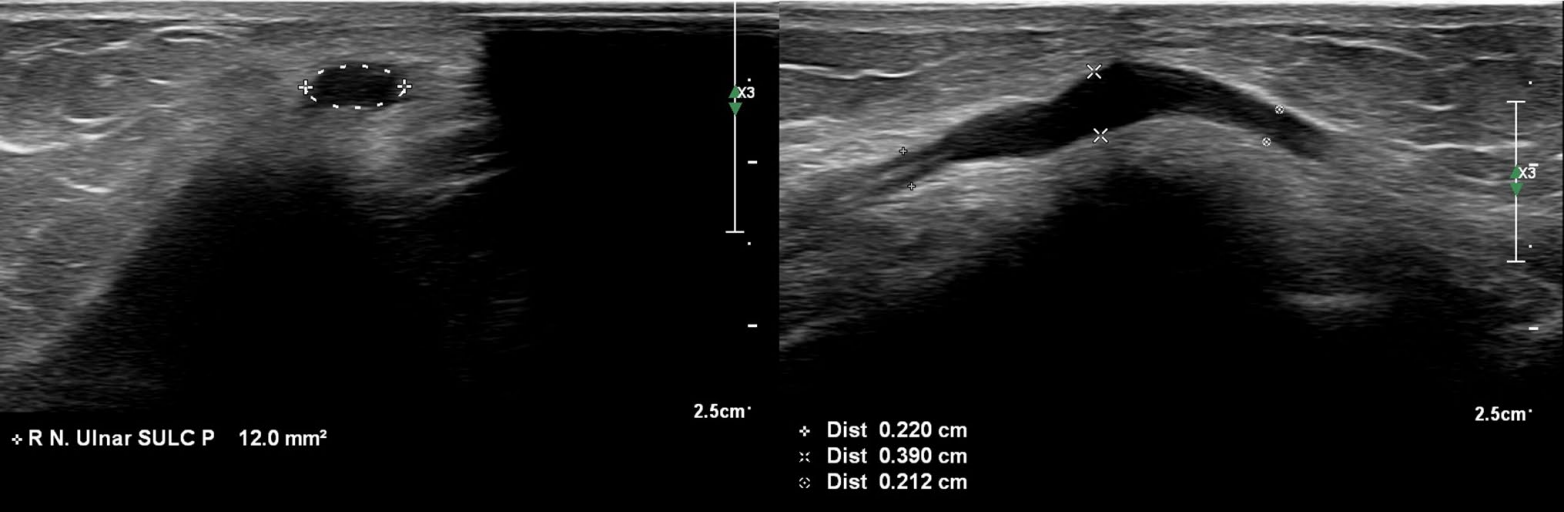
Ultrasound image of the ulnar nerve at the cubital tunnel. Left: a cross-sectional view of the ulnar nerve at the cubital tunnel. Right: a longitudinal view of the ulnar nerve at the cubital tunnel where thickening of the nerve is shown

Two independent researchers (N.B. and M.M.) extracted study, patient, and ultrasound characteristics from included studies and noted mean or median CSA values with 95% confidence interval (CI), standard deviation (SD) and/or range for all available anatomic locations. We categorized the anatomic locations into 11 categories, as shown in Fig. 1. Figure 2 shows an example of the ulnar nerve and its CSA measurement at the cubital tunnel, a possible point of compression.

For each study we also extracted data on size of study population, whether or not patients with diabetes were excluded, demographic data, number and side of arms studied, the type of probe (< 15 mHz or > 15 mHz) and the position of the elbow at investigation (extended, flexed or unknown).

We contacted twenty authors by email when relevant information on patient characteristics, methods or outcomes were missing. After reminder mails, we received additional information from six authors on six studies.

### Quality assessment

Two in-dependent researchers (N.B. and M.M.) appraised the quality of included studies using the Quality Assessment of Diagnostic Accuracy Studies (QUADAS-2) tool, designed to assess the quality of primary diagnostic accuracy studies (Table 2, Online Supplementary File A) [9]. Authors solved disagreement trough discussion. Studies have a high risk of bias when diabetic patients were not specifically excluded, when radiologists were not blinded, when data was collected during different periods and in different settings or when not all patients were included in analyses. Authors had applicability concerns when study conduction or study population was not representative for the research question of the study. When assessing the overall risk of bias, authors did not take the domain ‘reference standard’ into account as we are only interested in data on healthy participants. The Risk of Bias Table and Graphs summarize the quality appraisal for all studies included in the quantitative analyses.

### Statistical analysis

We were able to include studies in quantitative analyses if they reported mean values with SD or 95% CI’s. Using the formulas recommended by to the Cochrane Handbook, we calculated SD values from 95% CI’s [10]. We did not calculate SD values from ranges as this is not recommended.

We performed random effects meta-analyses of mean CSA values with SD when at least three studies reported CSA values. For the main analyses, we included data from both female and male and right and left arm. If papers only displayed subgroup data (i.e. female and male or right and left side), we used pooled mean values. When studies used overlapping data, we selected data from the largest and most appropriate study for inclusion in quantitative synthesis. If studies presented separate CSA values for flexion and extension of the elbow, we used CSA values of flexed elbows in the main analyses as this was considered the standard position and was used in most studies. The *I*^2^ test statistic was used to evaluate heterogeneity of CSA means across studies. *P* < 0.1 or *I*^2^ > 50% was classified as a high degree of heterogeneity among studies.

Subgroup analyses were performed for studies in which (1) men and women were reported separately; (2) probe frequency was noted [< 15 MHz (low resolution) or ≥ 15 MHz (high resolution)]; (3) explicitly reported excluding diabetic patients versus studies who did not report this; (4) flexion of the elbow versus extension of the elbow, and (5) Asian versus other populations. We performed subgroup analyses only when subgroups included at least three studies.

All statistical analyses were performed with R version 4.0.2 (R Core Team, 2021).

## Results

### Search results and study characteristics

**Fig. 3:**
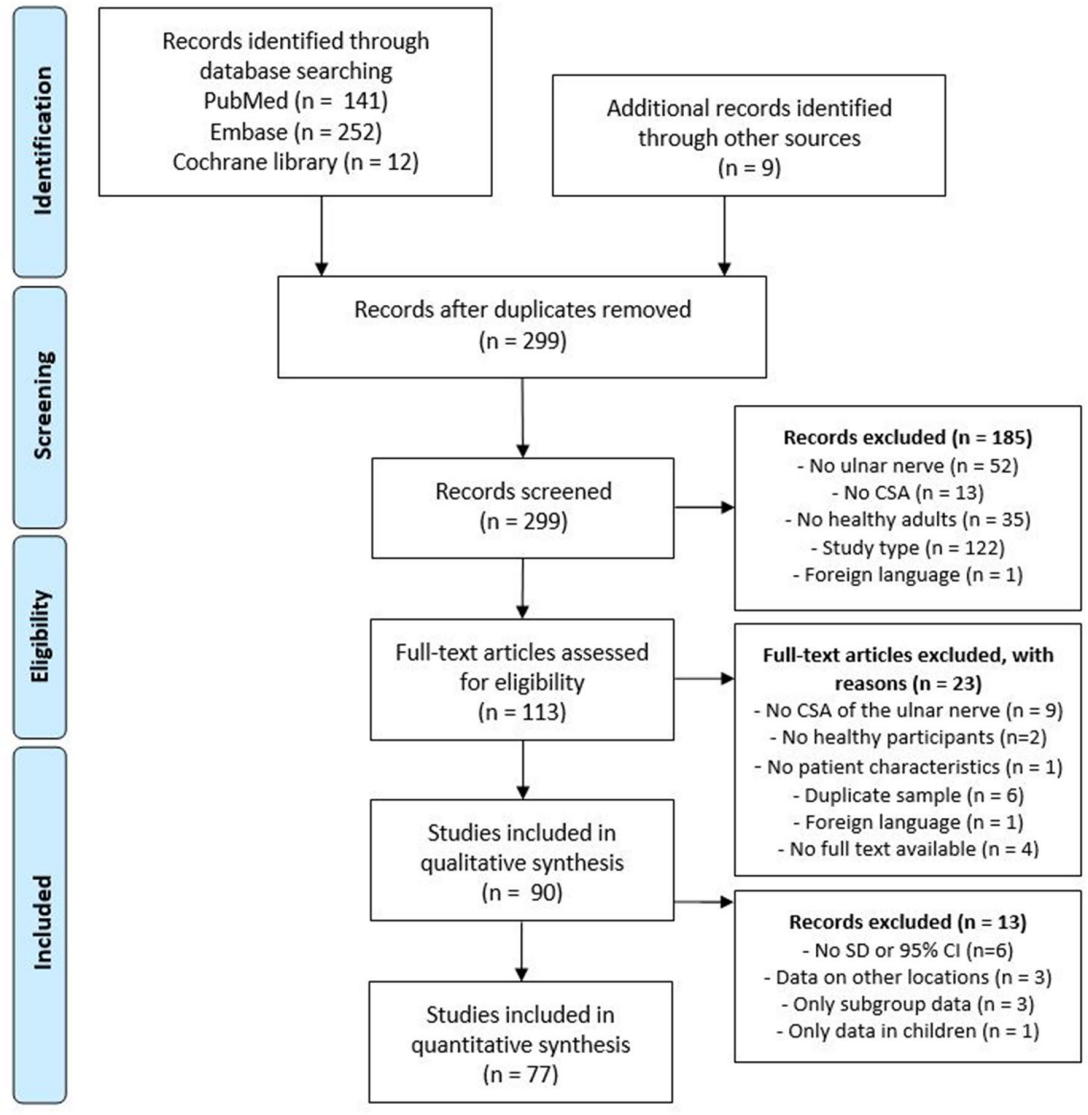
PRISMA flow diagram

Figure 3 summarizes our literature search and study selection. We found 90 studies that met the inclusion criteria, including 6806 elbows of 4206 participants (Online Supplementary File B). Of these, 56 studies compared disease specific CSA values to a control group, and 34 studies included healthy participants with the main goal to determine reference values. Study characteristics for all included studies are shown in Online Supplementary File C, Table 4. The reported CSA values for all included studies are summarized in Online Supplementary File C, Table 5 (adults), Table 6 (children), Table 7 (gender subgroups) and Table 8 (age subgroups).

### Meta-analysis

We included 77 studies for quantitative analyses, including 5772 elbows of 3472 participants. Of these studies, 44 studies had high overall risk of bias, mainly due to not specifically excluding diabetic patients and lack of examiner blinding. The Risk of Bias Graph including outcomes for each domain are shown in Online Supplementary File C, Table 3 and Fig. 1. We found a high degree of heterogeneity between studies in all meta-analyses, including subgroups. Table 1 summarizes the pooled mean CSA values of the ulnar nerve, stratified for anatomical location. Online Supplementary File C, Fig. 2 shows forest plots of pooled mean CSA values.

**Table 1:**
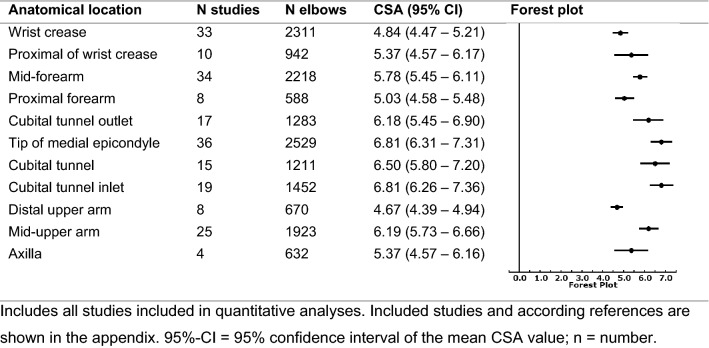
Pooled CSA stratified for anatomical level of the ulnar nerve

#### Subgroup analyses

Pooled CSA values were lower for subgroups with a low frequency probe (range < 15 MHz) compared to high frequency probe (range ≥ 15 MHz) at the wrist crease and proximal to the wrist crease. CSA values were lower for the Asian population compared to the non-Asian population at the wrist crease, proximal to the wrist crease, proximal forearm and the distal upper arm. Only for the cubital tunnel inlet, CSA values were lower with the elbow in flexed position compared to extended position. We found no difference for gender and between studies explicitly excluding diabetes patients and those who did not. Statistically significant different pooled CSA values for the subgroup analyses are shown in Table 2. An overview of all subgroup analyses and corresponding forest plots are shown in Online Supplementary File C, Table 9 and Fig. 3.

**Table 2:** Subgroup analyses with significantly different pooled CSA values

Position	Subgroup		*N* of studies	Mean CSA (95% CI)	*p*-value
Wrist crease	Hz	< 15 MHz	18	4.56 (4.08–5.03)	0.048
		≥ 15 MHz	15	5.17 (4.79–5.55)	
	Asian	Asian population	14	4.25 (3.75–4.74)	0.001
		Other	19	5.26 (4.91–5.61)	
Proximal of wrist crease	Hz	< 15 MHz	6	4.74 (4.03–5.44)	0.004
		≥ 15 MHz	3	6.67 (5.57–7.77)	
	Asian	Asian population	6	4.74 (4.03–5.44)	0.004
		Other	3	6.67 (5.57–7.77)	
Proximal forearm	Asian	Asian population	3	4.42 (4.06–4.77)	0.006
		Other	5	5.42 (4.80–6.04)	
Cubital tunnel inlet	Position	Flexion	9	7.25 (6.70–7.80)	0.003
		Extension	3	6.09 (5.72–6.45)	
		Unknown	7	6.48 (5.45–7.51)	
Distal upper arm	Asian	Asian population	4	4.30 (4.07–4.53)	0.029
		Other	4	4.87 (4.42–5.32)	
		Male	4	5.69 (4.85–6.52)	

Only subgroup analyses with p-values below 0.05 are shown

*95% CI* 95% confidence interval of the mean CSA value, *CSA* cross-sectional area, *n* number. *p* values < 0.05 were considered significant

### Branches of the ulnar nerve in the hand

CSA values of branches of the ulnar nerve (i.e. deep branch of the ulnar nerve, superficial branch of the ulnar nerve, dorsal ulnar cutaneous nerve and palmar ulnar cutaneous nerve) in the hand were described in only four studies and are shown in Table C.2 (Online Supplementary File C) [11–14].

## Discussion

Our meta-analyses resulted in a robust set of normal values specific anatomical sites along the entire length of the ulnar nerve. We found lower CSA values in low resolution probes (< 15 MHz) and with > 90° flexion of the elbow. Also we found lower CSA values in the Asian population, which therefore may warrant modified reference values. No difference was found for gender.

Diagnostic ultrasound of the ulnar nerve often relies on evaluation of nerve size, commonly rated as CSA on transverse images. Several other sonographic parameters have been proposed to be of potential additional value, however, the number of studies on these are still limited. Experts recently agreed that not only the elbow, but the entire length of the ulnar nerve should be imaged [6]. Therefore, our meta-analyses focused on CSA values for unique anatomical sites along the length of the ulnar nerve.

We have identified and analysed different equipment and techniques, including different probe frequencies (< 12 MHz, 12–15 MHZ, 15–18 MHz or a broader range), tracing methods (automatic tracing, free-hand tracing, ellipsoid function or irregular tracing according to the nerve shape), scan protocols, position of the arm (90° flexion in the elbow, > 90° flexion, < 90° flexion, extension or not described), and blinding of the ultrasonographer. Deploying ultrasound probes with lower frequencies warrant caution as image resolution is lower and consequently, the nerve boundaries are less sharp, thus making it more difficult to accurately measure the nerve [15]. Consequently, our subgroup analyses, showed lower pooled CSA values of the ulnar nerve at, and proximal to, the wrist in studies using a lower probe frequency (< 15 MHz versus ≥ 15 MHz). Previous data on zoom magnification, affecting the CSA values, indicated that (lower) resolution should be taken into account when assessing sonographic nerve size [16]. The fact that CSA values decrease with a distal gradient may further compound this. Regarding the position, flexion of the elbow at 90° was considered the standard position and was used in most studies. However, flexion ranged from 40° to 135° and some studies performed measurement with the elbow in extension. As peripheral nerves are capable of stretch and deformation, the position of the upper limb is reported to influence the geometric shape and position of the ulnar nerve [17–21]. Kutlay and Roodt et al. reported a higher CSA in extended upper limbs, contrasting our finding of a higher pooled CSA at the cubital tunnel inlet with the elbow in flexed position [17, 22].

Besides these technical aspects, patient characteristics such as ethnicity may also influence CSA values. We found studies on Asian populations to report a lower CSA at several anatomical sites. A plausible explanation could be racial and ethnic differences among the Asian population compared to non-Asian, or a possibly lower BMI [23]. This is in line with the study of Walhout-van Burg et al. who found that CSA reference values for the median nerve were significantly lower in Asian subjects compared to Dutch people, even after correcting for age, height, and weight [24]. Another explanation could be that studies on Asian populations used lower Hz frequencies more often (81% in Asian studies versus 59% in all studies included in the meta-analyses). However, we were not able to conduct subgroup analyses on use of lower/higher Hz frequencies within studies on Asian populations since too little studies used a higher frequency.

BMI has been suggested to influence the CSA value as well. A study comparing American and European CSA values of the median nerve found significantly higher CSA values in the American cohort, possibly explained by the generally higher BMI in the American population [25]. In our study, only three American studies were included in the meta-analyses in which CSA values were mostly comparable or a little higher than in other studies [26–28]. Only one study, including extremes of BMI (mean BMI of 35.2 kg/m^2^), had noticeably higher CSA values, supporting the hypothesis that BMI may influence the CSA value. One of the included studies compared CSA values of the ulnar nerve between subgroups based on BMI (< 25 kg/m^2^ versus ≥ 25 kg/m^2^) and found no difference at the wrist and in the hand [14]. However, included participants all had a BMI below 30.0 kg/m^2^ and do not represent extremes of BMI.

Our study has several limitations. We were not able to include all studies in the quantitative analyses because of reported median values or missing SD or 95% CI values. However, as these included only few studies and raw data shows that these values are not outliers, we feel it is unlikely that this may have influenced our pooled CSA values. Also, of the identified studies, 13% had unknown and 59% had high risk of bias. This was mostly due to patient selection as they did not specifically (report to) exclude diabetic patients. Since our primary aim is to obtain reference values, ideally, diabetic patients are excluded from analyses. Patient with diabetes may harbor larger CSA values, as has been suggested by previous studies that found higher values in carpal tunnel syndrome without diabetic neuropathy, and the ulnar nerve in (subclinical) diabetic peripheral neuropathy [23, 29–31]. However, we expect that the amount of diabetics included is negligible and therefore, is not likely to have influenced our pooled CSA values.

## Conclusion

We provided a robust set of normal values at several anatomical sites of the ulnar nerve that can be used in diagnostic and research settings. Our study found lower CSA values in Asian populations which therefor may need modified reference values. Our findings also indicate that the diverse techniques used to perform ultrasound warrant further standardisation of the diagnostic ultrasound procedures, including CSA measurements, to avoid unwanted variation in diagnostic performance and improve comparison in future research.

## Supplementary Information

Below is the link to the electronic supplementary material.

Supplementary file1 (PDF 277 kb)

Supplementary file2 (PDF 144 kb)

Supplementary file3 (PDF 7865 kb)
