# Supplementary material for: Sonographic normal values for the cross-sectional area of the ulnar nerve: a systematic review and meta-analysis
Source: J Ultrasound. 2022 Feb 19;26(1):81–8. doi: 10.1007/s40477-022-00661-8 (PMC10063700; doi:10.1007/s40477-022-00661-8)
Supplement: Supplementary file 1 — Supplementary file1 (PDF 277 kb) [file 40477_2022_661_MOESM1_ESM.pdf]

Supplementary file A – Search query and quality assessment

Sonographic normal values for the cross-sectional area of the ulnar nerve: a systematic review and meta-analysis.

Nadine Boers MD[1], Enrico Martin MD PhD[1], Marc Mazur BSc[1], David D. Krijgh MD[1], Monique H.M. Vlak MD PhD[2], Godard C.W. de Ruiter MD PhD[3], H. Stephan Goedee MD PhD[4], J. Henk Coert MD PhD[1]

[1] Department of Plastic Surgery, Utrecht Medical Center, Utrecht, The Netherlands

[2] Department of Neurology, Haaglanden Medical Center, The Hague, The Netherlands

[3] Department of Neurosurgery, Haaglanden Medical Center, The Hague, The Netherlands

[4] Department of Neurology, Utrecht Medical Center, Utrecht, The Netherlands

Corresponding author: N. Boers, MD. e-mail address: n.boers-2@umcutrecht.nl

Index

Table 1 - Search query

Table 2 - QUADAS-2 tool used for quality assessment in this study

Figure 1 - Risk of bias graph for each domain for the studies included in the quantitative analyses

Table 3 - Risk of bias table using the QUADAS-2 tool for separate domains for all selected studies

Table 1 - Search query

Database	Search
Pubmed	Ulnar [tiab] AND (Sonography [tiab] OR ultrasound [tiab] OR ultrasonography [tiab] OR ultrasound [mesh]) AND (Cross-sectional area* [tiab] OR Cross sectional area* [tiab] OR CSA [tiab] OR nerve size* [tiab] OR nerve width [tiab] OR surface area* [tiab]) AND (healthy [tiab] OR reference [tiab] OR volunteers [tiab] OR normal [tiab] OR normative [tiab])
Embase	Ulnar:ti,ab,kw AND (Sonography:ti,ab,kw OR ultrasound:ti,ab,kw OR ultrasonography:ti,ab,kw) AND (‘Cross-sectional area*’:ti,ab,kw OR ‘Cross sectional area*’:ti,ab,kw OR CSA:ti,ab,kw OR ‘nerve size*’:ti,ab,kw OR ‘nerve width’:ti,ab,kw OR ‘surface area*’:ti,ab,kw) AND (healthy:ti,ab,kw OR reference:ti,ab,kw OR volunteers:ti,ab,kw OR normal:ti,ab,kw OR normative:ti,ab,kw)
Cochrane Library	(Ulnar):ti,ab,kw AND ((Sonography):ti,ab,kw OR (ultrasound):ti,ab,kw OR (ultrasonography):ti,ab,kw OR MeSH descriptor: [Ultrasonography] explode all trees) AND ((Cross-sectional area):ti,ab,kw OR (Cross sectional area):ti,ab,kw OR (CSA):ti,ab,kw OR (nerve size*):ti,ab,kw OR (nerve width):ti,ab,kw OR (surface area*):ti,ab,kw) AND ((healthy):ti,ab,kw OR (reference):ti,ab,kw OR (volunteers):ti,ab,kw OR (normal):ti,ab,kw OR (normative):ti,ab,kw)

Table 2 - QUADAS-2 tool used for quality assessment in this study

**DOMAIN 1: PATIENT SELECTION**			
**Risk of bias**	Was a consecutive or random sample of patients enrolled? (Ideally all consecutive, or a random sample of, eligible patients/participants)		Yes / no / ?
	Did the study exclude patients with systemic diseases with a higher change of nerve compression (i.e. diabetes)?		Yes / no / ?
	**Risk of bias**	High / low / uncertain	
	**Explanation**	High risk: ≥1 question(s) are answered with 'no'<br>Low risk: all questions are answered with 'yes'<br>Uncertain risk: ≥1 question(s) are answered with '?' and no question is answered with no	
**Applicability**	Does study population differ from the population targeted with the research question (in term of age, demographic features, co-morbidity etc)?		Yes / no / ?
	**Applicability**	High / low / uncertain	
	**Explanation**	High risk: questions is answered with 'no'<br>Low risk: questions is answered with 'yes'<br>Uncertain risk: questions is answered with '?'	
**DOMAIN 2: INDEX TEST**			
**Risk of bias**	Were the ultrasound results interpreted without knowledge of the results of the reference standard?		Yes / no / ?
	**Risk of bias**	High / low / uncertain	
	**Explanation**	High risk: questions is answered with 'no'<br>Low risk: questions is answered with 'yes'<br>Uncertain risk: questions is answered with '?'	
**Applicability**	Are there concerns that the ultrasound, its conduct, or interpretation differ from the review question (i.e. in technology, execution or interpretation)?		Yes / no / ?
	**Applicability**	High / low / uncertain	
	**Explanation**	High risk: questions is answered with 'no'<br>Low risk: questions is answered with 'yes'<br>Uncertain risk: questions is answered with '?'	
**DOMAIN 3: REFERENCE STANDARD**<br>*This domain is only applicable for studies including patients with a target condition.*			
**Risk of bias**	Is the reference standard likely to correctly classify the target condition?		Yes / no / N/A
	Were the reference standard results interpreted without knowledge of the results of the index test?		Yes / no / N/A
	**Risk of bias**	High / low / uncertain / NA	
	**Explanation**	High risk: ≥1 question(s) are answered with 'no'<br>Low risk: all questions are answered with 'yes'<br>Uncertain risk: ≥1 question(s) are answered with '?' and no question is answered with no<br>N/A: if a study did only include healthy participants	
**Applicability**	Is the target condition in the study the same as the target condition for the ultrasound?		Yes / no / N/A
	**Applicability**	High / low / uncertain / NA	
	**Explanation**	High risk: questions is answered with 'no'<br>Low risk: questions is answered with 'yes'<br>Uncertain risk: questions is answered with '?'<br>N/A: if a study did only include healthy participants	
**DOMAIN 4: FLOW AND TIMING**			
**Risk of bias**	Were ultrasound outcomes collected around the same time (i.e. there was no large difference in time at which the data was collected in case of different hospitals and cohorts)?		Yes / no / N/A
	Were all patients included in the analysis (i.e. patients were not excluded from analyses based on outliers or other (non-explained) reasons)?		Yes / no
	**Risk of bias**	High / low / uncertain	
	**Explanation**	High risk: ≥1 question(s) are answered with 'no'<br>Low risk: all questions are answered with 'yes'<br>Uncertain risk: ≥1 question(s) are answered with '?' and no question is answered with no	

Quality assessment

The Quality Assessment of Diagnostic Accuracy Studies (QUADAS-2 tool) is the current research standard for evaluation of studies validating diagnostic tests and was used to rate study quality of all diagnostic studies included in this review (Table X1). The results of the QUADAS-2 tool indicate risk of bias and applicability concerns for the following categories: patient selection, index test, reference standard and flow and timing (risk of bias only). This study used a modified QUADAS-2 tool using categorical questions adjusted to study design of the included studies (Table X2). The full QUADAS-2 tool can be found on the QUADAS website (www.quadas.org).

Figure 1 - Risk of bias graph for each domain for the studies included in the quantitative analyses

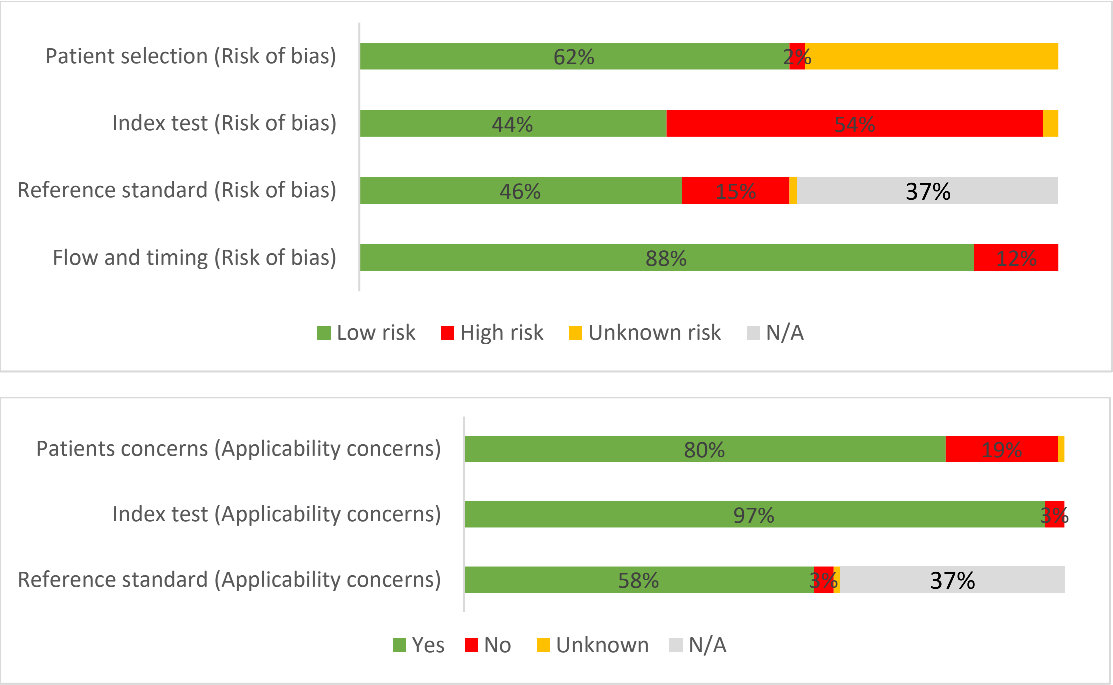

Table 3 - Risk of bias table using the QUADAS-2 tool for separate domains for all selected studies

Article	Year	Risk of bias				Applicability concerns		
		Patient selection	Index test	Reference standard	Flow and timing	Patients concerns	Index test	Reference standard
**Jacob et al.**	2004	?	H	N/A	H	L	L	N/A
**Peeters et al.**	2004	L	H	N/A	L	H	L	N/A
**Wiesler et al.**	2006	?	H	H	L	L	L	L
**Cartwright et al.**	2007	?	H	N/A	L	L	L	N/A
**Ozturk et al.**	2008	?	H	N/A	L	L	L	N/A
**Tagliafico et al. (1)**	2008	L	H	H	L	H	L	L
**Tagliafico et al. (2)**	2008	L	L	L	L	L	L	L
**Thoirs et al.**	2008	L	L	L	H	H	H	L
**Yoon et al. (1)**	2008	?	L	L	L	L	L	L
**Yoon et al. (2)**	2008	?	H	H	L	L	L	L
**Eichenberger et al.**	2009	?	L	L	L	L	L	L
**Elias et al.**	2009	?	L	L	L	L	L	L
**Jain et al.**	2009	L	L	L	L	L	L	L
**Kathirgamanathan et al.**	2009	?	H	N/A	L	L	L	N/A
**Kutlay et al.**	2009	?	L	L	L	L	L	L
**Bayrak et al.**	2010	L	H	H	L	L	L	L
**Hooper et al.**	2011	H	L	L	H	H	L	L
**Ayromlou et al.**	2012	L	L	L	H	L	L	L
**Bathala et al.**	2012	L	H	H	L	L	L	L
**Boom & Visser**	2012	?	H	H	H	L	L	L
**Childs et al.**	2012	L	L	L	H	H	L	L
**Girtler et al.**	2012	L	H	N/A	L	L	L	N/A
**Visser et al.**	2012	L	L	L	L	L	L	L
**Bathala et al.**	2013	?	H	N/A	L	L	L	N/A
**Cartwright et al.**	2013	L	H	N/A	L	H	L	N/A
**Frade et al.**	2013	L	L	L	H	L	L	L
**Kerasnoudis et al.**	2013	?	H	N/A	L	L	L	N/A
**Le Corroller et al.**	2013	?	H	H	L	L	L	L
**Pazzaglia et al.**	2013	L	L	L	L	L	L	L
**Pompe et al.**	2013	L	L	L	L	L	L	L
**Scheidl et al.**	2013	L	H	H	H	L	L	L
**Sugimoto et al.**	2013	?	H	N/A	L	L	L	N/A
**Tagliafico et al.**	2013	L	H	N/A	L	L	L	N/A
**Won et al.**	2013	L	H	N/A	L	L	L	N/A
**Yalcin et al.**	2013	?	H	N/A	L	L	L	N/A
**Boehm et al.**	2014	L	H	N/A	L	L	L	N/A
**Grimm et al. (1)**	2014	L	L	L	L	L	L	L
**Grimm et al. (2)**	2014	?	L	L	L	L	L	L
**Jang et al.**	2014	?	H	L	L	H	L	L
**Scheidl et al.**	2014	L	L	L	L	L	L	L
**Yalcin et al.**	2014	?	L	L	L	L	L	L
**Ellegaard et al.**	2015	L	L	L	L	L	L	L
**Ghanei et al.**	2015	L	L	L	L	L	L	L
**Kim et al.**	2015	L	?	L	L	?	L	L
**Mori et al.**	2015	?	L	L	L	L	L	L
**Reckelhoff et al.**	2015	L	H	H	L	L	L	L
**Roodt et al.**	2015	?	H	N/A	L	L	L	N/A
**Schreiber et al.**	2015	L	H	L	L	H	L	L
**Yiu et al.**	2015	?	H	L	L	L	L	L
**Afsal et al.**	2016	?	H	H	L	H	H	L
**Agirman et al.**	2016	L	L	L	L	L	L	L
**Arumugam et al.**	2016	L	H	L	L	L	L	L
**Cheng et al.**	2016	L	H	L	L	L	L	L
**Dikici et al.**	2016	L	H	N/A	L	L	L	N/A
**Gupta et al.**	2016	L	L	N/A	L	L	L	N/A
**Kang et al.**	2016	L	H	N/A	L	L	L	N/A
**Kose Ozlece et al.**	2016	L	H	L	L	L	L	L
**Merola et al.**	2016	?	L	L	L	L	L	L
**Qrimli et al.**	2016	L	H	N/A	L	L	L	N/A
**Yagci et al.**	2016	L	L	N/A	L	L	L	N/A
**Yurdakul et al.**	2015	L	H	H	L	L	L	L
**Bedewi et al.**	2017	?	H	N/A	L	L	L	N/A
**Chen et al.**	2017	?	L	L	L	L	L	L
**Fink et al.**	2017	?	H	N/A	L	L	L	N/A
**Niu et al.**	2017	?	H	L	H	H	L	H

Article	Year	Risk of bias				Applicability concerns		
		Patient selection	Index test	Reference standard	Flow and timing	Patients concerns	Index test	Reference standard
**Pelosi et al.**	2017	L	L	L	L	H	L	L
**Riegler et al.**	2017	L	H	N/A	L	L	L	N/A
**Atan et al.**	2018	L	L	L	L	L	L	L
**Chen et al.**	2018	?	?	H	L	L	L	L
**Grimm et al.**	2018	L	L	N/A	L	L	L	N/A
**Hobbelink et al.**	2018	L	L	L	L	H	H	H
**Jiwa et al.**	2018	L	L	L	L	L	L	L
**Kim et al.**	2018	?	H	N/A	L	L	L	N/A
**Mulholland et al.**	2018	L	H	N/A	H	L	L	N/A
**Mulroy et al.**	2018	L	L	L	L	L	L	L
**Paluch et al. (1)**	2018	L	L	L	L	H	L	L
**Paluch et al. (2)**	2018	H	L	L	L	H	L	L
**Pelosi et al.**	2018	?	L	L	L	L	L	L
**Schreiber et al.**	2019	L	H	H	L	L	L	H
**Chang et al.**	2019	L	L	L	L	L	L	L
**Druzhinin et al.**	2019	L	L	N/A	L	L	L	N/A
**Lothet et al.**	2019	?	H	N/A	L	H	L	N/A
**Rayegani et al.**	2019	L	L	L	L	L	L	L
**Singh et al.**	2019	L	L	H	H	L	L	L
**Choi et al.**	2020	L	H	N/A	L	H	L	N/A
**Grimm et al.**	2020	L	H	N/A	L	L	L	N/A
**Niu et al.**	2020	L	H	N/A	L	L	L	N/A
**Schubert et al.**	2020	L	H	N/A	L	L	L	N/A
**Tahmaz et al.**	2020	L	H	N/A	L	L	L	N/A
**Tandon et al.**	2020	L	L	?	L	L	L	?
**Bedewi et al.**	2021	?	H	N/A	L	H	L	N/A

L = Low risk H = High risk ? = Unclear risk
