# Supplementary material for: Sonographic normal values for the cross-sectional area of the ulnar nerve: a systematic review and meta-analysis
Source: J Ultrasound. 2022 Feb 19;26(1):81–8. doi: 10.1007/s40477-022-00661-8 (PMC10063700; doi:10.1007/s40477-022-00661-8)
Supplement: Supplementary file 2 — Supplementary file2 (PDF 144 kb) [file 40477_2022_661_MOESM2_ESM.pdf]

Supplementary file B - References of all studies included in qualitative and quantitative analyses

Sonographic normal values for the cross-sectional area of the ulnar nerve: a systematic review and meta-analysis.

Nadine Boers MD[1], Enrico Martin MD PhD[1], Marc Mazur BSc[1], David D. Krijgh MD[1], Monique H.M. Vlak MD PhD[2], Godard C.W. de Ruiter MD PhD[3], H. Stephan Goedee MD PhD[4], J. Henk Coert MD PhD[1]

[1] Department of Plastic Surgery, Utrecht Medical Center, Utrecht, The Netherlands

[2] Department of Neurology, Haaglanden Medical Center, The Hague, The Netherlands

[3] Department of Neurosurgery, Haaglanden Medical Center, The Hague, The Netherlands

[4] Department of Neurology, Utrecht Medical Center, Utrecht, The Netherlands

Corresponding author: N. Boers, MD. e-mail address: n.boers-2@umcutrecht.nl

References

Afsal M, Chowdhury V, Prakash A, Singh S, Chowdhury N. Evaluation of peripheral nerve lesions with high-resolution ultrasonography and color Doppler. Neurology India 2016;64:1002–9. doi: 10.4103/0028-3886.190269

Agirman M, Yagci I, Leblebicier MA, Ozturk D, Akyuz GD. Is ultrasonography useful in the diagnosis of the polyneuropathy in diabetic patients? Journal of Physical Therapy Science 2016;28:2620–4. doi: 10.1589/jpts.28.2620.

Arumugam T, Razali SNO, Vethakkan SR, Rozalli FI, Shahrizaila N. Relationship between ultrasonographic nerve morphology and severity of diabetic sensorimotor polyneuropathy. European Journal of Neurology 2016a;23:354–60. doi: 10.1111/ene.12836.

Atan T, Günendi Z. Diagnostic utility of the sonographic median to ulnar nerve cross-sectional area ratio in carpal tunnel syndrome. Turkish Journal of Medical Sciences 2018;48:110–6. doi: 10.3906/sag-1707-124.

Ayromlou H, Tarzamni MK, Daghighi MH, Pezeshki MZ, Yazdchi M, Sadeghi-Hokmabadi E, Sharifipour E, Ghabili K. Diagnostic value of ultrasonography and magnetic resonance imaging in ulnar neuropathy at the elbow. ISRN Neurol. 2012;2012:491892. doi: 10.5402/2012/491892.

Bathala L, Kumar K, Pathapati R, Jain S, Visser LH. Ulnar neuropathy in hansen disease: clinical, high-resolution ultrasound and electrophysiologic correlations. Journal of Clinical Neurophysiology : Official Publication of the American Electroencephalographic Society 2012;29:190–3. doi: 10.1097/WNP.0b013e31824d969c.

Bathala L, Kumar P, Kumar K, Visser LH. Ultrasonographic cross-sectional area normal values of the ulnar nerve along its course in the arm with electrophysiological correlations in 100 Asian subjects. Muscle & Nerve 2013;47:673–6. doi: 10.1002/mus.23639.

Bedewi MA, Kotb MA, Aldossary NM, Abodonya AM, Alhariqi BA, Swify SM. Shear wave elastography of the ulnar nerve at the forearm. Medicine 2021;100:e24071. doi: 10.1097/MD.0000000000024071.

Bedewi MA, Yousef AMM, Abd-Elghany AA, El-Sharkawy MS, Awad EM. Estimation of ultrasound reference values for the ulnar nerve fascicular number and cross-sectional area in young males. Medicine (United States) 2017;96. doi: 10.1097/MD.0000000000006204.

Boehm J, Scheidl E, Bereczki D, Schelle T, Arányi Z. High-resolution ultrasonography of peripheral nerves: measurements on 14 nerve segments in 56 healthy subjects and reliability assessments. Ultraschall in Der Medizin (Stuttgart, Germany : 1980) 2014;35:459–67. doi: 10.1055/s-0033-1356385.

Boom J, Visser LH. Quantitative assessment of nerve echogenicity: comparison of methods for evaluating nerve echogenicity in ulnar neuropathy at the elbow. Clinical Neurophysiology : Official Journal of the International Federation of Clinical Neurophysiology 2012;123:1446–53. doi: 10.1016/j.clinph.2011.10.050.

Cartwright MS, Mayans DR, Gillson NA, Griffin LP, Walker FO. Nerve cross-sectional area in extremes of age. Muscle & Nerve 2013;47:890–3. doi: 10.1002/mus.23718.

Cartwright MS, Shin HW, Passmore L V, Walker FO. Ultrasonographic findings of the normal ulnar nerve in adults. Archives of Physical Medicine and Rehabilitation 2007;88:394–6. doi: 10.1016/j.apmr.2006.12.020.

Chang Y-W, Hsieh T-C, Tzeng I-S, Chiu V, Huang P-J, Horng Y-S. Ratio and difference of the cross-sectional area of median nerve to ulnar nerve in diagnosing carpal tunnel syndrome: a case control study. BMC Medical Imaging 2019;19:52. doi: 10.1186/s12880-019-0351-3.

Chen J, Liu J, Zeng J, Wu S, Ren J. Ultrasonographic Reference Values for Assessing Normal Sciatic Nerve Ultrasonography in the Normal Population. Journal of Medical Ultrasound 2018;26:85–9. doi: 10.4103/JMU.JMU_6_17.

Chen J, Wang C-L, Wu S, He S, Ren J. The feasibility of using high-resolution ultrasonography to assess ulnar nerve in patients with diabetes mellitus. Journal of Ultrasonography 2017;17:160–6. doi: 10.15557/JoU.2017.0024.

Cheng Y, Xu X, Chen W, Wang Y. Doppler sonography for ulnar neuropathy at the elbow. Muscle and Nerve 2016;54:258–63. doi: 10.1002/mus.25022.

Childs JT, Phillips M, Thoirs KA. Impact of ancestry and body size on sonographic ulnar nerve dimensions. Radiography 2012;18:100–4. doi:101016/jradi201108002

Choi H, Yoon JS. Sonoanatomy of the deep branch of the ulnar nerve. Muscle and Nerve 2020;61:504–7. doi: 10.1002/mus.26820.

Le Corroller T, Bauones S, Acid S, Champsaur P. Anatomical study of the dorsal cutaneous branch of the ulnar nerve using ultrasound. European Radiology 2013;23:2246–51. doi: 10.1007/s00330-013-2832-z.

Dikici A, Ulasli AM, Dikici O, Eroglu S, Solak O, Toktas H, et al. Median, ulnar and peroneal nerve cross-sectional area as a function of muscle mass and BMI. Journal of Neurological Sciences 2016;33:585–93.

Druzhinin D, Naumova E, Nikitin S. Nerve ultrasound normal values in children and young adults. Muscle and Nerve 2019;60:757–61. doi: 10.1002/mus.26715.

Eichenberger U, Stočkli S, Marhofer P, Huber G, Willimann P, Kettner SC, et al. Minimal local anesthetic volume for peripheral nerve block: A new ultrasound-guided, nerve dimensionybased method. Regional Anesthesia and Pain Medicine 2009;34:242–6. doi: 10.1097/AAP.0b013e31819a7225.

Elias Jr. J, Nogueira-Barbosa MH, Feltrin LT, Furini RB, Foss NT, Marques Jr. W, et al. Role of ulnar nerve sonography in leprosy neuropathy with electrophysiologic correlation. Journal of Ultrasound in Medicine 2009;28:1201–9. doi: 10.7863/jum.2009.28.9.1201.

Ellegaard HR, Fuglsang-Frederiksen A, Hess A, Johnsen B, Qerama E. High-resolution ultrasound in ulnar neuropathy at the elbow: A prospective study. Muscle & Nerve 2015;52:759–66. doi: 10.1002/mus.24638.

Fink A, Teggeler M, Schmitz M, Janssen J, Pisters M. Reproducibility of Ultrasonographic Measurements of the Ulnar Nerve at the Cubital Tunnel. Ultrasound in Medicine & Biology 2017;43:439–44. doi: 10.1016/j.ultrasmedbio.2016.09.022.

Frade MAC, Nogueira-Barbosa MH, Lugao HB, Furini RB, Marques Junior W, Foss NT. New sonographic measures of peripheral nerves: a tool for the diagnosis of peripheral nerve involvement in

leprosy. Memórias Do Instituto Oswaldo Cruz 2013;108:257–62. doi: 10.1590/S0074-02762013000300001.

Ghanei ME, Karami M, Zarezadeh A, Sarrami AH. Usefulness of combination of grey-scale and color Doppler ultrasound findings in the diagnosis of ulnar nerve entrapment syndrome. Journal of Research in Medical Sciences : The Official Journal of Isfahan University of Medical Sciences 2015;20:342–5.

Girtler M-T, Krasinski A, Dejaco C, Kitzler HH, Cui LG, Sherebrin S, et al. Feasibility of 3D ultrasound to evaluate upper extremity nerves. Ultraschall in der Medizin (Stuttgart, Germany : 1980). 2013 Aug;34(4):382–7. doi: 10.1055/s-0032-1325397.

Grimm AS, Schubert C, Grimm A, Stahl JH, Küpper H, Horber V, Kegele J, Willikens S, Wittlinger J, Serna-Higuita L, Winter N, Groeschel S. Normative Observational Nerve Ultrasound Values in School-Age Children and Adolescents and Their Application to Hereditary Neuropathies. Front Neurol. 2020 Apr 28;11:303. doi: 10.3389/fneur.2020.00303. eCollection 2020.

Grimm A, Axer H, Heiling B, Winter N. Nerve ultrasound normal values – Readjustment of the ultrasound pattern sum score UPSS. Clinical Neurophysiology 2018;129:1403–9. doi: 10.1016/j.clinph.2018.03.036.

Grimm A, Décard BF, Axer H. Ultrasonography of the peripheral nervous system in the early stage of Guillain-Barré syndrome. Journal of the Peripheral Nervous System 2014a;19:234–41. doi: 10.1111/jns.12091.

Grimm A, Heiling B, Schumacher U, Witte OW, Axer H. Ultrasound differentiation of axonal and demyelinating neuropathies. Muscle and Nerve 2014b;50:976–83. doi: 10.1002/mus.24238.

Gupta S, Bhatt S, Bhargava SK, Singal A, Bhargava S. High resolution sonographic examination: a newer technique to study ulnar nerve neuropathy in leprosy. Leprosy Review 2016;87:464–75.

Hobbelink SMR, Brockley CR, Kennedy RA, Carroll K, de Valle K, Rao P, et al. Dejerine-Sottas disease in childhood-Genetic and sonographic heterogeneity. Brain and behavior. 2018 Apr;8(4):e00919. doi: 10.1002/brb3.919.

Hooper DR, Lawson W, Smith L, Baker SK. Sonographic features in hereditary neuropathy with liability to pressure palsies. Muscle & Nerve 2011;44:862–7. doi: 10.1002/mus.22199.

Jacob D, Creteur V, Courthaliac C, Bargoin R, Sassus B, Bacq C, et al. Sonoanatomy of the ulnar nerve in the cubital tunnel: A multicentre study by the GEL. European Radiology 2004;14:1770–3. doi: 10.1007/s00330-004-2401-6.

Jain S, Visser LH, Praveen TLN, Rao PN, Surekha T, Ellanti R, et al. High-resolution sonography: A new technique to detect nerve damage in leprosy. PLoS Neglected Tropical Diseases 2009;3. doi: 10.1371/journal.pntd.0000498.

Jang JH, Cho CS, Yang K-S, Seok HY, Kim B-J. Pattern analysis of nerve enlargement using ultrasonography in chronic inflammatory demyelinating polyneuropathy. Clinical Neurophysiology : Official Journal of the International Federation of Clinical Neurophysiology 2014;125:1893–9. doi: 10.1016/j.clinph.2013.12.115.

Jiwa N, Abraham A, Bril V, Katzberg HD, Lovblom LE, Barnett C, et al. The median to ulnar cross-sectional surface area ratio in carpal tunnel syndrome. Clinical Neurophysiology : Official Journal of the International Federation of Clinical Neurophysiology 2018;129:2239–44. doi: 10.1016/j.clinph.2018.08.008.

Kang S, Kim SH, Yang SN, Yoon JS. Sonographic features of peripheral nerves at multiple sites in patients with diabetic polyneuropathy. Journal of Diabetes and Its Complications 2016;30:518–23. doi: 10.1016/j.jdiacomp.2015.12.008.

Kathirgamanathan A, French J, Foxall GL, Hardman JG, Bedforth NM. Delineation of distal ulnar nerve anatomy using ultrasound in volunteers to identify an optimum approach for neural blockade. European journal of anaesthesiology. 2009 Jan;26(1):43–6. doi: 10.1097/EJA.0b013e328318c5b6.

Kerasnoudis A, Pitarokoili K, Behrendt V, Gold R, Yoon M-S. Cross sectional area reference values for sonography of peripheral nerves and brachial plexus. Clinical Neurophysiology : Official Journal of the International Federation of Clinical Neurophysiology 2013;124:1881–8. doi: 10.1016/j.clinph.2013.03.007.

Kim JH, Won SJ, Rhee WI, Park HJ, Hong HM. Diagnostic cutoff value for ultrasonography in the ulnar neuropathy at the elbow. Annals of Rehabilitation Medicine 2015;39:170–5. doi: 10.5535/arm.2015.39.2.170.

Kim KH, Lee SJ, Park BK, Kim DH. Sonoanatomy of sensory branches of the ulnar nerve below the elbow in healthy subjects. Muscle and Nerve 2018;57:569–73. doi: 10.1002/mus.25959. Epub 2017 Sep 23.

Kutlay M, Colak A, Simşek H, Oztürk E, Senol MG, Topuz K, et al. Use of ultrasonography in ulnar nerve entrapment surgery--a prospective study. Neurosurgical Review 2009;32:225–32; discussion 232. doi: 10.1007/s10143-008-0162-4.

Lothet EH, Bishop TJ, Walker FO, Cartwright MS. Ultrasound-Derived Nerve Cross-Sectional Area in Extremes of Height and Weight. Journal of Neuroimaging : Official Journal of the American Society of Neuroimaging 2019;29:406–9. doi: 10.1111/jon.12590.

Merola A, Rosso M, Romagnolo A, Peci E, Cocito D. Peripheral Nerve Ultrasonography in Chronic Inflammatory Demyelinating Polyradiculoneuropathy and Multifocal Motor Neuropathy: Correlations with Clinical and Neurophysiological Data. Neurology Research International 2016;2016:9478593. doi: 10.1155/2016/9478593.

Mori A, Nodera H, Takamatsu N, Shimatani Y, Maruyama K, Oda M, et al. Focal nerve enlargement is not the cause for increased distal motor latency in ALS: Sonographic evaluation. Clinical Neurophysiology : Official Journal of the International Federation of Clinical Neurophysiology 2015;126:1632–7. doi: 10.1016/j.clinph.2014.10.152.

Mulholland JM. The effect of wrist angle on ulnar nerve appearance at Guyon's canal in asymptomatic individuals utilising high-resolution sonography. Sonography 2018;5:164–73. doi: 10.1002/sono.12157

Mulroy E, Pelosi L, Leadbetter R, Joshi P, Rodrigues M, Mossman S, et al. Peripheral nerve ultrasound in Friedreich ataxia. Muscle & Nerve 2018;57:852–6. doi: 10.1002/mus.26012.

Niu J, Cui L, Liu M. Multiple Sites Ultrasonography of Peripheral Nerves in Differentiating Charcot-Marie-Tooth Type 1A from Chronic Inflammatory Demyelinating Polyradiculoneuropathy. Frontiers in Neurology 2017;8:181. doi: 10.3389/fneur.2017.00181.

Niu J, Li Y, Zhang L, Ding Q, Cui L, Liu M. Cross-sectional area reference values for sonography of nerves in the upper extremities. Muscle and Nerve 2020;61:338–46. doi: 10.1002/mus.26781.

Ozlece HK, Huseyinoglu N, Gok M, Ilik F. Ultrasonographic and electrophysiological evaluation of the ulnar nerve in patients diagnosed with carpal tunnel syndrome. Journal of Clinical Neurophysiology 2016;33:464–8. doi: 10.1097/WNP.0000000000000283.

Ozturk E, Sonmez G, Çolak A, Sildiroglu HO, Mutlu H, Senol MG, et al. Sonographic appearances of the normal ulnar nerve in the cubital tunnel. Journal of Clinical Ultrasound 2008;36:325–9. doi: 10.1002/jcu.20486.

Paluch Ł, Noszczyk B, Nitek Ż, Walecki J, Osiak K, Pietruski P. Shear-wave elastography: a new potential method to diagnose ulnar neuropathy at the elbow. European radiology. 2018 Dec;28(12):4932–9. doi: 10.1007/s00330-018-5517-9.

Paluch Ł, Noszczyk BH, Walecki J, Osiak K, Kiciński M, Pietruski P. Shear-wave elastography in the diagnosis of ulnar tunnel syndrome. Journal of plastic, reconstructive & aesthetic surgery : JPRAS. 2018 Nov;71(11):1593–9. doi: 10.1016/j.bjps.2018.08.018.

Pazzaglia C, Minciotti I, Coraci D, Briani C, Padua L. Ultrasound assessment of sural nerve in Charcot-Marie-Tooth 1A neuropathy. Clinical Neurophysiology : Official Journal of the International Federation of Clinical Neurophysiology 2013;124:1695–9. doi: 10.1016/j.clinph.2013.02.020.

Peeters EY, Nieboer KH, Osteaux MM. Sonography of the normal ulnar nerve at Guyon's canal and of the common peroneal nerve dorsal to the fibular head. Journal of Clinical Ultrasound 2004;32:375–80. doi: 10.1002/jcu.20054.

Pelosi L, Leadbetter R, Mulroy E, Chancellor AM, Mossman S, Roxburgh R. Peripheral nerve ultrasound in cerebellar ataxia neuropathy vestibular areflexia syndrome (CANVAS). Muscle & Nerve 2017;56:160–2. doi: 10.1002/mus.25476.

Pelosi L, Mulroy E, Leadbetter R, Kilfoyle D, Chancellor AM, Mossman S, et al. Peripheral nerves are pathologically small in cerebellar ataxia neuropathy vestibular areflexia syndrome: a controlled ultrasound study. European Journal of Neurology 2018;25:659–65. doi: 10.1111/ene.13563.

Pompe SM, Beekman R. Which ultrasonographic measure has the upper hand in ulnar neuropathy at the elbow? Clinical Neurophysiology : Official Journal of the International Federation of Clinical Neurophysiology 2013;124:190–6. doi: 10.1016/j.clinph.2012.05.030

Qrimli M, Ebadi H, Breiner A, Siddiqui H, Alabdali M, Abraham A, et al. Reference values for ultrasonograpy of peripheral nerves. Muscle & Nerve 2016;53:538–44. doi: 10.1002/mus.24888.

Rayegani SM, Raeissadat SA, Kargozar E, Rahimi-Dehgolan S, Loni E. Diagnostic value of ultrasonography versus electrodiagnosis in ulnar neuropathy. Medical Devices (Auckland, NZ) 2019;12:81–8. doi: 10.2147/MDER.S196106.

Reckelhoff KE, Li J, Kaeser MA, Haun DW, Kettner NW. Ultrasound Evaluation of the Normal Ulnar Nerve in Guyon's Tunnel: Cross-sectional Area and Anthropometric Measurements. Journal of Medical Ultrasound 2015;23:171–6. doi: 10.1016/j.jmu.2015.09.002.

Riegler G, Lieba-Samal D, Brugger PC, Pivec C, Platzgummer H, Vierhapper M, et al. High-resolution ultrasound visualization of the deep branch of the ulnar nerve. Muscle & Nerve 2017;56:1101–7. doi: 10.1002/mus.25614.

Riegler G, Lieba-Samal D, Brugger PC, Pivec C, Platzgummer H, Vierhapper M, et al. High-resolution ultrasound visualization of the deep branch of the ulnar nerve. Muscle & nerve. 2017 Dec;56(6):1101–7. doi: 10.1002/mus.25614.

Roodt T, van Dyk B, Jacobs S. Ultrasound diagnosis of ulnar nerve entrapment by confirming baseline cross-sectional area measurement for normal and abnormal nerves. South African Journal of Radiology 2015;19. doi: 10.4102/sajr.v19i1.747

Scheidl E, Böhm J, Farbaky Z, Simó M, Bereczki D, Arányi Z. Ultrasonography of ulnar neuropathy at the elbow: axonal involvement leads to greater nerve swelling than demyelinating nerve lesion. Clinical Neurophysiology : Official Journal of the International Federation of Clinical Neurophysiology 2013;124:619–25. doi: 10.1016/j.clinph.2012.08.027.

Schreiber S, Abdulla S, Debska-Vielhaber G, Machts J, Dannhardt-Stieger V, Feistner H, et al. Peripheral nerve ultrasound in amyotrophic lateral sclerosis phenotypes. Muscle & Nerve 2015;51:669–75. doi: 10.1002/mus.24431.

Schreiber S, Schreiber F, Debska-Vielhaber G, Garz C, Hensiek N, Machts J, et al. Differential involvement of forearm muscles in ALS does not relate to sonographic structural nerve alterations. Clinical Neurophysiology : Official Journal of the International Federation of Clinical Neurophysiology 2018;129:1438–43. doi: 10.1016/j.clinph.2018.04.610.

Schubert C, Grimm AS, Stahl JH, Küpper H, Kegele J, Wittlinger J, et al. Nerve ultrasound reference data in children from two to seven years. Clinical Neurophysiology. 2020 Apr 1;131(4):859–65. doi: 10.1016/j.clinph.2019.12.404.

Singh Y, Dixit R, Singh S, Garg S, Chowdhury N. High resolution ultrasonography of peripheral nerves in diabetic peripheral neuropathy. Neurology India 2019;67:S71–6. doi: 10.4103/0028-3886.250719.

Sugimoto T, Ochi K, Hosomi N, Mukai T, Ueno H, Takahashi T, et al. Ultrasonographic reference sizes of the median and ulnar nerves and the cervical nerve roots in healthy Japanese adults. Ultrasound in Medicine & Biology 2013;39:1560–70. doi: 10.1016/j.ultrasmedbio.2013.03.031.

Tagliafico A, Martinoli C. Reliability of side-to-side sonographic cross-sectional area measurements of upper extremity nerves in healthy volunteers. Journal of Ultrasound in Medicine 2013;32:457–62. doi: 10.7863/jum.2013.32.3.457.

Tagliafico A, Resmini E, Nizzo R, Bianchi F, Minuto F, Ferone D, et al. Ultrasound measurement of median and ulnar nerve cross-sectional area in acromegaly. Journal of Clinical Endocrinology and Metabolism 2008a;93:905–9. doi: 10.1210/jc.2007-1719.

Tagliafico A, Resmini E, Nizzo R, Derchi LE, Minuto F, Giusti M, et al. The pathology of the ulnar nerve in acromegaly. European Journal of Endocrinology 2008b;159:369–73. doi: 10.1530/EJE-08-0327.

Tahmaz M, Yoon MS, Schellinger PD, Philipps J. Cross-sectional area in median and ulnar nerve ultrasound correlates with hand volume. Muscle and Nerve 2020;62:83–8. doi: 10.1002/mus.26881.

Tandon A, Khullar T, Maheshwari S, Bhatt S, Narang S. High resolution ultrasound in subclinical diabetic neuropathy: A potential screening tool. Ultrasound 2020. doi: 10.1177/1742271X20958034.

Thoirs K, Williams MA, Phillips M. Ultrasonographic measurements of the ulnar nerve at the elbow: Role of confounders. Journal of Ultrasound in Medicine 2008;27:737–43. doi: 10.7863/jum.2008.27.5.737.

Visser LH, Jain S, Lokesh B, Suneetha S, Subbanna J. Morphological changes of the epineurium in leprosy: a new finding detected by high-resolution sonography. Muscle & Nerve 2012;46:38–41. doi: 10.1002/mus.23269.

Wiesler ER, Chloros GD, Cartwright MS, Shin HW, Walker FO. Ultrasound in the Diagnosis of Ulnar Neuropathy at the Cubital Tunnel. Journal of Hand Surgery 2006;31:1088–93. doi: 10.1016/j.jhsa.2006.06.007.

Won SJ, Kim B-J, Park KS, Yoon JS, Choi H. Reference values for nerve ultrasonography in the upper extremity. Muscle and Nerve 2013;47:864–71. doi: 10.1002/mus.23691.

Yagci I, Leblebicier MA, Kaplan BM, Gokbakan DO, Akyuz G. Sonographic Measurements Can Be Misleading for Diagnosing Carpal Tunnel Syndrome in Patients with Rheumatoid Arthritis. Acta Reumatolol Port 2016;41:40–4.

Yalcin E, Onder B, Akyuz M. Ulnar nerve measurements in healthy individuals to obtain reference values. Rheumatology International 2013;33:1143–7. doi: 10.1007/s00296-012-2527-9.

Yalcin E, Unlu E, Akyuz M, Karaahmet OZ. Ultrasound diagnosis of ulnar neuropathy: comparison of symptomatic and asymptomatic nerve thickness. The Journal of Hand Surgery, European Volume 2014;39:167–71. doi: 10.1177/1753193413484627.

Yiu EM, Brockley CR, Lee KJ, Carroll K, de Valle K, Kennedy R, et al. Peripheral nerve ultrasound in pediatric Charcot-Marie-Tooth disease type 1A. Neurology. 2015 Feb;84(6):569–74. doi: 10.1212/WNL.0000000000001236.

Yoon JS, Hong S-J, Kim B-J, Kim SJ, Kim JM, Walker FO, et al. Ulnar Nerve and Cubital Tunnel Ultrasound in Ulnar Neuropathy at the Elbow. Archives of Physical Medicine and Rehabilitation 2008;89:887–9. doi: 10.1016/j.apmr.2007.10.024.

Yoon JS, Walker FO, Cartwright MS. Ultrasonographic swelling ratio in the diagnosis of ulnar neuropathy at the elbow. Muscle & Nerve 2008;38:1231–5. doi: 10.1002/mus.21094.

Yurdakul OV, Mesci N, Çetinkaya Y, Külcü DG. Diagnostic significance of ultrasonographic measurements and median-ulnar ratio in carpal tunnel syndrome: Correlation with nerve conduction studies. Journal of Clinical Neurology (Korea) 2016;12:289–94. doi: 10.3988/jcn.2016.12.3.289.
