# Supplementary material for: Sonographic normal values for the cross-sectional area of the ulnar nerve: a systematic review and meta-analysis
Source: J Ultrasound. 2022 Feb 19;26(1):81–8. doi: 10.1007/s40477-022-00661-8 (PMC10063700; doi:10.1007/s40477-022-00661-8)
Supplement: Supplementary file 3 — Supplementary file3 (PDF 7865 kb) [file 40477_2022_661_MOESM3_ESM.pdf]

Supplementary file C - Results

Sonographic normal values for the cross-sectional area of the ulnar nerve: a systematic review and meta-analysis.

Nadine Boers MD[1], Enrico Martin MD PhD[1], Marc Mazur BSc[1], David D. Krijgh MD[1], Monique H.M. Vlak MD PhD[2], Godard C.W. de Ruiter MD PhD[3], H. Stephan Goedee MD PhD[4], J. Henk Coert MD PhD[1]

[1] Department of Plastic Surgery, Utrecht Medical Center, Utrecht, The Netherlands

[2] Department of Neurology, Haaglanden Medical Center, The Hague, The Netherlands

[3] Department of Neurosurgery, Haaglanden Medical Center, The Hague, The Netherlands

[4] Department of Neurology, Utrecht Medical Center, Utrecht, The Netherlands

Corresponding author: N. Boers, MD. e-mail address: n.boers-2@umcutrecht.nl

Index

Table 4 Study characteristics of included papers

Table 5 Mean CSA measurements reported in included studies in adults

Table 6 Mean CSA measurements reported in included studies in children

Table 7 Mean CSA measurements at different anatomical locations of the ulnar nerve, separate for men and women

Table 8 Mean CSA measurements at different anatomical locations of the ulnar nerve for different age groups

Table 9 Subgroup analyses

Fig. 2 Forest plots of the mean CSA at different anatomical levels

Fig. 3 Forest plots of subgroup analyses, stratified for anatomical level

Table 4 Study characteristics of included papers

Article	Year	Country (ethnicity, if mentioned)	Diabetic patients excluded	MHz of probe	Experience of examiner	N of investigators	Position of patient during ultrasound examination	N of measurements taken to calculate mean CSA	Anatomic landmarks of the nerve used for tracing CSA	Tracing method used for CSA measurement	N of subjects	N of elbows	Side of mean CSA	Age in yrs<br><br>Mean with (SD or 95%CI) or [range]	Weight in kg<br><br>Mean with (SD or 95%CI) or [range]	Height in cm<br><br>Mean with (SD or 95%CI) or [range]	BMI in kg/m²<br><br>Mean with (SD or 95%CI) or [range]
**Jacob et al.**	2004	Germany	No	8-13 MHz 9-13MHz	ND	8	Elbow flexed at 90° and the palm of the hand placed flat on a hard surface	≥2	ND	One of two methods: ellipse or contouring. When divided, the CSA was calculated as the sum of CSA's	200	400	B	45.7 (15.6)	.	.	.
**Peeters et al.**	2004	Belgium	Yes	5-12 MHz	ND	ND	Dorsal side of the forearm resting on the table.	ND	ND	Continuous tracing	15	30	B	25.1 [21-32]	66.9 [48–95]	172 [163–184]	22.4 [17.6–28.1]
**Wiesler et al.**	2006	United States	No	5-12 MHz	Experienced , not further specified	ND	Supine position with the arm abducted.	3	Maximum CSA) found in the scan (+/- 2cm medial epicondyle)	ND	30	60	B	30 [24-50]	.	.	.
**Cartwright et al.**	2007	United States	No	15 MHz	Training in neuromuscular ultrasound	Several, reliability testing not performed	Arm in 90° abduction and slightly bent (15°)	ND	Inside the hyperechoic rim of the nerve	Circumferentially tracing	30	60	B	30 [24-50]	71	166	.

Article	Year	Country (ethnicity, if mentioned)	Diabetic patients excluded	MHz of probe	Experience of examiner	N of investigators	Position of patient during ultrasound examination	N of measurements taken to calculate mean CSA	Anatomic landmarks of the nerve used for tracing CSA	Tracing method used for CSA measurement	N of subjects	N of elbows	Side of mean CSA	Age in yrs<br>Mean with (SD or 95%CI) or [range]	Weight in kg<br>Mean with (SD or 95%CI) or [range]	Height in cm<br>Mean with (SD or 95%CI) or [range]	BMI in kg/m^2<br>Mean with (SD or 95%CI) or [range]
**Ozturk et al.**	2008	Turkey	No	5-13 MHz	ND	1	Supine with the arm in 30-40° abduction and the elbow in extension and flexion (135°)	3	ND	Automatically tracing	106	212	B	36.8 [20-78]	.	.	.
**Tagliafico et al. (1)**	2008	Italy	Yes	5-17 MHz	ND	NR, intra- and interobserver variability were good	NR	NR	Detection of the fascicular echotexture	NR	50	NR	ND	54.9 (3.1)	.	.	.
**Tagliafico et al. (2)**	2008	Italy	Yes	5-17 MHz	Experienced radiologist and specifically trained non-radiologist, not specified.	Interobserver agreement was good	NR	NR	Fig 1. displays calipers inside the hyperechoic rim of the nerve, not explicitly described	NR	34	34	ND	41.9 (4.62) [23-79]	.	.	25.1 (0.75)

**Article**	**Year**	**Country (ethnicity, if mentioned)**	**Diabetic patients excluded**	**MHz of probe**	**Experience of examiner**	**N of investigators**	**Position of patient during ultrasound examination**	**N of measurements taken to calculate mean CSA**	**Anatomic landmarks of the nerve used for tracing CSA**	**Tracing method used for CSA measurement**	**N of subjects**	**N of elbows**	**Side of mean CSA**	**Age in yrs**<br><br>**Mean with (SD or 95%CI) or [range]**	**Weight in kg**<br><br>**Mean with (SD or 95%CI) or [range]**	**Height in cm**<br><br>**Mean with (SD or 95%CI) or [range]**	**BMI in kg/m^2**<br><br>**Mean with (SD or 95%CI) or [range]**
**Thoirs et al.**	2008	Australia	Yes	5-13 MHz	Experienced and accredited, not further specified	1	Supine sitting position and the upper limb 90° abducted. Two images with the elbow fully extended, and 1 image with the elbow in a fully flexed.	3	Inside the hyperechoic rim of the nerve	NR	NR	108	Both were measured, either R or L or B were included based on CSA findings	35.92 (11.15)	74.84 (20.51)	169 (8)	26.08 (7.22)
**Yoon et al. (1)**	2008	Korea	No	12 MHz	Muscoskeletal radiologist, experience NR	1	Supine position with the elbow extended and flexed 135°	NR	Along the hyperechoic rim of the nerve	Continuous trace function	20	40	B	40.7	.	.	.
**Yoon et al. (2)**	2008	Korea	No	15 Mhz	NR	NR	Supine position with the elbow flexed to 90°	3	Inside the hyperechoic rim of the nerve	Continuous trace function	30	30	R	30.9	70.4	167.2	-
**Eichenberger et al.**	2009	Switzerland	No	15 MHz	Anesthesiologist, experience NR	2	NR	1	'Outer limit traced on frozen image'; image shows inside hyperechoic rim	NR	17	17	ND	33.5 (7.3)	.	.	22.2 (3.3)

Article	Year	Country (ethnicity, if mentioned)	Diabetic patients excluded	MHz of probe	Experience of examiner	N of investigators	Position of patient during ultrasound examination	N of measurements taken to calculate mean CSA	Anatomic landmarks of the nerve used for tracing CSA	Tracing method used for CSA measurement	N of subjects	N of elbows	Side of mean CSA	Age in yrs<br><br>Mean with (SD or 95%CI) or [range]	Weight in kg<br><br>Mean with (SD or 95%CI) or [range]	Height in cm<br><br>Mean with (SD or 95%CI) or [range]	BMI in kg/m^2<br><br>Mean with (SD or 95%CI) or [range]
**Elias et al.**	2009	Brazil	No	10 MHz	Specialized radiologist with 5 years experience in musculoskeletal ultrasound	1	Seated with elbow at 45°	NR	Inside the hyperechoic rim of the nerve	Freehand delimitation by electronic cursor	20	20	ND	46.5 (16.2)	.	.	.
**Jain et al.**	2009	India	Yes	10-14 MHz	NR	1	NR	1	Inside the hyperechoic rim of the nerve	NR	30	60	B	33 (10)	.	.	.
**Kathirgamanathan et al.**	2009	United Kingdom	No	6–13 MHz	>500 examinations	3, reliability testing not performed	Arm in 90° abduction and 90° flexion	NR	NR	NR	50	100	B	33.3 (7.2)	.	.	24.2 (3.9)
**Kutlay et al.**	2009	Turkey	No	5-13 MHz	NR	1	Patient in semilateral position with the shoulders in 60° flexion. Elbows measured in both flexion and extension.	CSA reported two times, assessed by two investiagtors	A hypoechoic, oval, or round ulnar nerve was surrounded by a narrow hyperechoic rim	NR	40	80	B	42 [21 - 62]	.	.	.
**Bayrak et al.**	2010	Turkey	Yes	12 MHz	Radiologist	1	Prone with arms placed near the body in slight flexion (15°)		Inside the hyperechoic rim of the nerve	Manual tracing	21	42	B	40	172.4	68.3	

Article	Year	Country (ethnicity, if mentioned)	Diabetic patients excluded	MHz of probe	Experience of examiner	N of investigators	Position of patient during ultrasound examination	N of measurements taken to calculate mean CSA	Anatomic landmarks of the nerve used for tracing CSA	Tracing method used for CSA measurement	N of subjects	N of elbows	Side of mean CSA	Age in yrs Mean with (SD or 95%CI) or [range]	Weight in kg Mean with (SD or 95%CI) or [range]	Height in cm Mean with (SD or 95%CI) or [range]	BMI in kg/m^2 Mean with (SD or 95%CI) or [range]
**Hooper et al.**	2011	Canada	No	5-12 MHz	NR	1	Elbow flexed at 90° while placing the palm on a tray table	3	Inside the hyperechoic rim of the nerve	Direct trace method	32	64	B	33.3 (11.7)	70.1 (13.0)	172 (9)	23.7 (3.5)
**Ayromlou et al.**	2012	Iran	Yes	7-14 MHz	NR	1	Seated with the elbow flexed to 90°	1	Inside the hyperechoic rim of the nerve	Automatic manual tracing	23	35	ND	42.1 (12.9)	.	.	.
**Bathala et al.**	2012	Netherlands	Yes	6-8 MHz	NR	1	Supine with the elbow flexed at 70°	NR	Inside the hyperechoic rim of the nerve	NR	22	44	B	34.09 (15) [18-70]	.	.	.
**Boom & Visser**	2012	Netherlands	No	7-18 MHz	Experienced neurologist	NR	NR	1	Hyperechoic rim	NR	37	37	B	45 (14)	.	.	.
**Childs et al.**	2012	Australia (European and vietnamese)	Yes	5-13 MHz	Accredited and experienced sonographer	1	Supine with the arm 90° abducted from the body, the elbow extended and the palm facing upwards.	NR	Inside the hyperechoic rim of the nerve	Estimated by calculating the product of the short and long axis measurements	European =24 Vietnamese = 12 Total = 36	71	B	[20-30]	European: 71.81 (19.72) [69.18-74.42] Vietnamese: 59.79 (10.36) [57.7-61.69]	European: 172.42 (11.36) [170.8-173.98] Vietnamese: 162.14 (9.63)[160.24-163.69]	European: 24.23 (6.69) [23.29-25.11] Vietnamese: 22.91 (3.33) [22.3-23.5]
**Girtler et al.**	2012	Germany	Yes	7-18 MHz, set at 18 (3D)	3 years and trained for 4 weeks	2	Lying with the arm extended in supine besides the body in a relaxed position	3 (median instead of mean was calculated)	Outer hypoechoic border of the nerves	Manually drawn line	20	40	B	35 [23-56]	.	.	.

Article	Year	Country (ethnicity, if mentioned)	Diabetic patients excluded	MHz of probe	Experience of examiner	N of investigators	Position of patient during ultrasound examination	N of measurements taken to calculate mean CSA	Anatomic landmarks of the nerve used for tracing CSA	Tracing method used for CSA measurement	N of subjects	N of elbows	Side of mean CSA	Age in yrs<br><br>Mean with (SD or 95%CI) or [range]	Weight in kg<br><br>Mean with (SD or 95%CI) or [range]	Height in cm<br><br>Mean with (SD or 95%CI) or [range]	BMI in kg/m^2<br><br>Mean with (SD or 95%CI) or [range]
**Visser et al.**	2012	Netherlands (Indian)	Yes	10-14 MHz and 12 MHz	NR	1	NR	1	Inside the hyperechoic rim of the nerve	NR	25	25	L or R	31 (8.7)	59.39 (11.48)	162.16 (8.18)	22.82 (3.85)
**Bathala et al.**	2013	India	No	3–12 MHz	NR	1	Supine with the elbow flexed to 70 °	NR	Inside the hyperechoic rim of the nerve	NR	100	200	B	39 (14)	59.25 (11.25)	160 (10)	22.95 (3.50)
**Cartwright et al. (pediatric and geriatrics)**	2013	United States	No	18 MHz	NR	2, intrarater and interrater reliabiilty not assessed	Either seated or lying as they preferred. Arm position is ND	Each image was agreed on by two investigators	NR	Freehand tracing	7<br>8<br>9<br>8<br>20	14<br>16<br>18<br>16<br>40	B	1.2 (0.7)<br>4.8 (0.9)<br>8.8 (1.6)<br>13.8 (1.4)<br>82.2 (7.2)	.	.	17.4 (2.2)<br>16.1 (2.6)<br>16.9 (1.7)<br>22.8 (4.1)<br>24.9 (3.2)
**Frade et al.**	2013	Brasil	Yes	12 MHz	Specialized Radiologist	1	Seated with elbow flexed to 45 °	1	Inner borders of the echogenic rim of the nerve	Freehand delimitation	49	92	B	33.1 (12 - 67)	.	.	.
**Kerasnoudis et al.[1]**	2013	Germany	No	18 MHz	>1 year	3, adequate intrarater and good interrater reliabiilty	NR	3	Inside the hyperechoic rim of the nerve	Continuous tracing	75	150	B	53.46 (14.8)	77.9 (10.68)	175 (9)	.

Article	Year	Country (ethnicity, if mentioned)	Diabetic patients excluded	MHz of probe	Experience of examiner	N of investigators	Position of patient during ultrasound examination	N of measurements taken to calculate mean CSA	Anatomic landmarks of the nerve used for tracing CSA	Tracing method used for CSA measurement	N of subjects	N of elbows	Side of mean CSA	Age in yrs<br><br>Mean with (SD or 95%CI) or [range]	Weight in kg<br><br>Mean with (SD or 95%CI) or [range]	Height in cm<br><br>Mean with (SD or 95%CI) or [range]	BMI in kg/m^2<br><br>Mean with (SD or 95%CI) or [range]
**Le Corroller et al.**	2013	France	No	17.5 MHz	Musculoskeletal radiologists, experience NR	2	Full supination	1	NR	NR	20	20	?	42 [25-61]	-	-	23.7 [20-28]
**Pazzaglia et al.**	2013	Italy	No	18 MHz	Neurologists, experience NR	>1	Seated, arm extended	NR	Inside the hyperechoic rim	Tracing	37	?	?	47.3 [20-69]	.	.	24 [16.20 - 31.45]
**Pompe et al.**	2013	Netherlands	No	5 - 16 MHz	Experienced neurophysiological personnel	NR	Supine with the elbow flexed to 90 °	NR	Within the echogenic rim surrounding the nerve	Automatic ellipse tool or direct tracing	73	73	B	47.2 (10.8) [26-73]	.	.	.
**Scheidl et al.**	2013	Germany or Hungary (unclear)	Yes	15 MHz	NR	>1	Seated, arm on table ,elbow slight flexed (10-20°)	3	Inside the hyperechoic rim of the nerve	Manual tracing	50	87	B	40.6 (11.6)	76.3 (13.5)	167 (7)	.
**Sugimoto et al.**	2013	Japanese	No	7-14 MHz	NR	2, good to bad intra- and interrater variability	Supine position with the examined arm supinated and abducted at body level	NR	Inside the hyperechoic rim of the nerve,	NR	60	120	B	35.4 (9.7)	60.6 (12.1)	164 (10)	22.3 (3.6)

Article	Year	Country (ethnicity, if mentioned)	Diabetic patients excluded	MHz of probe	Experience of examiner	N of investigators	Position of patient during ultrasound examination	N of measurements taken to calculate mean CSA	Anatomic landmarks of the nerve used for tracing CSA	Tracing method used for CSA measurement	N of subjects	N of elbows	Side of mean CSA	Age in yrs<br><br>Mean with (SD or 95%CI) or [range]	Weight in kg<br><br>Mean with (SD or 95%CI) or [range]	Height in cm<br><br>Mean with (SD or 95%CI) or [range]	BMI in kg/m^2<br><br>Mean with (SD or 95%CI) or [range]
**Tagliafico et al.**	2013	Italy	Yes	5-17 MHz	6, 2 and 1 year	3, good inter- and intrarater reliability	NR	NR	Inside the hyperechoic rim of the nerve, the smallest CSA was considered	By the trace function or the ellipsoid formula	40	80	B	41.5 (21-59)	.	.	23.2 (21-24.9)
**Won et al.**	2013	Korea	Yes	5-12 MHz	>3 years	1	Supine with the shoulder flexed to 60° and the elbow flexed to 90-100°	NR	Inside the hyperechoic rim of the nerve, the smallest CSA was considered	Automatically tracing	97	194	R and L seperate	20-69	63.1 (12.2)	165.2 (9.0)	22.9 (3.1)
**Yalcin et al.**	2013	Turkey	No	7–12 MHz	NR	NR, good intrarated variability	Supine with the arm extended 180° from the body	NR	Inside the hyperechoic rim of the nerve If >2 fascicles: as the sum of the CSA of each fascicle	Circumferentially tracing	72	144	B	40.1 (12.3)	73.3 (13.9)	167 (9.8)	26.3 (4.4)
**Boehm et al.**	2014	Germany and Hungary	Yes	12-15 MHz	Both perform ultrasound on a daily basis	2, good inter- and intrarater reliability	Supine, not further specified	3	Inside the hyperechoic rim of the nerve	Manually tracing	31<br>25	31<br>25	L	Germany: 51.8 (16.4)<br>Hungary: 48.5 (15.6)	Germany: 75.4 (13.0)<br>Hungary: 79.6 (18.2)	Germany: 171 (9)<br>Hungary: 168 (6)	.

Article	Year	Country (ethnicity, if mentioned)	Diabetic patients excluded	MHz of probe	Experience of examiner	N of investigators	Position of patient during ultrasound examination	N of measurements taken to calculate mean CSA	Anatomic landmarks of the nerve used for tracing CSA	Tracing method used for CSA measurement	N of subjects	N of elbows	Side of mean CSA	Age in yrs Mean with (SD or 95%CI) or [range]	Weight in kg Mean with (SD or 95%CI) or [range]	Height in cm Mean with (SD or 95%CI) or [range]	BMI in kg/m^2 Mean with (SD or 95%CI) or [range]
**Grimm et al. (1)**	2014	Germany	Yes	18 MHz	NR	2, excellent inter- and intrarater reliability	NR	2 (second examiner evaluated them offline)	Inside the hyperechoic rim of the nerve	NR	8	16	B	49.71 (20.2)	76.0 (17.1)	176.0 (6.1)	.
**Grimm et al. (2)**	2014	Germany	No	14 MHz	NR	2, excellent inter- and intrarater reliability	NR	2 (second examiner evaluated them offline)	Inside the hyperechoic rim of the nerve	NR	21	42	B	53.14 (15.15)	64.29 (17.39)	172.89 (6.79)	.
**Jang et al.**	2014	Korea	No	5-12 MHz	>5 years	2, excellent inter- and intrarater reliability	NR	3	Inside the hyperechoic rim of the nerve	NR	18	36	B	45.9 (16.2)	68.1 (10.0)	168.0 (7.5)	24.2 (3.0)
**Scheidl et al.**	2014	Germany	Yes	15 MHz	NR	1	NR	3	Inside the hyperechoic rim of the nerve	NR	34	34	L	50.6 (17.2)	77.6 (17.6)	167 (6.3)	.

Article	Year	Country (ethnicity, if mentioned)	Diabetic patients excluded	MHz of probe	Experience of examiner	N of investigators	Position of patient during ultrasound examination	N of measurements taken to calculate mean CSA	Anatomic landmarks of the nerve used for tracing CSA	Tracing method used for CSA measurement	N of subjects	N of elbows	Side of mean CSA	Age in yrs<br><br>Mean with (SD or 95%CI) or [range]	Weight in kg<br><br>Mean with (SD or 95%CI) or [range]	Height in cm<br><br>Mean with (SD or 95%CI) or [range]	BMI in kg/m^2<br><br>Mean with (SD or 95%CI) or [range]
**Yalcin et al.**	2014	Turkey	No	7-12 MHz	Physical medicine and rehabilitation physician, experience NR	1	Sitting with the elbow extended	NR	Inside the hyperechoic rim of the nerve	Circumferentially tracing	19	38	B	38.5 (13.1)	72.2 (14.0)	72.2 (14.0)	26.0 (4.5)
**Ellegaard et al.**	2015	Denmark	No	NR	NR	1	Lying on one side with the arm beside the head and the elbow flexed at 80–90°	NR	Inside the hyperechoic rim of the nerve. I.e. >1 fascicle, structures were traced together	Direct tracing	43	43	L	44.88 (16.43)	74.53 (14.27)	174 (10)	24.56 (3.29)
**Ghanei et al.**	2015	Iran	No	5-7 MHz	NR	1	Supine position with the arm abducted	3	Inside the hyperechoic rim of the nerve	Automatic manual tracing	44	44	L or R	39.5 [27-49]	.	.	.
**Kim et al.**	2015	Korea	Yes	7-12 MHz	NR	1	Sitting with their elbows flexed at 90° and their shoulders flexed at 60°, the forearm supinated and resting.	3	Inside the hyperechoic rim of the nerve, maximum CSA at the cubital tunnel inlet	NR	30	30	R	45.2 (13.5)	63.6 (10.7)	165.2 (7.2)	23.3 (3.2)
**Mori et al.**	2015	Japan	No	11 MHz	NR	1	Supine position with the arm supinated and abducted at body level	NR	Inside the hyperechoic rim of the nerve	NR	30	30	R	61.8 (18.1)	59.7 (11.2)	161.9 (9.7)	22.6 (3.0)

Article	Year	Country (ethnicity, if mentioned)	Diabetic patients excluded	MHz of probe	Experience of examiner	N of investigators	Position of patient during ultrasound examination	N of measurements taken to calculate mean CSA	Anatomic landmarks of the nerve used for tracing CSA	Tracing method used for CSA measurement	N of subjects	N of elbows	Side of mean CSA	Age in yrs<br><br>Mean with (SD or 95%CI) or [range]	Weight in kg<br><br>Mean with (SD or 95%CI) or [range]	Height in cm<br><br>Mean with (SD or 95%CI) or [range]	BMI in kg/m^2<br><br>Mean with (SD or 95%CI) or [range]
**Reckelhoff et al.**	2015	United states (Turkisch)	Yes	4-15 MHz	7 and 4 years	3, very good intra- and interrater reliability	NR	NR	Inside the hyperechoic rim of the nerve	The ellipsoid function for a round or oval nerve, the tracing function for an irregular-shaped nerve	46	83	B	24.7 (3.1)	.	.	.
**Roodt et al.**	2015	South Africa	No	12 MHz	NR	NR	Flexion: Seated with the palm on the table and the elbow rotated postero-laterally. Extension: seated with the elbow extended and the dorsal hand on the table	3	Inside the hyperechoic rim of the nerve	Manually tracing	25	25	D	36 [23-56]	.	.	.
**Schreiber et al.**	2015	Germany	Yes	12 MHz	NR	1	Seated with the entire arm extended anteriorly, supinated, and supported by a pillow on a table	3	Inside the hyperechoic rim of the nerve	Continuous manual tracing of nerve circumference	18	36	B	63.8 (9.5)	81.0 (12.7)	174.0 (8.7)	.

Article	Year	Country (ethnicity, if mentioned)	Diabetic patients excluded	MHz of probe	Experience of examiner	N of investigators	Position of patient during ultrasound examination	N of measurements taken to calculate mean CSA	Anatomic landmarks of the nerve used for tracing CSA	Tracing method used for CSA measurement	N of subjects	N of elbows	Side of mean CSA	Age in yrs<br>Mean with (SD or 95%CI) or [range]	Weight in kg<br>Mean with (SD or 95%CI) or [range]	Height in cm<br>Mean with (SD or 95%CI) or [range]	BMI in kg/m^2<br>Mean with (SD or 95%CI) or [range]
**Yiu et al. (pediatric)**	2015	Australia	No	7-12 MHz	Child neurologist and an experienced ultrasonographer	2, excellent interrater reliability	Supine position with the arm supinated	3	Inside the hyperechoic rim of the nerve, the smallest CSA	NR	29	29	D	11.3 (4.4)	.	143.6 (25.5)	.
**Afsal et al.**	2016	India	No	5-17 MHz	NR	NR	Supine position	NR	NR, figure shows inside the hyperechoic rim of the nerve	NR, figure shows irregular tracing according to the nerve shape	10<br>16	20<br>32	B	Age matched with cases: Cases: 56.6 Cases: 28.25	.	.	.
**Agirman et al.**	2016	Turkey	Yes	6-18 MHz	Well-trained, not specified	1	Supine position on a table	3	Inside the hyperechoic rim of the nerve	NR	14	28	B	47.6 (13.1)	.	.	.
**Arumugam et al.**	2016	Malaysia (Malaysian, Chinese, Indian)	Yes	12 MHz	NR	1	NR	NR	Inside the hyperechoic rim of the nerve	Electronic tracer	40	40	ND	57.75 (7.11)	66.81 (12.39)	162.7 (8.99)	.
**Cheng et al.**	2016	China	Yes	7-15 MHz	7 years	1	Supine position. The forearm was held above the head, and the elbow was flexed 70°	NR	Inside the hyperechoic rim of the nerve, accurate to 0.1 mm, largest CSA at scanning traject	Continuous tracing, CSA calculated by a preinstalled area measurement software program	50	100	B	42.7 (14.8)	.	.	.

Article	Year	Country (ethnicity, if mentioned)	Diabetic patients excluded	MHz of probe	Experience of examiner	N of investigators	Position of patient during ultrasound examination	N of measurements taken to calculate mean CSA	Anatomic landmarks of the nerve used for tracing CSA	Tracing method used for CSA measurement	N of subjects	N of elbows	Side of mean CSA	Age in yrs<br><br>Mean with (SD or 95%CI) or [range]	Weight in kg<br><br>Mean with (SD or 95%CI) or [range]	Height in cm<br><br>Mean with (SD or 95%CI) or [range]	BMI in kg/m^2<br><br>Mean with (SD or 95%CI) or [range]
**Dikici et al.**	2016	Turkey	Yes	6-18 MHz	4 years	1	Seated with the elbow in 80-90° flexion and forearm in full supination	3	NR	Circumferentially tracing	233	466	B	40.5 (15.6)	73.5 (16.4)	164.5 (9.6)	27.3 (6.3)
**Gupta et al.**	2016	India	Yes	5-12 MHz	NR	NR	Supine position with the arm slightly abducted with 30° flexion at the elbow	NR	Inside the hyperechoic rim of the nerve	Electronic calipers	30	60	B	38 [10-80]	.	.	.
**Kang et al.**	2016	Korea	Yes	7-12 MHz	>10 years	1, intrarater reliability was excellent	NR	3	Inside the hyperechoic rim of the nerve, Doppler was used to control for blood vessels	Direct tracing, not specified	20	?	?	65.00 (9.83)	60.25 (11.77)	162.10 (11.23)	22.86 (3.39)
**Kose Ozlece et al.**	2016	Turkey	Yes	12-17 MHz	NR	1	Seated with the arm extended 180° and the palms upward and resting on a pillow	NR	Inside the hyperechoic rim of the nerve, smallest CSA was considered	Circumferentially tracing	39	78	B	39.59 (9.130)	.	.	28.6 (3.4)

Article	Year	Country (ethnicity, if mentioned)	Diabetic patients excluded	MHz of probe	Experience of examiner	N of investigators	Position of patient during ultrasound examination	N of measurements taken to calculate mean CSA	Anatomic landmarks of the nerve used for tracing CSA	Tracing method used for CSA measurement	N of subjects	N of elbows	Side of mean CSA	Age in yrs<br><br>Mean with (SD or 95%CI) or [range]	Weight in kg<br><br>Mean with (SD or 95%CI) or [range]	Height in cm<br><br>Mean with (SD or 95%CI) or [range]	BMI in kg/m^2<br><br>Mean with (SD or 95%CI) or [range]
**Merola et al.**	2016	Italy	No	6-15 MHz	NR	1	NR	NR	Inside the hyperechoic rim of the nerve	Ellipse method in case of a round shape and alternative tracing method in case of a irregular shape	70	140	B	58.4 (16.10)	.	.	.
**Qrimli et al.**	2016	Canada	Yes	15 MHz	experience, not specified	3, interrater variability was not tested	Laying supine with the elbow flexed at 90°	NR	Inside the hyperechoic rim of the nerve, smallest CSA was considered	Automatically tracing	100	185	R and L separate	44.1 (18.4)	.	.	25.3 (5.3)
**Yagci et al.**	2016	Turkey	Yes	6-18 MHz	Well trained, not specified	1	Supine position on a table	3	Inside the hyperechoic rim of the nerve	NR	30[F]	60[F]	B[F]	41.56 (10.40)[F]	.	.	27.19 (3.88)[F]
**Yurdakul et al.**	2016	Turkey	Yes	7-13 MHz	Experienced, not specified	1	Sitting with the wrist at a neutral position	3	Tracing of the hypoechoic rim	Continuous tracing	37	62	B	45.16 (13.11)	72.38 (13.82)	162 (6)	27.51 (5.60)
**Bedewi et al.**	2017	Saudi Arabia	No	5-13 MHz	Senior, experienced, not specified	1	Supine with the elbow flexed at 90°	NR	Inside the hyperechoic rim of the nerve	Circumferentially tracing	50	100	B	21.62 (1.70)	71.60 (14.32)	169.50 (6.27)	24.80 (4.11)
**Chen et al.**	2017	China	No	15 MHz	NR	1	NR	3	Inside the hyperechoic rim of the nerve	NR	100	200	B	58.5 (7.7)	51.3 (6.8)	168.7 (8.9)	.

Article	Year	Country (ethnicity, if mentioned)	Diabetic patients excluded	MHz of probe	Experience of examiner	N of investigators	Position of patient during ultrasound examination	N of measurements taken to calculate mean CSA	Anatomic landmarks of the nerve used for tracing CSA	Tracing method used for CSA measurement	N of subjects	N of elbows	Side of mean CSA	Age in yrs Mean with (SD or 95%CI) or [range]	Weight in kg Mean with (SD or 95%CI) or [range]	Height in cm Mean with (SD or 95%CI) or [range]	BMI in kg/m^2 Mean with (SD or 95%CI) or [range]
**Fink et al.**	2017	Netherlands	No	6-13 MHz	3 and 7 years	2, good interrater and excellent intrarater reliability	Sitting with the elbow in 70-90° flexion	The mean of two measurements by two investigators	On the hyperechoic rim of the nerve	Elliptical	73	73	D	36 (15)	72.1 (13.8)	174 (10)	23.82 (3.65)
**Niu et al.**	2017	China	No	8-12 MHz	NR	NR	NR	NR	Inside the hyperechoic rim of the nerve	Continuously tracing	14	18	B in 4<br>R in 10	39 (19-52)	.	.	.
**Pelosi et al.**	2017	New Zealand	Yes	6-15 MHz	NR	1	NR	3	Inside the hyperechoic rim of the nerve	Manual tracing	7	7	L or R	62.6 (12.8)	.	.	.
**Riegler et al.**	2017	Austria	Yes	18-22 MHz	> 4 years	1	Hand on a table with the handpalm up and the wrist extended	NR	NR	Using the software, not specified	10	20	B	31.5 (27-54)	.	.	.
**Atan et al.**	2018	Turkey	No	8-12 MHz	NR	NR	Seated with the forearm resting on a table and the palm supinated	3	Inside the hyperechoic rim of the nerve	Using the trace program of the ultrasound	50	50	L or R	50.68 (17.33)	71.84 (12.38)	160 (5.7)	27.97 (5.05)
**Chen et al.**	2018	China	No	6-13 MHz	NR	NR	Supine or prone position	NR	At the outer borders of the echogenic rims of the nerves	Freehand tracing	29	58	L and R seperate	57.6 (17.9)	.	.	.

Article	Year	Country (ethnicity, if mentioned)	Diabetic patients excluded	MHz of probe	Experience of examiner	N of investigators	Position of patient during ultrasound examination	N of measurements taken to calculate mean CSA	Anatomic landmarks of the nerve used for tracing CSA	Tracing method used for CSA measurement	N of subjects	N of elbows	Side of mean CSA	Age in yrs<br><br>Mean with (SD or 95%CI) or [range]	Weight in kg<br><br>Mean with (SD or 95%CI) or [range]	Height in cm<br><br>Mean with (SD or 95%CI) or [range]	BMI in kg/m^2<br><br>Mean with (SD or 95%CI) or [range]
**Grimm et al.[2]**	2018	Germany	Yes	14 MHz	NR	2, intra- and interrater variability was excellent	NR	NR	Inside the hyperechoic rim of the nerve	NR	100	100	R	51.2 (18.2)	74.5 (24.1)	174.5 (11.4)	.
**Hobbelink et al. (pediatric)**	2018	Australia	Yes	8-18 MHz	NR	NR	NR	Repeated three times with the mean of the measures	Inside the hyperechoic rim of the nerve, the smallest CSA was considered	NR	5	5	D	8.2 (4.0)	.	.	.
**Jiwa et al.**	2018	Canada	Yes	5-15 MHz	Experienced, not specified	1	Seated with the arm supinated and the wrist in a neutral position	3	Inside the hyperechoic rim of the nerve	Continuous tracing	37	37	L or R	49.5 (16.4)	74.4 (17.4)	168 (11)	26.1 (4.2)
**Kim et al.**	2018	Korea	No	5-13 MHz	NR	1	Supine position with the forearms supinated	NR	As a single hypoechic fascicle, excluding the epineurium	NR	30	60	B	35 (12)	60.0 (9.8)	168.8 (8.9)	.
**Mulholland et al.**	2018	Australia	Yes	5-18 MHz	Experienced, not specified	1	Extension of the wrist	3	Inside the hyperechoic rim of the nerve	Continuous tracing	51	51	.	40.4(11.5)[M]<br>40.4 (13.2)[F]	.	.	25.6 (3.4)[M]<br>24.7 (5.0)[F]

Article	Year	Country (ethnicity, if mentioned)	Diabetic patients excluded	MHz of probe	Experience of examiner	N of investigators	Position of patient during ultrasound examination	N of measurements taken to calculate mean CSA	Anatomic landmarks of the nerve used for tracing CSA	Tracing method used for CSA measurement	N of subjects	N of elbows	Side of mean CSA	Age in yrs<br><br>Mean with (SD or 95%CI) or [range]	Weight in kg<br><br>Mean with (SD or 95%CI) or [range]	Height in cm<br><br>Mean with (SD or 95%CI) or [range]	BMI in kg/m^2<br><br>Mean with (SD or 95%CI) or [range]
**Mulroy et al.**	2018	New Zealand	Yes	6-15 MHz	Experienced, not specified	1	NR	3	Inside the hyperechoic rim of the nerve	Manual tracing	8	8	.	48.6 (18.0)	.	.	28.0 (5.3)
**Paluch et al. (1)**	2018	Poland	Yes	5-18 MHz	>4 years	1	Seated position with the examined forearm resting on the examiner's knee	3	NR	NR	39	39	R or L	60.05 [41-93]	.	.	.
**Paluch et al. (2)**	2018	Poland	No	5-18 MHz	>4 years	1	Seated with with flexed elbow and forearm resting on the knee in a supine position	3	Inside the hyperechoic rim of the nerve	NR	38	38	.	57.42 [38-84]	.	.	.
**Pelosi et al.**	2018	New Zealand	No	6-15 MHz	NR	1	NR	3	Inside the hyperechoic rim of the nerve	Manually tracing	14	28	B	67.1 (.)	75.4 (.)	169.6 (.)	.
**Schreiber et al.**	2018	Germany	No	12 MHz	NR	1	NR	NR	Automatically, the intranerval fascicular portion	Continuous manual tracing	18	36	L and R seperate	59 (8)	81 (15)	175 (10)	.
**Chang et al.**	2019	China	Yes	12 MHz	>5 years	1	Laying in the supine position with the elbow extended, forearm supinated, and neutral wrist	3	Inside the hyperechoic rim of the nerve	Continuous tracing	32	59	B	50.1 [30.0–70.2]	.	.	22.1 [16.6–27.5]

Article	Year	Country (ethnicity, if mentioned)	Diabetic patients excluded	MHz of probe	Experience of examiner	N of investigators	Position of patient during ultrasound examination	N of measurements taken to calculate mean CSA	Anatomic landmarks of the nerve used for tracing CSA	Tracing method used for CSA measurement	N of subjects	N of elbows	Side of mean CSA	Age in yrs<br><br>Mean with (SD or 95%CI) or [range]	Weight in kg<br><br>Mean with (SD or 95%CI) or [range]	Height in cm<br><br>Mean with (SD or 95%CI) or [range]	BMI in kg/m^2<br><br>Mean with (SD or 95%CI) or [range]
**Druzhinin et al. (pediatric and adult)**	2019	Russia	Yes	5-18 MHz	Experienced, not specified	2, good intra- and interrated variability	NR	2 by 2 investigators (4 in total)	NR	Manually tracing	11<br>15<br>10<br>7<br>7<br>22	22<br>30<br>20<br>14<br>14<br>44	L and R seperate	3.27 (0.79)<br>5.61 (0.80)<br>8.47 (1.59)<br>11.3 (1.22)<br>14.8 (1.57)<br>24.5 (3.66)	.	.	.
**Lothet et al.**	2019	United States	No	18 MHz	NR	NR	Seated or lying, their extremity placed in a neutral position	NR	Inside the hyperechoic rim of the nerve	Freehand tracing	15	30	B	21.7 (4.0)	102.7 (31.2)	190.5 (11.4)	28.0 (7.5)
**Rayegani et al.**	2019	Iran	Yes	5-12 MHz	NR	1	Supine position with the elbow 90° flexed, wrist in neutral position, shoulder 90° abducted and slightly rotated.	3	Inside the hyperechoic rim of the nerve	By placing electronic calipers around the margin of the nerve	34	34	.	44.5 (34.13–54.93)	.	.	26.5 (23.1–29.9)
**Singh et al.**	2019	India	Yes	5-18 MHz	NR	NR	Supine position	NR	NR	NR	45	45	R	30-68	.	.	.
**Choi et al.**	2020	Korea	Yes	3-12 MHz	NR	1	NR	NR	Inside the hyperechoic rim of the nerve	NR	30	60	B	39 (14)	67 (10.9)	169.8 (8.9)	23.2 (3.2)
**Grimm et al. (pediatric)**	2020	Germany	Yes	14 MHz	NR	NR	NR	NR	Inside the hyperechoic rim of the nerve	NR	59<br>58	59<br>58	R	8-12<br>13-17	33.15 (6.39)<br>62.82 (13.21)	142 (9)<br>173 (9)	16.21 (1.99)<br>20.90 (3.19)
**Niu et al.**	2020	China	Yes	8-12 MHz	NR	NR	NR	NR	Inside the hyperechoic rim of the nerve	NR	111	222	B	41.69 (15.80)	65.34 (11.06)	167.07 (8.31)	23.32 (2.86)

Article	Year	Country (ethnicity, if mentioned)	Diabetic patients excluded	MHz of probe	Experience of examiner	N of investigators	Position of patient during ultrasound examination	N of measurements taken to calculate mean CSA	Anatomic landmarks of the nerve used for tracing CSA	Tracing method used for CSA measurement	N of subjects	N of elbows	Side of mean CSA	Age in yrs<br><br>Mean with (SD or 95%CI) or [range]	Weight in kg<br><br>Mean with (SD or 95%CI) or [range]	Height in cm<br><br>Mean with (SD or 95%CI) or [range]	BMI in kg/m²<br><br>Mean with (SD or 95%CI) or [range]
**Schubert et al. (pediatric)**	2020	Germany	Yes	14 MHz	>5000 examinations	2, excellent inter- and intrarater variability	NR	NR	Inside the hyperechoic rim of the nerve	NR	58<br>58	58<br>58	R<br>R	2-4<br>5-7	.	.	15.9 (1.5)<br>15.2 (2.1)
**Tahmaz et al.**	2020	Germany	Yes	5-18MHz	Supervisor >8 years	supervised and validated by a neurologist, adequate inter- and intrarater variability	NR	NR	Inside the hyperechoic rim of the nerve	Freehand tracing	80	160	B	56.71 (22.8)	.	.	24.6 (4.0)
**Tandon et al.**	2020	India	Yes	7-12 MHz	> 10 years	2, excellent interrater reliability	Supine, arm by the side and the hand supinated	NR	Inside the hyperechoic rim of the nerve	NR	30	60	B	51.26 (9.62)	61.5 (5.99)	156.1 (9.48)	25.46 (2.50)
**Bedewi et al.**	2021	Saudi Arabia	No	4-18 MHz	19 years	1, and reviewed by a neurologist	Sitting with the arm on the thigh, extended elbow, slightly abducted	NR	NR	NR	20	38	B	32.9 (6.6)	59.7 (10.4)	156.6 (8.3)	24.3 (3.5)

[1]= main cohort of healthy patients (diagnostic accuracy studies, dating from 2013 to 2015, using (subgroups of this) cohort are excluded to prevent duplicate data); [2]= main cohort of healthy patients (diagnostic accuracy studies studies, dating from 2015 to 2017, using (subgroups of this) cohort are excluded to prevent duplicate data); .=missing information; [M]=only males; [F]=only females. B = both, D = dominant side; DBUN = deep branch of the ulnar nerve; FCU = flexor carpi ulnaris; L = left; ME = medial epicondyle; N = number; ND = non-dominant side; NR = not reported; R = right; SD = standard deviation; CSA = cross-sectional area

Table 5 Mean CSA measurements reported in included studies in adults

Study	Year	N of elbows	Side in one patient	Mean CSA value in mm2 (SD) or [range]
**Just distal to wrist crease**				
Girtler et al.	2012	40	B	4 [2-6]*
**Wrist (distal wrist crease, Guyon's canal)**				
Peeters et al.	2004	30	B	8.4 (4.2) [#]
Cartwright et al.	2007	60	B	5.9 (1.1)
Bayrak et al.	2010	42	B	4.0 [3.0 - 8.0]
Girtler et al.	2012	40	B	4 [2-6]*
Bathala et al.	2013	200	B	3.6 (0.5)
Cartwright et al.	2013	36	B	6.5 (1.7) [G]
Kerasnoudis et al.	2013	150	B	5.16 (1.03)
Sugimoto et al.	2013	120	B	4.1 (1.0)
Tagliafico et al.	2013	80	B	3.1 (1.0)
Won et al.	2013	194	B	4.3 (0.8) [#]
Yalcin et al.	2013	144	B	4.9 (0.6)
Jang et al.	2014	36	B	5.0 (0.5)
Kim et al.	2015	30	R	4.4 (0.6)
Reckelhoff et al.	2015	83	B	5.6 (1.8) [#]
Schreiber et al.	2015	36	B	6.1 (1.4)
Afsal et al.	2016	56	B	3.9 (0.8) [#]
Agirman et al.	2016	28	B	5.6 (0.8)
Arumugam et al.	2016	40	ND	4.12 (0.94)
Kang et al.	2016	20/40	?	5.03 (0.78)
Merola et al.	2016	140	B	4.82 (1.04)
Qrimli et al.	2016	185	B	5.0 (1.8) [#]
Yagci et al.	2016	60	B	6.18 (0.91)[F]
Yurdakul et al.	2016	62	B	4.86 (1.17)
Chen et al.	2017	200	B	4.86 (1.16)
Niu et al.	2017	18	B or R	3.1 (1.1)
Atan et al.	2018	50	L or R	3.61 (1.07)
Jiwa et al.	2018	37	L or R	4.82 (0.93)
Mulholland et al.	2018	51	.	5.3 (1.0)
Paluch et al. (1)	2018	39	L or R	3.23 [2-5]
Schreiber et al.	2018	36	B	6.5 (1.0) [#]
Chang et al.	2019	59	B	3.5 (1.4)
Druzhinin et al.	2019	44	B	4.2 (1.5) [#]
Singh et al.	2019	45	R	5.56 (1.22)
Choi et al.	2020	60	B	6.59 (1.17)
Niu et al.	2020	222	B	2.9 (0.6)
Tahmaz et al.	2020	160	B	5.0 (1.0)
Tandon et al.	2020	60	B	2.41 (0.51)
**2cm proximal to wrist crease**				
Kathirgamanathan et al.	2009	100	B	4.7 [1-15.1]
Girtler et al.	2012	40	B	4 [3-6]*
Won et al.	2013	194	B	4.9 (0.9) [#]
Jang et al.	2014	36	B	6.5 (0.8)
Mori et al.	2015	30	R	5.1 (1.0)
**4cm proximal to wrist crease**				
Niu et al.	2017	18	B or R	3.3 (0.8)
Niu et al.	2020	222	B	3.9 (0.8)
**6cm proximal to wrist crease**				
Chen et al.	2017	200	B	5.00 (1.26)
**Distal 1/3th of the forearm**				
Tagliafico et al. (2)	2008	34	B	6.66 (1.74)

Sugimoto et al.	2013	120	B	4.7 (1.0)
Agirman et al.	2016	28	B	5.9 (0.8)
Yagci et al.	2016	60	B	7.45 (0.94)[F]
**Mid-forearm, where the ulnar artery and nerve made contact**				
Cartwright et al. (2)	2007	60	B	6.3 (1.0)
Tagliafico et al.	2008	34	B	5.31 (1.43)
Yoon et al. (2)	2008	30	R	6.3 (1.0)
Hooper et al.	2011	64	B	6.29 (0.95)
Kerasnoudis et al.	2013	150	B	5.46 (1.26)
Pompe et al.	2013	73	B	3.2 (0.7)
Sugimoto et al.	2013	120	B	4.6 (0.8)
Won et al.	2013	194	B	6.3 (1.1) [#]
Yalcin et al.	2013	144	B	5.0 (0.6)
Boehm et al.	2014	56	L	5.2 (1.3)
Grimm et al. (1)	2014	16	B	6.0 (2.2)
Grimm et al. (2)	2014	42	B	6.1 (1.5)
Jang et al.	2014	36	B	7.3 (0.9)
Scheidl et al.	2014	34	L	5.5 (1.3)
Ellegaard et al.	2015	43	L	5.5 (1.1) [SD]
Kim et al.	2015	30	R	6.6 (1.2)
Mori et al.	2015	30	R	6.0 (1.7)
Schreiber et al.	2015	36	B	7.2 (1.7) [#]
Arumugam et al.	2016	40	ND	4.60 (1.06)
Dikici et al.	2016	266	B	6.1 (1.2)
Kang et al.	2016	20/40	?	6.85 (0.69)
Kose Ozlece et al.	2016	78	B	4.16 (1.0)
Merola et al.	2016	140	B	6.07 (1.42)
Qrimli et al.	2016	185	B	6.3 (1.7) [#]
Niu et al.	2017	18	B or R	4.4 (1.0)
Pelosi et al.	2017	7	L or R	6.03 (1.5)
Grimm et al.	2018	100	R	5.9 (1.4)
Mulroy et al.	2018	8	.	6.2 (1.4)
Pelosi et al.	2018	28	B	6.03 (1.1)
Schreiber et al.	2018	36	B	7.3 (1.5) [#]
Druzhinin et al.	2019	44	B	5.8 (1.9) [#]
Niu et al.	2020	222	B	4.6 (0.8)
Tahmaz et al.	2020	160	B	5.4 (1.0)
Bedewi et al.	2021	38	B	7.1 (1.7)
**2cm proximal to the contact point of ulnar artery and nerve**				
Kathirgamanathan et al.	2009	100	B	6.2 [4-14.2]
**Proximal 1/3rd of the forearm**				
Eichenberger et al.	2009	17	ND	6.2 (1.0)
Agirman et al.	2016	28	B	6.1 (1.0)
**Alongside muscle belly of the flexor carpi ulnaris**				
Niu et al.	2017	18	B or R	4.8 (1.0)
Niu et al.	2020	222	B	4.5 (0.8)
**3-5cm distal to tip of the medial epicondyle**				
Ayromlou et al.	2012	35	?	4 (2.9) [SD]
Bathala et al.	2013	200	B	4.1 (0.6)
Ellegaard et al.	2015	43	L	5.5 (1.3) [SD]
Roodt et al. (flexion)	2015	25	D	4.9 (1.0)
Roodt et al. (extension)	2015	25	D	5.2 (0.9)
**Outlet of the FCU**				
Niu et al.	2017	18	B or R	5.1 (0.7)
**Cubital tunnel outlet (1-2 cm distal to medial epicondyle, between the two heads of the FCU muscle)**				
Cartwright et al.	2007	60	B	6.4 (1.1)

Yoon et al. (1) (extension)	2008	40	B	8
Yoon et al. (1) (flexion)	2008	40	B	5
Elias et al.	2009	20	?	5.19 (1.31)
Kutlay et al. (extension)	2009	40	B	6.6 (1.4) [#]
Kutlay et al. (flexion)	2009	40	B	5.7 (1.3) [#]
Bayrak et al.	2010	42	B	8.0 [5.0-12.0]
Girtler et al.	2012	40	B	8 [5-10]*
Pazzaglia et al.	2013	37	?	6.5 (1.2)
Pompe et al.	2013	73	B	5.1 (1.6)
Won et al.	2013	194	B	7.4 (1.4) [#]
Yalcin et al.	2013	144	B	5.6 (1.0)
Jang et al.	2014	36	B	8.6 (1.2)
Yalcin et al.	2014	38	B	5.6 (1.1) [&]
Ellegaard et al.	2015	43	L	5.9 (1.5) [SD]
Ghanei et al.	2015	44	L or R	7.8 (1.4)
Gupta et al.	2016	60	B	4.00 (0.13)
Kang et al.	2016	20/40	?	7.37 (0.82)
Chen et al.	2017	200	B	6.26 (1.29)
Niu et al.	2017	18	B or R	5.1 (1.4)
Rayegani et al.	2019	34	L or R	7.57 (1.34)
Niu et al.	2020	222	B	4.9 (0.9)
**Medial epicondyle**				
Jacob et al.	2004	400	B	7.9 (3.1)
Cartwright et al.	2007	60	B	6.5 (0.9)
Thoirs et al.	2008	107	B	8.2 (3.2)
Yoon et al. (2)	2008	30	R	6.6 (1.0)
Jain et al.	2009	60	B	8.5 (3.5)
Kutlay et al. (extension)	2009	40	B	6.9 (1.9) [#]
Kutlay et al. (flexion)	2009	40	B	5.6 (1.6) [#]
Bayrak et al.	2010	42	B	9.0 [5.0 - 12.0]
Hooper et al.	2011	64	B	7.61 (0.78)
Ayromlou et al.	2012	35	?	4 (2.9) [SD]
Boom & Visser	2012	37	B	6.17 (1.56)
Bathala et al.	2012	200	B	4.7 (0.6)
Childs et al. (European)	2012	48	B	7.0 (2.0)
Childs et al. (Asian)	2012	24	B	7.0 (2.0)
Cartwright et al.	2013	36	B	10.6 (3.0) [G]
Kerasnoudis et al.	2013	150	B	5.33 (1.4)
Pompe et al.	2013	73	B	5.3 (1.6)
Sugimoto et al.	2013	120	B	6.7 (1.9)
Yalcin et al.	2013	144	B	6.2 (1.1)
Boehm et al.	2014	56	L	7.2 (2.1)
Jang et al.	2014	36	B	8.7 (1.0)
Scheidl et al.	2014	34	L	7.6 (2.1)
Yalcin et al.	2014	38	B	5.7 (1.1)
Ellegaard et al.	2015	43	L	6.8 (1.5) [SD]
Roodt et al. (flexion)	2015	25	D	5.2 (1.0)
Roodt et al. (extension)	2015	25	D	5.4 (1.0)
Schreiber et al.	2015	36	B	7.6 (2.4) [#]
Afsal et al.	2016	56	B	5.3 (1.6)
Agirman et al.	2016	28	B	7.7 (1.1)
Merola et al.	2016	140	B	5.94 (1.82)
Qrimli et al.	2016	185	B	6.9 (2.3) [#]
Yagci et al.	2016	60	B	8.21 (1.22)[F]
Chen et al.	2017	200	B	5.87 (1.49)
Grimm et al.	2018	100	R	8.7 (2.0)
Paluch et al. (2)	2018	38	L or R	6.47 [3-9]
Lothet et al.	2019	30	B	9.27 (2.25) [E]
Rayegani et al.	2019	34	L or R	8.27 (1.74)
Singh et al.	2019	45	R	6.50 (1.41)
Tahmaz et al.	2020	160	B	7.1 (1.8)
Tandon et al.	2020	60	B	3.11 (0.81)

**Cubital tunnel (including maximal CSA measurements between cubital tunnel inlet and outlet)**				
Wiesler et al.	2006	60	B	6.5 (1.0)
Ozturk et al.	2008	212	B	6.6 (1.7)
Tagliafico et al. (1)	2008	50	.	6.8 (1.2)
Elias et al.	2009	20	L or R	6.84 (1.92)
Bayrak et al.	2010	42	B	9.0 [7.0 - 12.0]
Ayromlou et al.	2012	35	B	5 (2.9) [SD]
Girtler et al.	2012	40	B	8 [5-10]*
Frade et al.	2013	92	B	6.7 (2.2)
Pompe et al.	2013	73	B	5.9 (1.0)
Scheidl et al.	2013	87	B	7.6 (1.7)
Tagliafico et al.	2013	80	B	5.9 (3.0)
Ghanei et al.	2015	44	L or R	7.2 (1.7)
Cheng et al.	2016	100	B	6.7 (1.3)
Gupta et al.	2016	60	B	4.55 (0.02) [#]
Niu et al.	2017	18	B or R	6.2 (1.9)
Chen et al.	2018	29	B	9.4 (2.7)
Niu et al.	2020	222	B	5.6 (1.1)
**Cubital tunnel inlet (1-2 cm proximal to medial epicondyle)**				
Cartwright et al.	2007	60	B	6.7 (1.1)
Yoon et al. (1) (extension)	2008	40	B	7
Yoon et al. (1) (flexion)	2008	40	B	6
Kathirgamanathan et al.	2009	100	B	6.2 [4-14.2]
Kutlay et al. (extension)	2009	40	B	6.4 (1.6) [#]
Kutlay et al. (flexion)	2009	40	B	5.7 (1.6) [#]
Bayrak et al.	2010	42	B	8.0 [5.0-12.0]
Girtler et al.	2012	40	B	8 [5-11]*
Visser et al.	2012	25	L or R	5.32 (1.46)
Pompe et al.	2013	73	B	4.2 (1.1)
Won et al.	2013	194	B	7.2 (1.3) [#]
Yalcin et al.	2013	144	B	6.2 (1.1) [&]
Jang et al.	2014	36	B	7.7 (1.3)
Yalcin et al.	2014	38	B	5.7 (1.1)
Ellegaard et al.	2015	43	L	6.5 (1.1) [SD]
Ghanei et al.	2015	44	L or R	7.8 (1.4)
Kim et al.	2015	30	R	7.5 (1.3)
Kang et al.	2016	20/40	?	7.29 (1.02)
Bedewi et al.	2017	100	B	6.54 (1.67) [M]
Chen et al.	2017	200	B	6.38 (1.38) [#]
Fink et al.	2017	73	D	10.0 (2.5) [&]
Niu et al.	2017	18	B or R	4.7 (1.2)
Chen et al.	2018	29	B	9.1 (2.2)
Rayegani et al.	2019	34	L or R	7.52 (1.55)
Niu et al.	2020	222	B	4.9 (0.9)
**4-5 cm proximal to tip of the medial epicondyle**				
Ayromlou et al.	2012	35	?	4 (1.5) [SD]
Bathala et al.	2012	44	B	4.83 (1.0)
Bathala et al.	2013	200	B	4.4 (0.6)
Ellegaard et al.	2015	43	L	5.4 (1.1) [SD]
Roodt et al. (flexion)	2015	25	D	5.1 (0.9)
Roodt et al. (extension)	2015	25	D	5.3 (0.8)
Gupta et al.	2016	60	B	4.08 (0.14)
Niu et al.	2017	18	B or R	4.4 (1.3)
Niu et al.	2020	220	B	4.4(0.8)
**Mid-upper arm**				
Cartwright et al.	2007	60	B	6.1 (0.9) [G]
Yoon et al. (2)	2008	30	R	6.2 (1.0)
Elias et al.	2009	20	?	7.05 (1.66)

Frade et al.	2013	92	B	5.9 (1.8)
Pompe et al.	2013	73	B	3.8 (0.9)
Scheidl et al.	2013	87	B	6.33
Sugimoto et al.	2013	120	B	4.8 (1.0)
Won et al.	2013	194	R	5.8 (1.0) [#]
Boehm et al.	2014	56	L	6.3 (1.7)
Grimm et al. (1)	2014	16	B	7.7 (3.3)
Grimm et al. (2)	2014	42	B	7.7 (2.3)
Jang et al.	2014	36	B	9.0 (1.2)
Scheidl et al.	2014	34	L	6.5 (1.6)
Arumugam et al.	2016	40	ND	5.88 (1.56)
Kang et al.	2016	20/40	?	6.76 (0.87)
Merola et al.	2016	140	B	7.31 (1.79)
Qrimli et al.	2016	185	B	6.9 (2.3) [#]
Chen et al.	2017	200	B	5.60 (1.34)
Niu et al.	2017	18	B or R	4.0 (0.9)
Pelosi et al.	2017	7	L or R	5.67 (0.67)
Grimm et al.	2018	100	R	7.0 (1.2)
Mulroy et al.	2018	8	L or R	6.4 (1.1) [#]
Pelosi et al.	2018	28	B	5.96 (1.1)
Druzhinin et al.	2019	44	L	6.4 (2.0)
Niu et al.	2020	222	B	4.4 (0.9)
Tahmaz et al.	2020	160	B	6.5 (1.4)
**Axilla**				
Cartwright et al.	2007	60	B	6.2 (1.1) [E]
Bathala et al.	2013	200	B	4.3 (0.5)
Kerasnoudis et al.	2013	150	B	6.53 (1.82)
Niu et al.	2020	222	B	4.5 (0.9)
**DBUN: Directly after separation from the ulnar nerve**				
Corroler et al.	2013	20	?	1.6 [1.1-2.2]
Riegler et al.	2017	20	B	1.8 (0.5)
**DBUN: At hook of hamate level**				
Choi et al.	2020	60	B	1.51 (0.24)
**DBUN: At metacarpal base level**				
Riegler et al.	2017	20	B	1.6 (0.4)
Choi et al.	2020	60	B	1.35 (0.25)
**Ulnar nerve at the level of the branch point of the DUCN**				
Kim et al.	2018	60	B	6.1 [4.1-8.1]
**DUCN: 1 cm distal to its origin**				
Kim et al.	2018	60	B	1.5 [0.6-2.3]
**Ulnar nerve at the level of the branch point of the PUCN**				
Kim et al.	2018	60	B	6.7 [5.0-10.0]
**PUCN: 1 cm distal to its origin**				
Kim et al.	2018	47	B	0.3 [0.2-0.6]
**Ulnar nerve at the level of the branch point of the SSB**				
Kim et al.	2018	60	B	5.2 [4.0-7.2]
**SSB: 1cm distal to its origin**				
Kim et al.	2018	60	B	3.9 [2.4-6.5]

*=median instead of mean; [SD] = SD calculated from 95% confidence interval using the formula described in the methods; [#]= combined mean and standard deviation is calculated using the formula

described in the methods ; [&]= mean of two measurements in the same patients (different assessors or 1 cm difference in location); [E]=extremes of BMI; [G]=geriatrics; [M]=only males; [F]=only females; B = both sides; CI = confidence interval; CSA = cross-sectional area; FCU = flexor carpi ulnaris; D = dominant side; DBUN = deep branch ulnar nerve; DUCN = dorsal ulnar cutaneous nerve; L = left side; ME = medial epicondyle; N = number; ND = non-dominant; PUCN = palmar ulnar cutaneous nerve; R = right side; SD = standard deviation; SSB = superficial sensory branch

Table 6 Mean CSA measurements reported in included studies in children

Study	Year	Age range	Mean age	N of elbows	Side	Mean CSA value in mm2 (SD)
**Distal wrist crease (Guyon's canal)**						
Cartwright et al.	2013	0-3	1.2 (0.7)	4	B	2.5 (0.7)
		4-6	4.8 (0.9)	4	B	4.5 (0.7)
		7-11	8.8 (1.6)	4	B	3.5 (0.7)
		12-16	13.8 (1.4)	12	B	5.8 (1.5)
Druzhinin et al.	2019	2-4	3.27 (0.79)	22	B	2.5 (0.7) #
		5-7	5.61 (0.80)	30	B	3.0 (1.1) #
		8-10	8.47 (1.59)	20	B	3.3 (1.6) #
		11-13	11.3 (1.22)	14	B	4.2 (1.6) #
		14-16	14.8 (1.57)	14	B	4.9 (1.6) #
**Mid-forearm, where the ulnar artery and nerve made contact**						
Yiu et al.	2015	.	11.3 (4.4)	29	D	3.3 (0.9)
Hobbelink et al.	2018	.	8.2 (4.0)	5	D	2.9 (0.8)
Druzhinin et al.	2019	2-4	3.27 (0.79)	22	B	3.0 (0.6) #
		5-7	5.61 (0.80)	30	B	3.4 (0.9) #
		8-10	8.47 (1.59)	20	B	4.4 (1.3) #
		11-13	11.3 (1.22)	14	B	4.9 (1.4) #
		14-16	14.8 (1.57)	14	B	5.7 (1.8) #
Grimm et al.	2020	8-12	.	59	R	3.95 (1.24)
		13-17	.	58	R	5.50 (1.68)
Schubert et al.	2020	2-4	.	58	R	3.03 (1.01)
		5-7	.	58	R	3.66 (1.02)
**Medial epicondyle**						
Cartwright et al.	2013	0-3	1.2 (0.7)	4	B	3.5 (0.7)
		4-6	4.8 (0.9)	2	B	4.0
		7-11	8.8 (1.6)	6	B	5.0 (2.0)
		12-16	13.8 (1.4)	10	B	7.2 (1.3)
Yiu et al.	2015	.	11.3 (4.4)	29	D	3.8 (1.3)
Hobbelink et al.	2018	.	8.2 (4.0)	5	D	2.8 (0.8)
Grimm et al.	2020	8-12	.	59	R	2.93 (1.56)
		13-17	.	58	R	5.26 (2.35)
Schubert et al.	2020	2-4	.	58	R	2.40 (1.17)
		5-7	.	58	R	3.16 (1.32)
**Mid-upper arm**						
Hobbelink et al.	2018	.	8.2 (4.0)	5	D	3.0 (0.7)
Druzhinin et al.	2019	2-4	3.27 (0.79)	22	B	3.7 (2.6) #
		5-7	5.61 (0.80)	30	B	4.3 (1.0) #
		8-10	8.47 (1.59)	20	B	5.0 (1.3) #
		11-13	11.3 (1.22)	14	B	4.9 (1.5) #
		14-16	14.8 (1.57)	14	B	6.1 (2.6) #
Grimm et al.	2020	8-12	.	59	R	4.75 (1.37)
		13-17	.	58	R	6.66 (2.07)
Schubert et al.	2020	2-4	.	58	R	3.78 (1.09)
		5-7	.	58	R	4.24 (1.06)

B = both sides; D = dominant side; N = number; SD = standard deviation; CSA = cross-sectional area .

All data by Druzhinin et al. combined using the formula described in the method section.

Table 7 Mean CSA measurements at different anatomical locations of the ulnar nerve, separate for men and women

Study	Year	Men		Women	
		N elbows	Mean CSA (SD)	N elbows	Mean CSA (SD)
**Wrist (distal wrist crease, Guyon's canal)**					
Peeters et al.	2004	16	9.6 (2.5)	14	7.0 (2.79)
Cartwright et al.	2007	22	6.4 (1.2)	28	5.6 (0.8)
Bathala et al.	2013	50	3.8 (0.5)	50	3.4 (0.4)
Kerasnoudis et al.	2013	90	5.38 (0.91)	60	4.83 (1.19)
Sugimoto et al.	2013	58	4.5 (0.9)	62	3.8 (0.9)
Yalcin et al.	2013	94	5.2 (0.8)	100	4.7 (0.5)
Reckelhoff et al.	2015	51	6.0 (2.0)	31	5.0 (1.0)
Chen et al.	2017	100	4.80 (1.16)	100	4.93 (1.29)
Choi et al.	2020	31	6.9 (1.2)	28	6.0 (0.9)
Niu et al.	2020	112	3.1 (0.6)	110	2.8 (0.5)
**4cm proximal to wrist crease**					
Niu et al.	2020	112	4.1 (0.8)	110	3.8 (0.9)
**6cm proximal to wrist crease**					
Chen et al.	2017	100	5.10 (1.27)	100	4.90 (1.17)
**Distal 1/3th of the forearm**					
Sugimoto et al.	2013	58	5.0 (0.9)	62	4.4 (1.0)
**Mid-forearm, where the ulnar artery and nerve made contact**					
Cartwright et al.	2007	22	6.5 (1.2)	28	6.1 (0.7)
Hooper et al.	2011	26	6.41 (1.03)	38	6.20 (0.91)
Kerasnoudis et al.	2013	90	5.32 (1.33)	60	5.58 (1.24)
Sugimoto et al.	2013	58	4.6 (0.8)	62	4.6 (0.9)
Yalcin et al.	2013	94	5.2 (0.7)	100	4.7 (0.5)
Dikici et al.	2016	92	6.10 (1.30)	141	6.12 (1.24)
Grimm et al.	2018	55	6.0 (1.3)	45	5.7 (1.3)
Niu et al.	2020	112	4.7 (0.8)	110	4.4 (0.8)
Bedewi et al.	2021	38	7.1	.	.
**Proximal 1/3rd of the forearm**					
Niu et al.	2020	112	4.6 (0.9)	110	4.4 (0.7)
**3cm distal to tip of the medial epicondyle**					
Bathala et al.	2013	50	4.2 (0.6)	50	3.9 (0.5)
**Cubital tunnel outlet (1-1,5 cm distal to medial epicondyle, between the two heads of the FCU muscle)**					
Cartwright et al.	2007	22	6.9 (1.4)	28	6.1 (0.8)
Yalcin et al.	2013	94	6.0 (1.0)	100	5.2 (0.8)
Chen et al.	2017	100	6.30 (1.36)	100	6.22 (1.30)
Niu et al.	2020	112	5.1 (0.9)	110	4.7 (0.8)
**Medial epicondyle**					
Jacob et al.	2004	154	8.3 (3.3)	246	7.6 (2.9)
Cartwright et al.	2007	22	7.2 (1.3)	28	6.2 (1.3)
Hooper et al.	2011	13	8.07 (0.55)	19	7.30 (0.77)
Bathala et al.	2013	50	4.8 (0.6)	50	4.5 (0.6)
Kerasnoudis et al.	2013	90	5.55 (1.75)	60	5.13 (0.6)
Sugimoto et al.	2013	58	7.0 (1.2)	62	6.4 (1.6)
Yalcin et al.	2013	94	6.6 (1.1)	100	5.8 (1.0)
Chen et al.	2017	100	6.18 (1.49)	100	5.38 (1.30)
Grimm et al.	2018	55	8.7 (1.9)	45	8.7 (1.9)

Niu et al.	2020	112	5.8 (1.1)	110	5.4 (1.0)
**Cubital tunnel (including maximal CSA measurements between cubital tunnel inlet and outlet)**					
Ozturk et al.	2008	120	6.7 (1.8)	92	6.5 (1.7)
**Cubital tunnel inlet (just proximal to medial epicondyle)**					
Cartwright et al.	2007	22	7.3 (1.1)	28	6.4 (1.3)
Yalcin et al.	2013	94	6.2 (1.0)	100	5.4 (0.9)
Chen et al.	2017	100	6.42 (1.38)	100	6.35 (1.33)
Niu et al.	2020	112	5.1 (0.9)	110	4.6 (0.8)
Bedewi et al.	2021	38	6.54 (1.67)	.	.
**4-5 cm proximal to tip of the medial epicondyle**					
Bathala et al.	2013	50	4.6 (0.6)	50	4.1 (0.6)
Niu et al.	2020	112	4.6 (0.8)	110	4.1 (0.8)
**Mid-upper arm**					
Cartwright et al.	2007	22	6.8 (1.3)	28	5.9 (0.7)
Sugimoto et al.	2013	58	5.1 (1.1)	62	4.5 (0.7)
Chen et al.	2017	100	5.94 (1.38)	100	5.36 (1.31)
Grimm et al.	2018	55	7.0 (1.3)	45	7.0 (1.1)
Niu et al.	2020	112	4.8 (0.8)	110	4.1 (0.9)
**Axilla**					
Cartwright et al.	2007	22	6.8 (1.7)	28	5.9 (6.3)
Bathala et al.	2013	50	4.5 (0.5)	50	4.1 (0.6)
Kerasnoudis et al.	2013	90	6.88 (2.02)	60	6.2 (1.47)
Niu et al.	2020	112	4.8 (0.9)	110	4.1 (0.8)

B = both sides; D = dominant side; N = number; SD = standard deviation; CSA = cross-sectional area .

All data by Druzhinin et al. combined using the formula described in the method section.

Table 8 Mean CSA measurements at different anatomical locations of the ulnar nerve for different age groups

**Study**	**Year**				
**Wrist crease**					
Bathala et al.	2013	Age<br>N<br>CSA (SD)	18 – 40<br>years<br>50<br>3.59 (0.64)	>40 years<br>30<br>3.88 (0.47)	
Kerasnoudis et al.	2013	Age<br>N<br>CSA (SD)	20 – 39<br>years<br>20<br>5.41 (0.99)	50 – 59<br>years<br>30<br>4.8 (1.31)	>60 years<br>25<br>5.25 (0.51)
Niu et al.	2020	Age<br>N<br>CSA (SD)	<30 years<br>31<br>2.9 (0.5)	30 – 49<br>years<br>40<br>2.9 (0.6)	≥ 50 years<br>40<br>3.0 (0.6)
**4cm proximal to wrist crease**					
Niu et al.	2020	Age<br>N<br>CSA (SD)	<30 years<br>31	30 – 49<br>years<br>40	≥ 50 years<br>40
**Mid-forearm**					
Kerasnoudis et al.	2013	Age in<br>N<br>CSA (SD)	20 – 39<br>years<br>20<br>5.43 (1.5)	50 – 59<br>years<br>30<br>5.1 (1.15)	>60 years<br>25<br>6.5 (0.57)
Niu et al.	2020	Age in<br>N<br>CSA (SD)	<30 years<br>31<br>4.2 (0.8)	30 – 49<br>years<br>40<br>4.7 (0.7)	≥ 50 years<br>40<br>4.7 (0.8)
**Proximal 1/3rd of the forearm**					
Niu et al.	2020	Age in<br>N<br>CSA (SD)	<30 years<br>31<br>4.3 (0.8)	30 – 49<br>years<br>40<br>4.6 (0.9)	≥ 50 years<br>40<br>4.6 (1.0)
**3cm distal to tip of the medial epicondyle**					
Bathala et al.	2013	Age<br>N<br>CSA (SD)	18 – 40<br>years<br>50<br>3.94 (0.74)	>40 years<br>30<br>4.2 (0.47)	
**Cubital tunnel outlet**					
Niu et al.	2020	Age<br>N<br>CSA (SD)	<30 years<br>31<br>4.7 (0.8)	30 – 49<br>years<br>40<br>5.0 (0.8)	≥ 50 years<br>40<br>4.9 (1.0)
**Medial epicondyle**					
Jacob et al.	2004	Age<br>N<br>CSA (SD)	<40 years<br>142<br>7.5 (3.1)	40 – 60<br>years<br>176<br>8.3 (2.9)	>60 years<br>62<br>7.6 (3.4)
Bathala et al.	2013	Age<br>N<br>CSA (SD)	18 – 40<br>years<br>50<br>4.46 (0.75)	>40 years<br>30<br>4.95 (0.55)	
Kerasnoudis et al.	2013	Age in<br>N<br>CSA (SD)	20 – 39<br>years<br>20	50 – 59<br>years<br>30	>60 years<br>25<br>5.75 (0.95)

			4.83 (0.71)	6.2 (2.1)	
Niu et al.	2020	Age<br>N<br>CSA (SD)	<30 years<br>31<br>5.2 (1.0)	30 – 49 years<br>40<br>5.9 (1.0)	≥ 50 years<br>40<br>5.8 (1.1)
**Cubital tunnel inlet**					
Niu et al.	2020	Age<br>N<br>CSA (SD)	<30 years<br>31<br>4.5 (0.8)	30 – 49 years<br>40<br>5.0 (0.9)	≥ 50 years<br>40<br>5.0 (0.9)
**4-5 cm proximal to tip of the medial epicondyle**					
Bathala et al.	2013	Age<br>N<br>CSA (SD)	18 – 40 years<br>50<br>4.21 (0.63)	>40 years<br>30<br>4.53 (0.60)	
Niu et al.	2020	Age<br>N<br>CSA (SD)	<30 years<br>31<br>4.2 (0.8)	30 – 49 years<br>40<br>4.4 (0.8)	≥ 50 years<br>40<br>4.6 (0.9)
**Mid-upper arm**					
Niu et al.	2020	Age in<br>N<br>CSA (SD)	<30 years<br>31<br>4.2 (0.8)	30 – 49 years<br>40<br>4.5 (0.9)	≥ 50 years<br>40<br>4.5 (1.0)
**Axilla**					
Bathala et al.	2013	Age<br>N<br>CSA (SD)	18 – 40 years<br>50<br>4.21 (0.58)	>40 years<br>30<br>4.44 (0.64)	
Kerasnoudis et al.	2013	Age<br>N<br>CSA (SD)	20 – 39 years<br>20<br>6.83 (1.46)	50 – 59 years<br>30<br>7.1 (2.35)	>60 years<br>25<br>6.5 (1.29)
Niu et al.	2020	Age<br>N<br>CSA (SD)	<30 years<br>31<br>4.2 (0.8)	30 – 49 years<br>40<br>4.5 (0.9)	≥ 50 years<br>40<br>4.6 (1.0)

B = both sides; D = dominant side; N = number; SD = standard deviation; CSA = cross-sectional area .

Table 9 Subgroup analyses

Position	Subgroup		N	Mean CSA (95%CI)	p-value
**Wrist crease**	Hz	<15 MHz	18	4.56 (4.08-5.03)	**0.048**
		≥15 MHz	15	5.17 (4.79-5.55)	
	Diabetics	Specifically excluded	21	4.86 (4.30-5.41)	0.947
		Unknown or not excluded	12	4.83 (4.34-5.32)	
	Position	Flexion	13	4.68 (4.15-5.20)	
		Extension	17	4.95 (4.33-5.58)	0.798
		Unknown	3	4.83 (3.72-5.93)	
	Asian	Asian population	14	4.25 (3.75-4.74)	**0.001**
		Other	19	5.26 (4.91-5.61)	
	Gender	Female	10	4.73 (4.05-5.41)	0.150
		Male	10	5.47 (4.72-6.21)	
**Proximal of wrist crease**	Hz	<15 MHz	6	4.74 (4.03-5.44)	**0.004**
		≥15 MHz	3	6.67 (5.57-7.77)	
	Diabetics	Specifically excluded	5	5.75 (4.54-6.96)	0.325
		Unknown or not excluded	4	4.90 (3.73-6.08)	
	Position	Flexion	4	5.14 (4.71-5.56)	0.644
		Extension	0	*NA*	
		Unknown	5	5.56 (3.81-7.30)	
	Asian	Asian population	6	4.74 (4.03-5.44)	**0.004**
		Other	3	6.67 (5.57-7.77)	
	Gender	Female	2	*NA*	
		Male	2	*NA*	
**Mid-forearm**	Hz	<15 MHz	15	5.92 (5.45-6.40)	0.493
		≥15 MHz	18	5.68 (5.15-6.21)	
	Diabetics	Specifically excluded	18	5.76 (5.34-6.18)	0.910
		Unknown or not excluded	16	5.80 (5.25-6.35)	
	Position	Flexion	11	5.77 (5.03-6.50)	0.996
		Extension	4	5.82 (4.83-6.81)	
		Unknown	19	5.78 (5.37-6.18)	
	Asian	Asian population	11	5.87 (5.22-6.53)	0.731
		Other	23	5.74 (5.33-6.14)	
	Gender	Female	8	5.42 (4.89-5.94)	0.650
		Male	8	5.58 (5.13-6.02)	
**Proximal forearm**	Hz	<15 MHz	6	*NA*	
		≥15 MHz	2	*NA*	
	Diabetics	Specifically excluded	4	4.92 (4.09-5.75)	0.750
		Unknown or not excluded	4	5.14 (4.11-6.16)	
	Position	Flexion	6	*NA*	
		Extension	1	*NA*	
		Unknown	2	*NA*	
	Asian	Asian population	3	4.42 (4.06-4.77)	**0.006**
		Other	5	5.42 (4.80-6.04)	
	Gender	Female	2	*NA*	
		Male	2	*NA*	
**Cubital tunnel outlet**	Hz	<15 MHz	12	6.24 (5.36-7.11)	0.755
		≥15 MHz	4	6.07 (5.56-6.59)	
	Diabetics	Specifically excluded	6	6.15 (4.94-7.36)	0.945
		Unknown or not excluded	11	6.20 (5.66-6.73)	
	Position	Flexion	9	6.12 (4.82-7.41)	0.845
		Extension	4	6.05 (5.53-6.57)	
		Unknown	5	6.45 (5.22-7.68)	
	Asian	Asian population	7	6.23 (5.05-7.42)	0.883
		Other	10	6.14 (5.63-6.64)	
	Gender	Female	4	5.55 (4.87-6.22)	0.316
		Male	4	6.04 (5.35-6.73)	
**Cubital tunnel**	Hz	<15 MHz	9	6.13 (5.36-6.91)	0.097
		≥15 MHz	6	7.03 (6.31-7.74)	
	Diabetics	Specifically excluded	8	6.12 (5.25-6.99)	0.133

		Unknown or not excluded	7	6.92 (6.34-7.51)	
	Position	Flexion	10	6.36 (5.41-7.32)	0.589
		Extension	0	*NA*	
		Unknown	5	6.77 (5.67-7.86)	
	Asian	Asian population	5	6.44 (5.41-7.48)	0.817
		Other	10	6.57 (6.23-6.91)	
	Gender	Female	1	*NA*	
		Male	1	*NA*	
**Medial epicondyle**	Hz	<15 MHz	19	6.67 (5.88-7.47)	0.581
		≥15 MHz	16	6.94 (6.44-7.44)	
	Diabetics	Specifically excluded	19	6.79 (5.96-7.62)	0.977
		Unknown or not excluded	17	6.81 (6.31-7.30)	
	Position	Flexion	16	6.38 (5.57-7.20)	0.164
		Extension	7	6.47 (5.98-6.95)	
		Unknown	14	7.24 (6.53-7.94)	
	Asian	Asian population	9	6.47 (5.14-7.80)	0.533
		Other	27	6.92 (6.38-7.47)	
	Gender	Female	10	6.22 (5.60-6.83)	0.241
		Male	10	6.81 (6.04-7.58)	
**Cubital tunnel inlet**	Hz	<15 MHz	14	6.90 (6.16-7.63)	0.333
		≥15 MHz	3	6.52 (6.32-6.72)	
	Diabetics	Specifically excluded	7	6.49 (5.40-7.57)	0.407
		Unknown or not excluded	11	7.01 (6.43-7.59)	
	Position	Flexion	9	7.25 (6.70-7.80)	**0.003**
		Extension	3	6.09 (5.72-6.45)	
		Unknown	7	6.48 (5.45-7.51)	
	Asian	Asian population	10	6.66 (5.84-7.48)	0.544
		Other	8	6.93 (6.29-7.70)	
	Gender	Female	4	5.67 (4.85-6.49)	0.344
		Male	4	6.24 (5.40-7.07)	
**Distal upper arm**	Hz	<15 MHz	8	*NA*	
		≥15 MHz	0	*NA*	
	Diabetics	Specifically excluded	5	4.49 (4.15-4.83)	0.536
		Unknown or not excluded	3	4.74 (4.02-5.46)	
	Position	Flexion	6	*NA*	
		Extension	1	*NA*	
		Unknown	2	*NA*	
	Asian	Asian population	4	4.30 (4.07-4.53)	**0.029**
		Other	4	4.87 (4.42-5.32)	
	Gender	Female	2	*NA*	
		Male	2	*NA*	
**Mid-upper arm**	Hz	<15 MHz	11	6.19 (5.43-6.95)	0.995
		≥15 MHz	14	6.20 (5.61-6.78)	
	Diabetics	Specifically excluded	14	6.25 (5.67-6.84)	0.803
		Unknown or not excluded	11	6.12 (5.29-6.95)	
	Position	Flexion	8	5.80 (5.09-6.50)	0.255
		Extension	0	*NA*	
		Unknown	16	6.37 (5.68-7.06)	
	Asian	Asian population	9	5.82 (5.10-6.54)	0.212
		Other	16	6.42 (5.82-7.02)	
	Gender	Female	5	5.37 (4.45-6.28)	0.391
		Male	5	5.91 (5.07-6.76)	
**Axilla**	Hz	<15 MHz	2	*NA*	
		≥15 MHz	2	*NA*	
	Diabetics	Specifically excluded	1	*NA*	
		Unknown or not excluded	3	*NA*	
	Position	Flexion	2	*NA*	
		Extension	0	*NA*	
		Unknown	2	*NA*	
	Asian	Asian population	2	*NA*	
		Other	2	*NA*	

Gender	Female	4	4.88 (4.05-5.71)	0.179
	Male	4	5.69 (4.85-6.52)	

95% CI = 95% confidence interval of the mean CSA value; CSA = cross-sectional area; N = number; NA = comparison is not applicable due to too little studies (two studies or less) in at least one of the subgroups. P values < 0.05 were considered significant.

Fig. 2 Forest plots of the mean CSA at different anatomical levels

A. Wrist crease

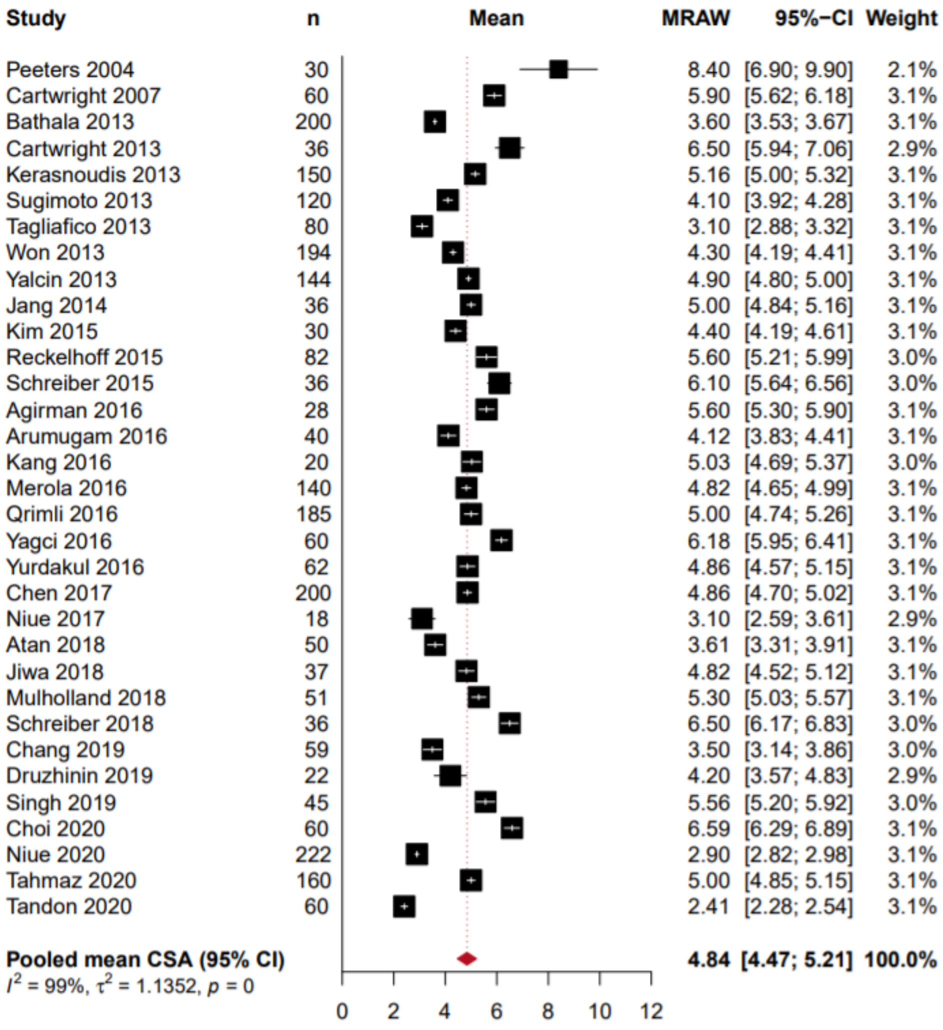

B. Proximal of wrist crease

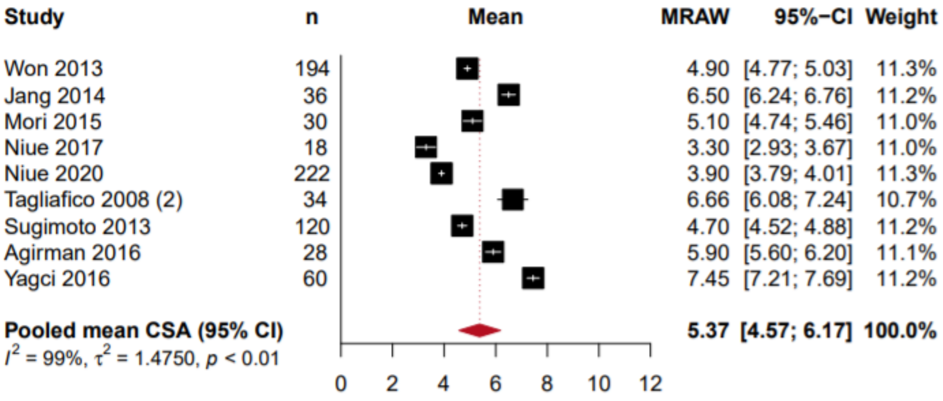

C. Mid-forearm

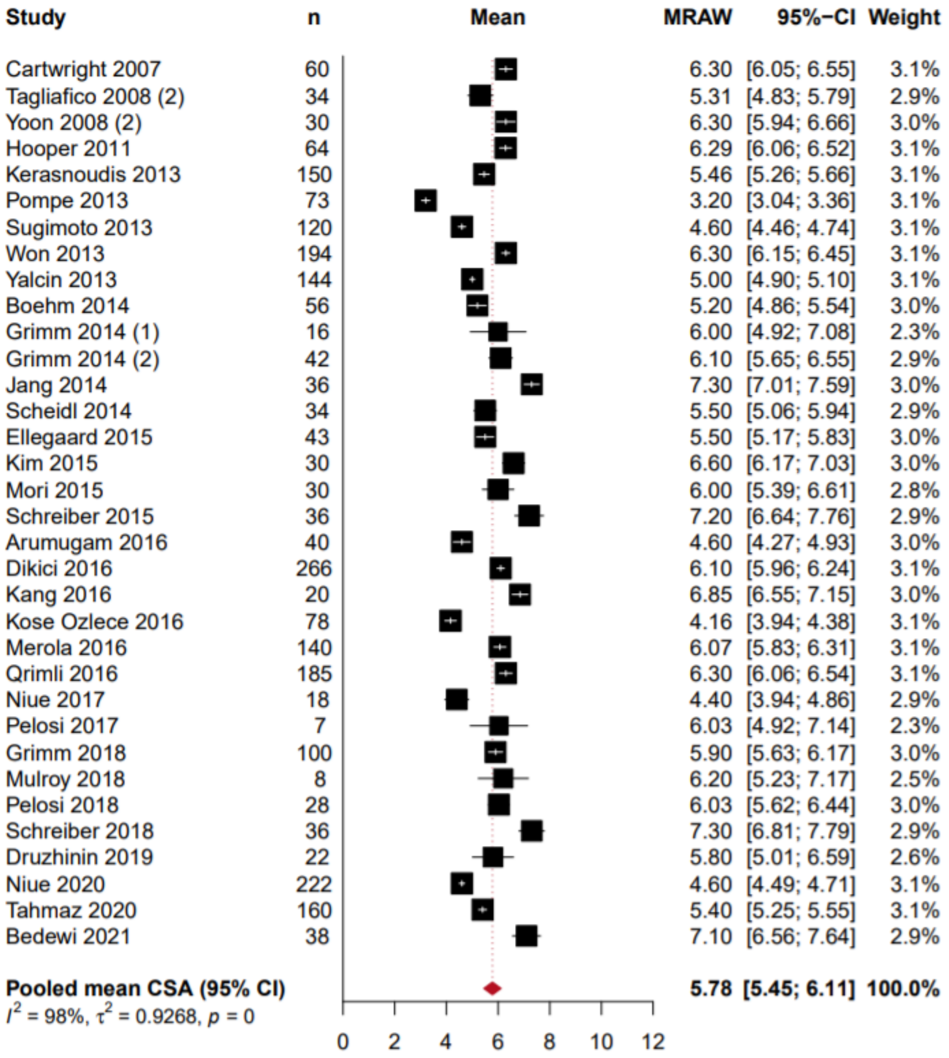

D. Proximal forearm

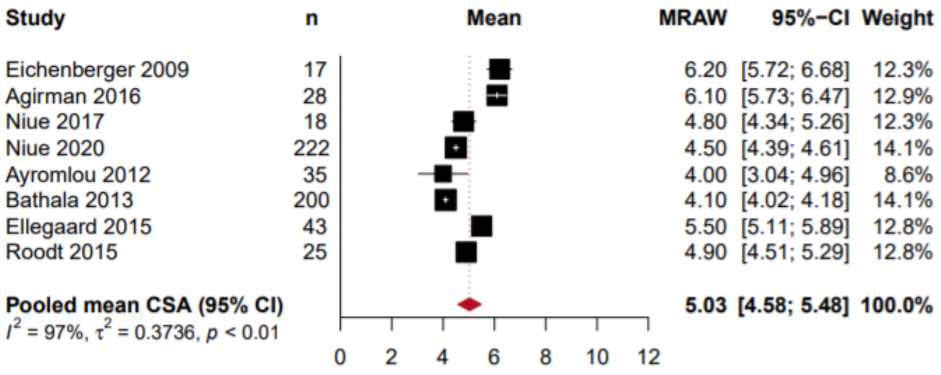

E. Cubital tunnel outlet

Study	n	MRAW	95%−CI	Weight
Cartwright 2007	60	6.40	[6.12; 6.68]	5.9%
Elias 2009	20	5.19	[4.62; 5.76]	5.8%
Kutlay 2009	40	5.70	[5.30; 6.10]	5.9%
Pompe 2013	73	5.10	[4.73; 5.47]	5.9%
Pazzaglia 2013	37	6.50	[6.11; 6.89]	5.9%
Won 2013	194	7.40	[7.20; 7.60]	5.9%
Yalcin 2013	144	5.60	[5.44; 5.76]	6.0%
Jang 2014	36	8.60	[8.21; 8.99]	5.9%
Yalcin 2014	38	5.60	[5.25; 5.95]	5.9%
Ellegaard 2015	43	5.90	[5.45; 6.35]	5.8%
Ghanei 2015	44	7.80	[7.39; 8.21]	5.9%
Gupta 2016	60	4.00	[3.97; 4.03]	6.0%
Kang 2016	20	7.37	[7.01; 7.73]	5.9%
Chen 2017	200	6.26	[6.08; 6.44]	6.0%
Niue 2017	18	5.10	[4.45; 5.75]	5.7%
Rayegani 2019	34	7.57	[7.12; 8.02]	5.8%
Niue 2020	222	4.90	[4.78; 5.02]	6.0%
**Pooled mean CSA (95% CI)**		**6.18**	**[5.45; 6.90]**	**100.0%**

$I^2 = 100\%$, $\tau^2 = 2.2860$, $p = 0$

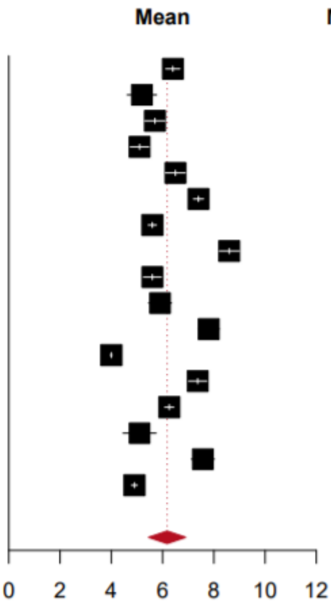

F. Tip of the medial epicondyle

Study	n	MRAW	95%−CI	Weight
Jacob 2004	400	7.90	[7.60; 8.20]	2.8%
Cartwright 2007	60	6.50	[6.27; 6.73]	2.8%
Thoirs 2008	107	8.20	[7.59; 8.81]	2.7%
Yoon 2008 (2)	30	6.60	[6.24; 6.96]	2.8%
Jain 2009	60	8.50	[7.61; 9.39]	2.6%
Kutlay 2009	40	5.60	[5.10; 6.10]	2.8%
Hooper 2011	64	7.61	[7.42; 7.80]	2.8%
Ayromlou 2012	35	4.00	[3.04; 4.96]	2.6%
Bathala 2012	200	4.70	[4.62; 4.78]	2.9%
Boom & Visser 2012	37	6.17	[5.67; 6.67]	2.8%
Childs 2012 (European)	48	7.00	[6.43; 7.57]	2.8%
Childs 2012 (Asian)	24	7.00	[6.20; 7.80]	2.7%
Cartwright 2013	36	10.60	[9.62; 11.58]	2.6%
Kerasnoudis 2013	150	5.33	[5.11; 5.55]	2.8%
Pompe 2013	73	5.30	[4.93; 5.67]	2.8%
Sugimoto 2013	120	6.70	[6.36; 7.04]	2.8%
Yalcin 2013	144	6.20	[6.02; 6.38]	2.9%
Boehm 2014	56	7.20	[6.65; 7.75]	2.8%
Jang 2014	36	8.70	[8.37; 9.03]	2.8%
Scheidl 2014	34	7.60	[6.89; 8.31]	2.7%
Yalcin 2014	38	5.70	[5.35; 6.05]	2.8%
Ellegaard 2015	43	6.80	[6.35; 7.25]	2.8%
Roodt 2015	25	5.20	[4.81; 5.59]	2.8%
Schreiber 2015	36	7.60	[6.82; 8.38]	2.7%
Afsal 2016	20	5.30	[4.60; 6.00]	2.7%
Agirman 2016	28	7.70	[7.29; 8.11]	2.8%
Merola 2016	140	5.94	[5.64; 6.24]	2.8%
Qrimli 2016	185	6.90	[6.57; 7.23]	2.8%
Yagci 2016	60	8.21	[7.90; 8.52]	2.8%
Chen 2017	200	5.87	[5.66; 6.08]	2.8%
Grimm 2018	100	8.70	[8.31; 9.09]	2.8%
Lothet 2019	30	9.27	[8.46; 10.08]	2.7%
Rayegani 2019	34	8.27	[7.69; 8.85]	2.8%
Singh 2019	45	6.50	[6.09; 6.91]	2.8%
Tahmaz 2020	160	7.10	[6.82; 7.38]	2.8%
Tandon 2020	60	3.11	[2.91; 3.31]	2.8%
**Pooled mean CSA (95% CI)**		**6.81**	**[6.31; 7.31]**	**100.0%**

$I^2 = 99\%$, $\tau^2 = 2.2579$, $p = 0$

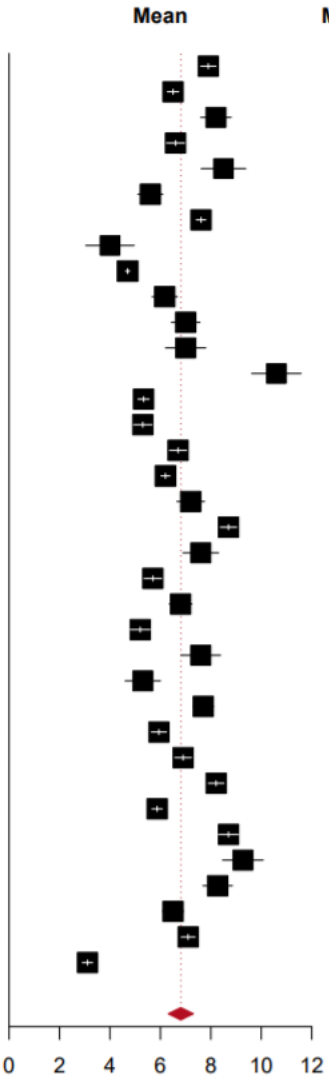

G. Cubital tunnel

Study	n	MRAW	95%-CI	Weight
Wiesler 2006	60	6.50	[6.25; 6.75]	6.9%
Ozturk 2008	212	6.60	[6.37; 6.83]	6.9%
Tagliafico 2008 (1)	50	6.80	[6.47; 7.13]	6.8%
Elias 2009	20	6.84	[6.00; 7.68]	6.3%
Ayromlou 2012	35	5.00	[4.04; 5.96]	6.1%
Frade 2013	92	6.70	[6.25; 7.15]	6.7%
Pompe 2013	73	5.90	[5.49; 6.31]	6.8%
Scheidl 2013	87	7.60	[7.24; 7.96]	6.8%
Tagliafico 2013	80	5.90	[5.24; 6.56]	6.5%
Ghanei 2015	44	7.20	[6.70; 7.70]	6.7%
Cheng 2016	100	6.70	[6.45; 6.95]	6.9%
Gupta 2016	60	4.55	[4.54; 4.56]	6.9%
Niue 2017	18	6.20	[5.32; 7.08]	6.2%
Chen 2018	58	9.40	[8.71; 10.09]	6.5%
Niue 2020	222	5.60	[5.46; 5.74]	6.9%
**Pooled mean CSA (95% CI)**		**6.50**	**[5.80; 7.20]**	**100.0%**

$I^2 = 99\%$, $\tau^2 = 1.8282$, $p = 0$

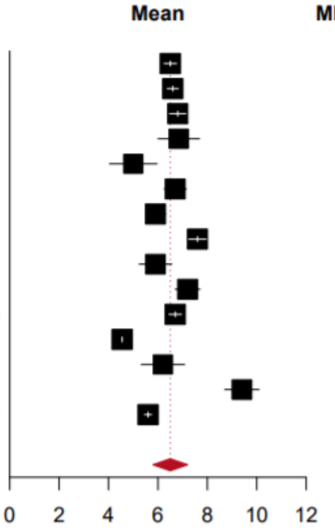

H. Cubital tunnel inlet

Study	n	MRAW	95%-CI	Weight
Cartwright 2007	60	6.70	[6.42; 6.98]	5.7%
Kutlay 2009	40	5.70	[5.20; 6.20]	5.5%
Visser 2012	25	5.32	[4.75; 5.89]	5.4%
Won 2013	194	7.20	[7.02; 7.38]	5.7%
Yalcin 2013	144	6.20	[6.02; 6.38]	5.7%
Jang 2014	36	7.70	[7.28; 8.12]	5.5%
Yalcin 2014	38	5.70	[5.35; 6.05]	5.6%
Ellegaard 2015	43	6.50	[6.17; 6.83]	5.6%
Ghanei 2015	44	7.80	[7.39; 8.21]	5.6%
Kim 2015	30	7.50	[7.03; 7.97]	5.5%
Kang 2016	20	7.29	[6.84; 7.74]	5.5%
Bedewi 2017	100	6.54	[6.21; 6.87]	5.6%
Chen 2017	200	6.38	[6.19; 6.57]	5.7%
Fink 2017	73	10.00	[9.43; 10.57]	5.4%
Niue 2017	18	4.70	[4.15; 5.25]	5.4%
Chen 2018	58	9.10	[8.53; 9.67]	5.4%
Rayegani 2019	34	7.52	[7.00; 8.04]	5.5%
Niue 2020	222	4.90	[4.78; 5.02]	5.7%
**Pooled mean CSA (95% CI)**		**6.81**	**[6.26; 7.36]**	**100.0%**

$I^2 = 98\%$, $\tau^2 = 1.3710$, $p < 0.01$

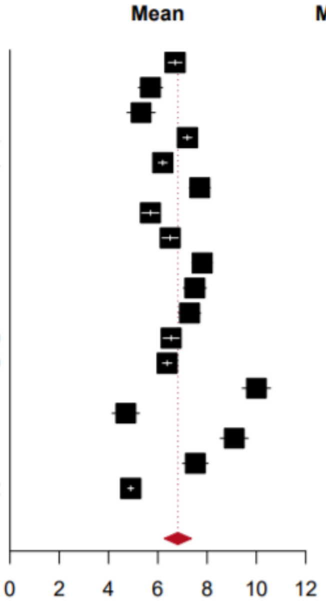

I. Distal upper arm

Study	n	MRAW	95%-CI	Weight
Ayromlou 2012	35	4.00	[3.50; 4.50]	9.5%
Bathala 2012	44	4.83	[4.53; 5.13]	12.5%
Bathala 2013	200	4.40	[4.32; 4.48]	14.9%
Ellegaard 2015	43	5.40	[5.07; 5.73]	12.0%
Roodt 2015	50	5.10	[4.85; 5.35]	13.2%
Gupta 2016	60	4.08	[4.04; 4.12]	15.1%
Niue 2017	18	4.40	[3.80; 5.00]	8.1%
Niue 2020	220	4.40	[4.29; 4.51]	14.7%
**Pooled mean CSA (95% CI)**		**4.58**	**[4.33; 4.83]**	**100.0%**

$I^2 = 96\%$, $\tau^2 = 0.1094$, $p < 0.01$

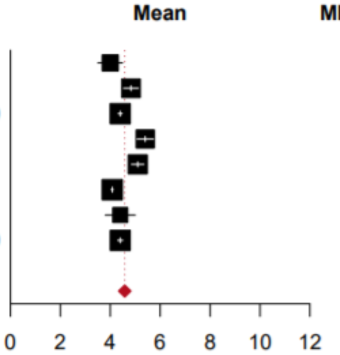

J. Mid-upper arm

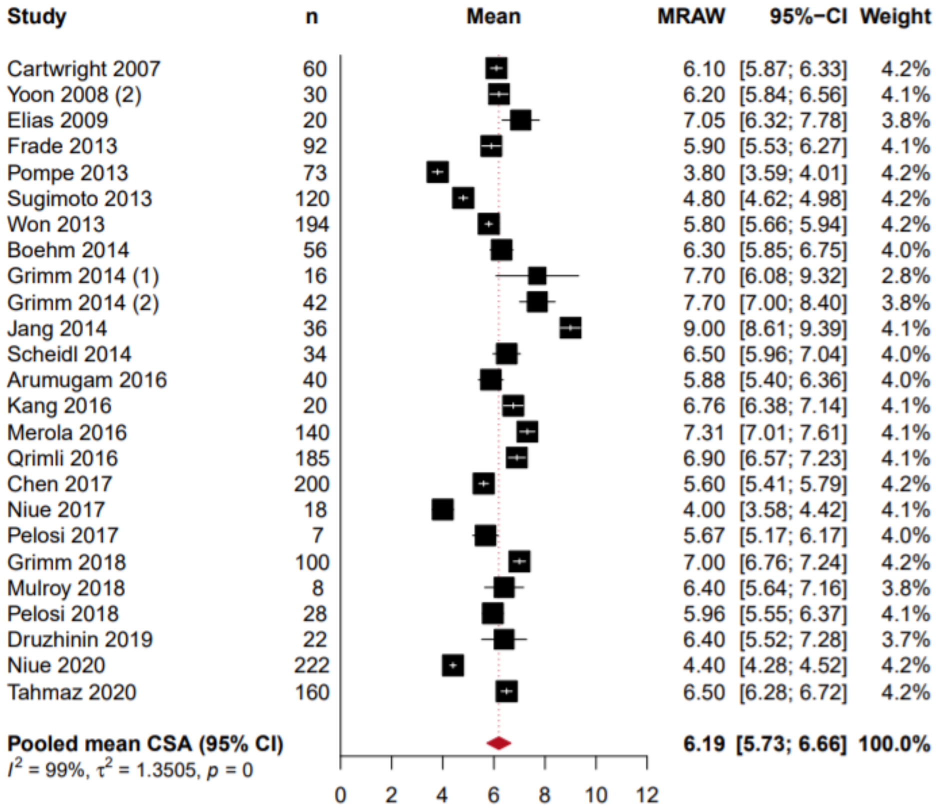

K. Axilla

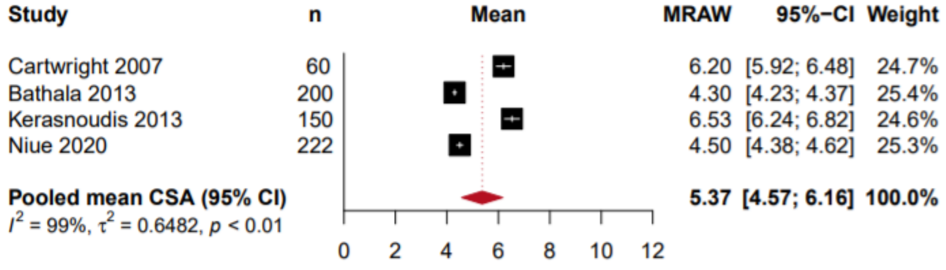

A: Includes measurements at the distal wrist crease and Guyon's canal; B: Includes measurements taken 2 cm proximal to the distal crease to the distal 1/3th of the forearm; C: Includes measurements taken at the mid-forearm, where the ulnar artery and nerve made contact; D: Includes measurements taken 2 cm proximal to the contact point of ulnar artery and nerve up to 3 cm distal to tip of the medial epicondyle; E: Includes measurements taken at the cubital tunnel outlet , 1-2 cm distal to medial epicondyle, between the two heads of the FCU muscle; F: Includes maximal CSA measurements between cubital tunnel inlet and outlet; G: Includes measurements taken at the tip of the medial epicondyle; H: Includes measurements at the cubital tunnel inlet, 1-2 cm proximal to medial epicondyle; I: Includes measurements 4-5 cm proximal to tip of the medial epicondyle; J. Includes measurements at the mid-upper arm.

Fig. 3 Forest plots of subgroup analyses, stratified for anatomical level

A. Wrist crease

<15 MHz versus ≥15 MHz

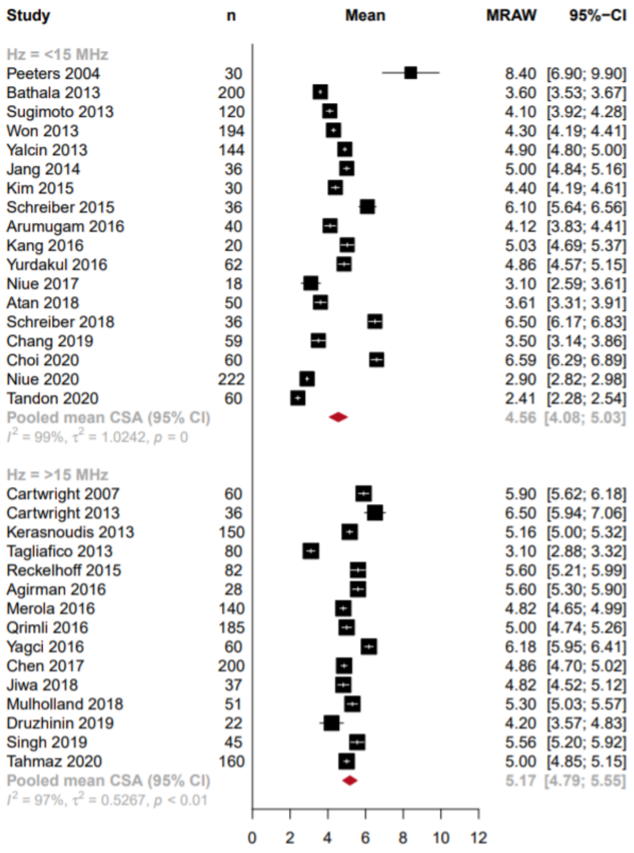

Diabetics: specifically excluded versus unknown or not excluded

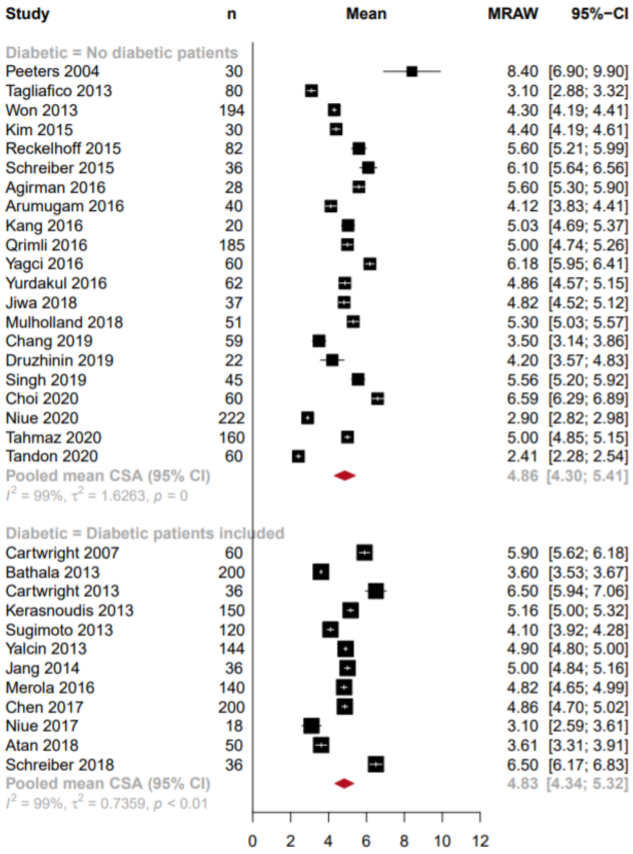

Position: flexion versus extension versus unknown

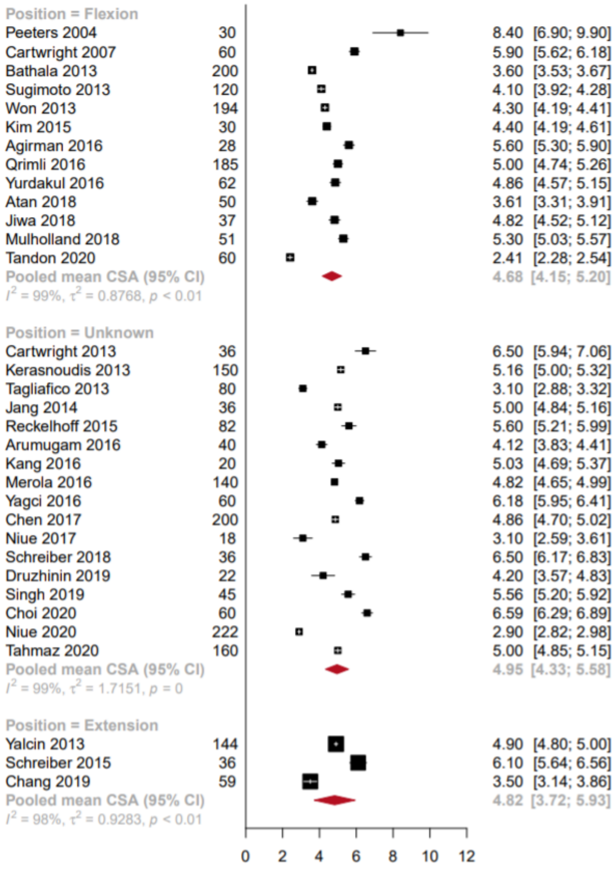

Ethnicity: Asian population versus other population

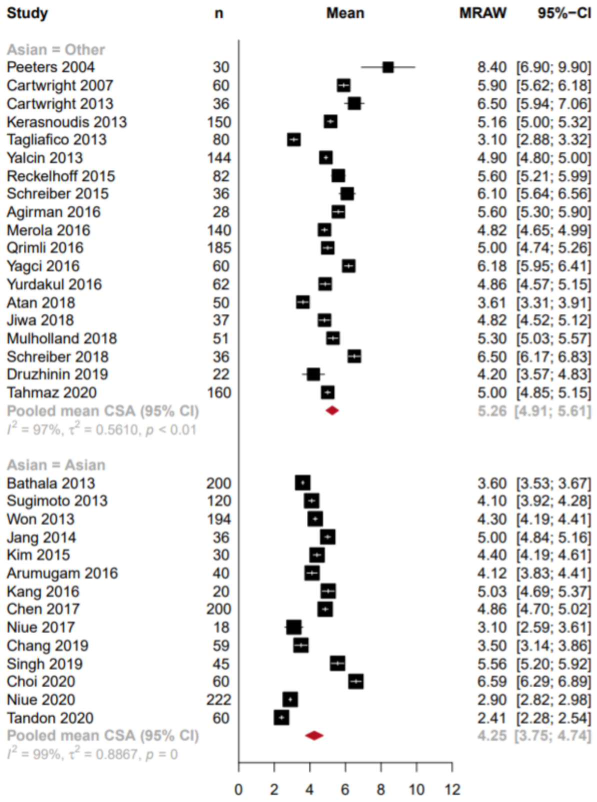

Gender: female versus male

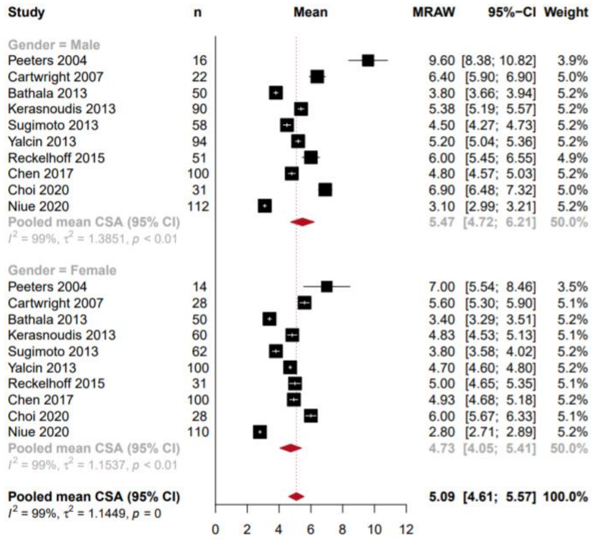

B. Proximal of wrist crease

<15 MHz versus ≥15 MHz

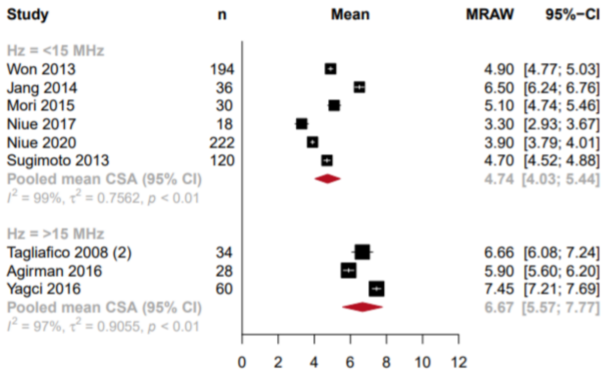

Diabetics: specifically excluded versus unknown or not excluded

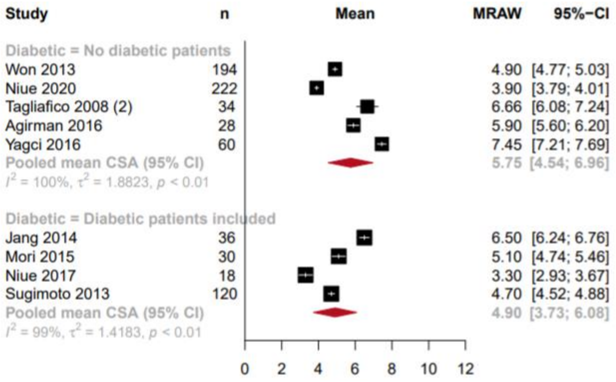

Position: flexion versus extension versus unknown

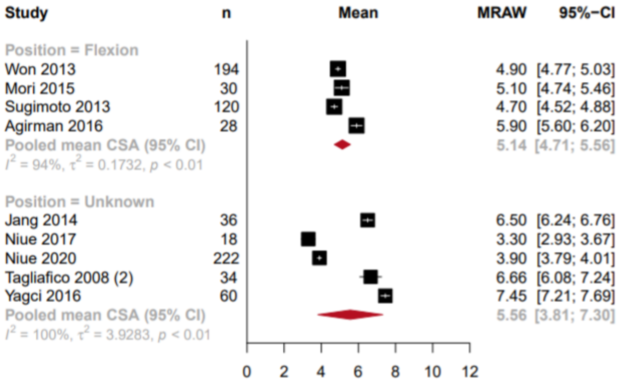

Ethnicity: Asian population versus other population

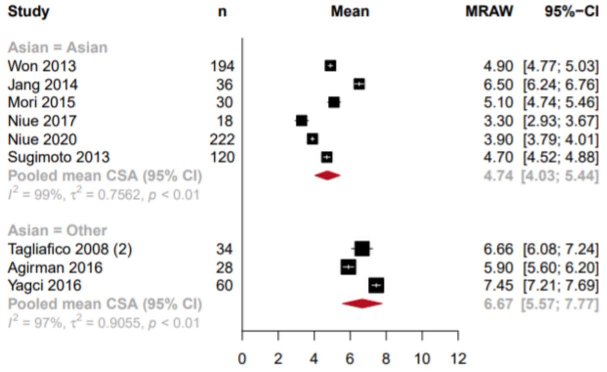

C. Mid-forearm

<15 MHz versus ≥15 MHz

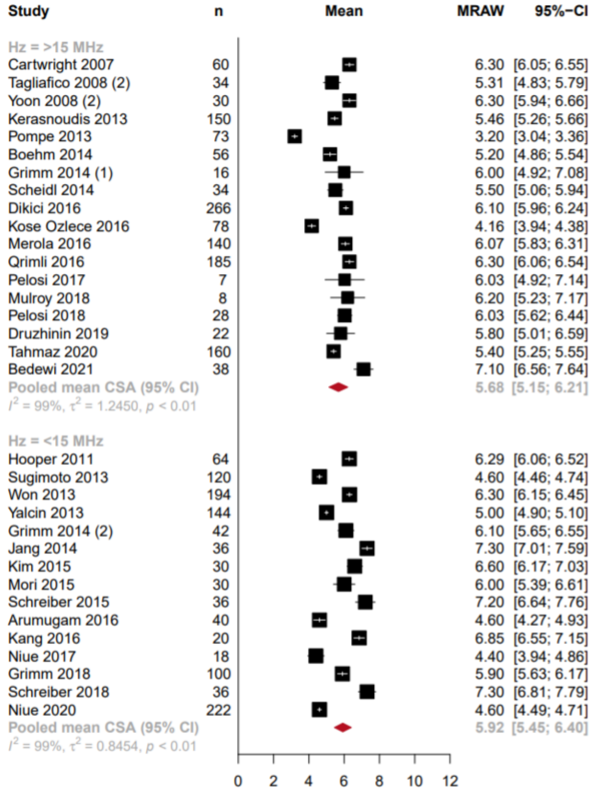

Diabetics: specifically excluded versus unknown or not excluded

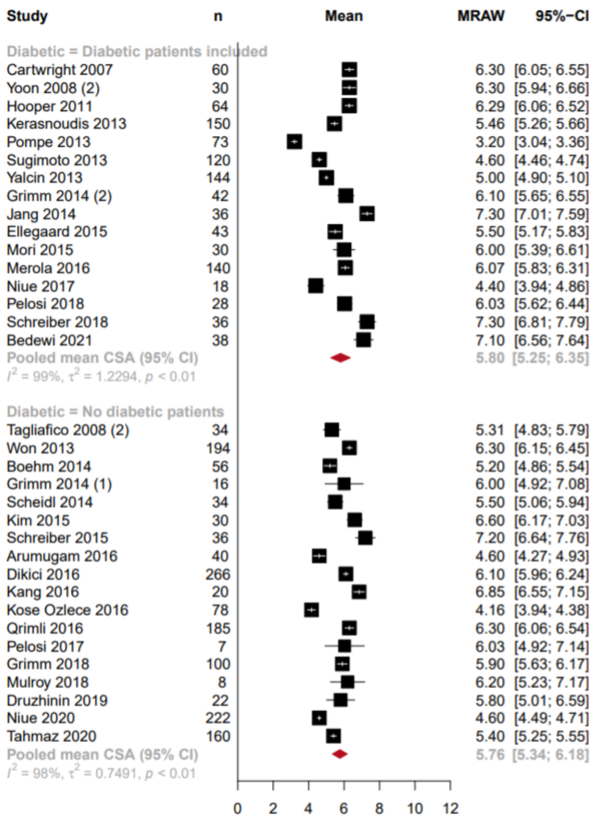

Position: flexion versus extension versus unknown

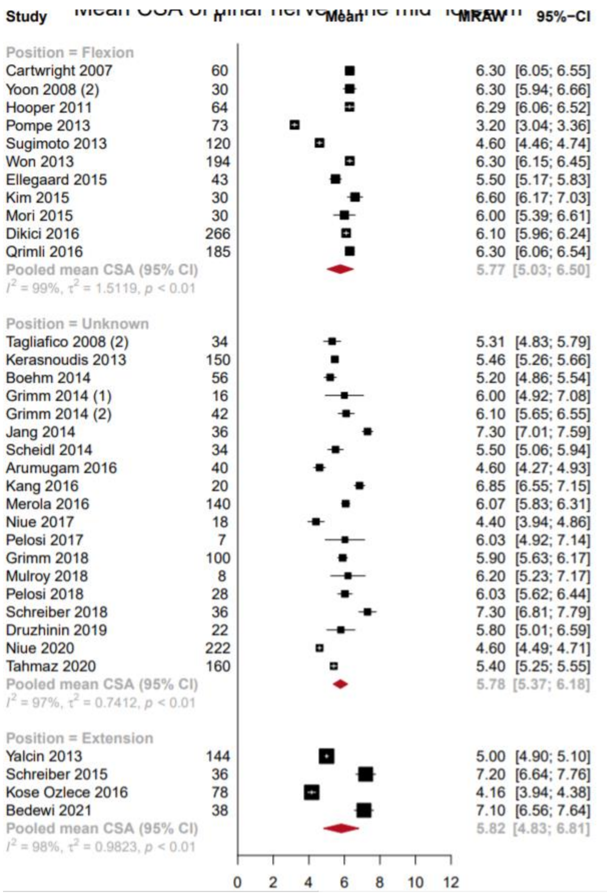

Ethnicity: Asian population versus other population

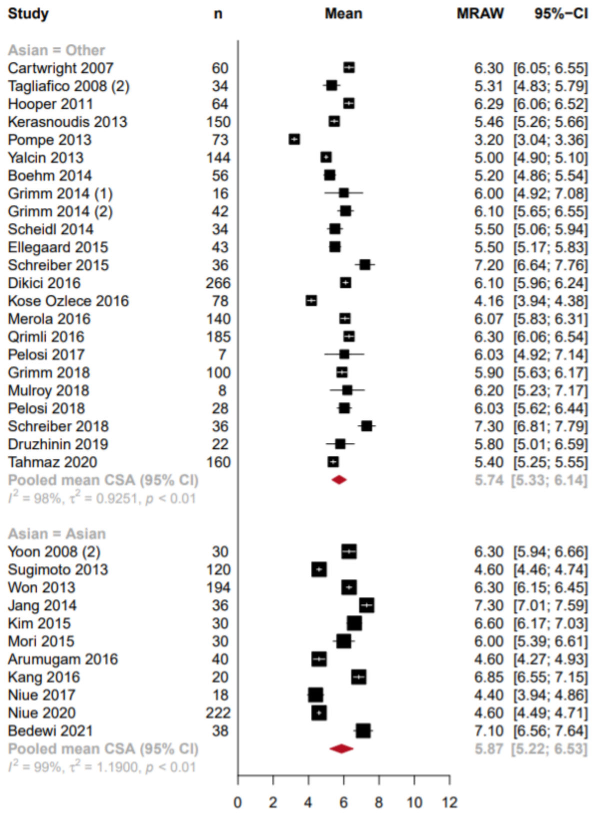

Gender: female versus male

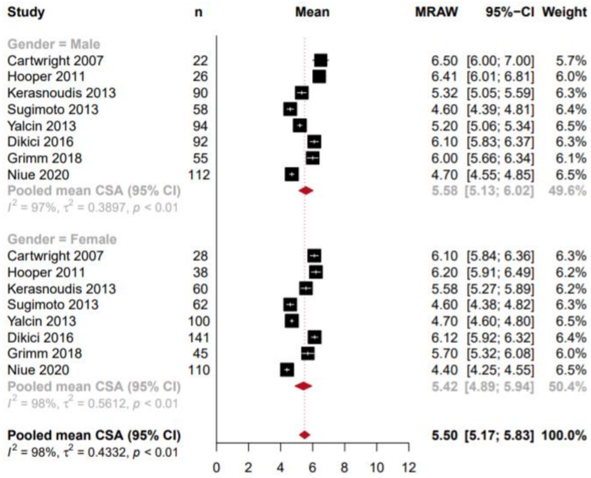

D. Proximal forearm

Diabetics: specifically excluded versus unknown or not excluded

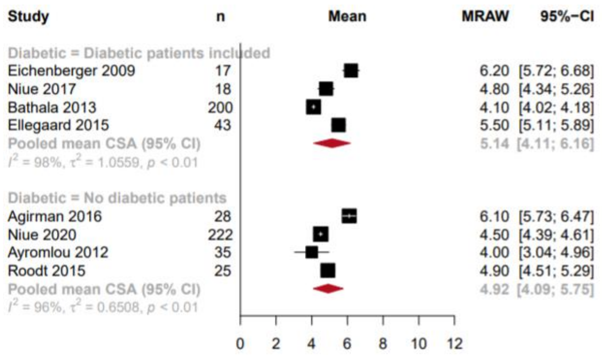

Ethnicity: Asian population versus other population

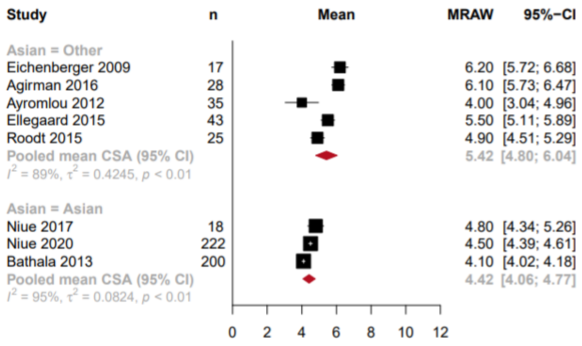

E. Cubital tunnel outlet

<15 MHz versus ≥15 MHz

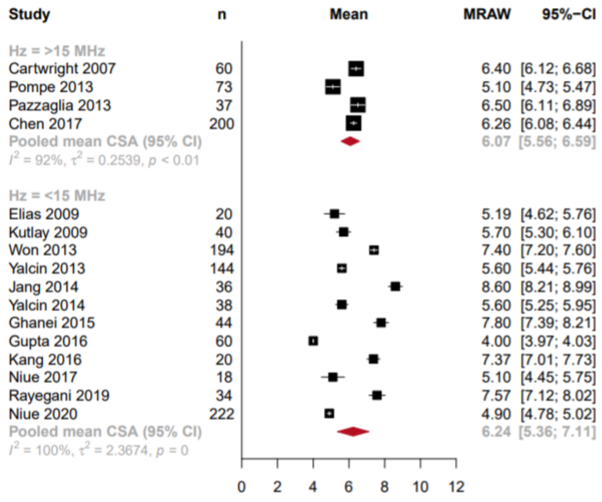

Diabetics: specifically excluded versus unknown or not excluded

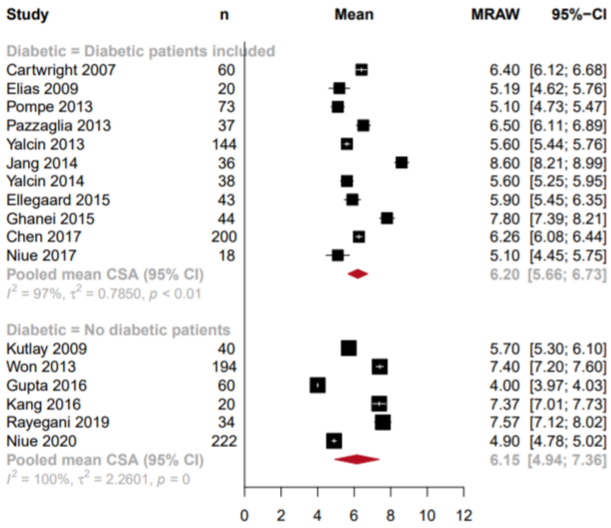

Position: flexion versus extension versus unknown

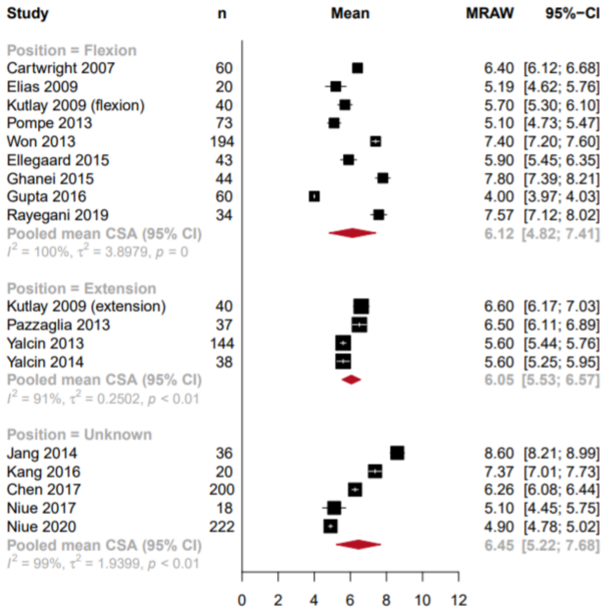

Ethnicity: Asian population versus other population

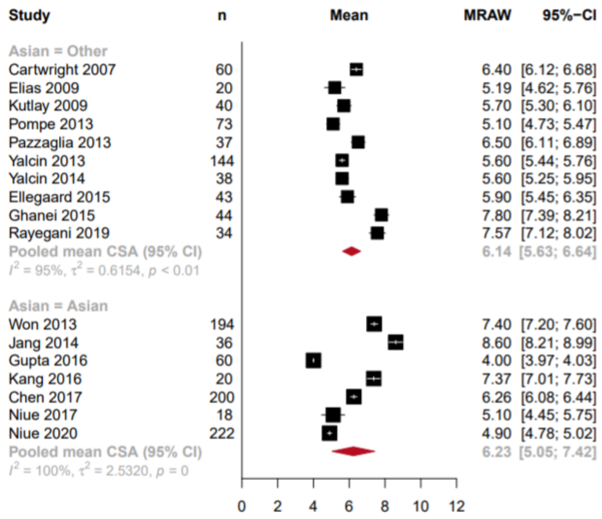

Gender: female versus male

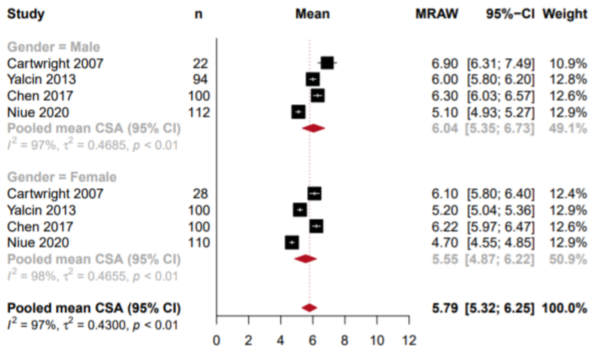

F. Cubital tunnel

<15 MHz versus ≥15 MHz

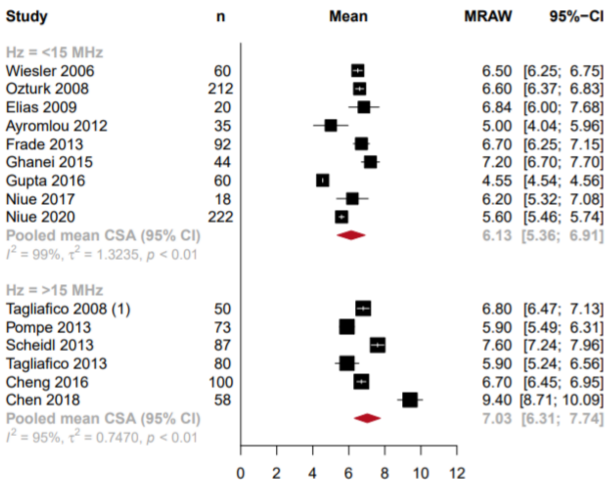

Diabetics: specifically excluded versus unknown or not excluded

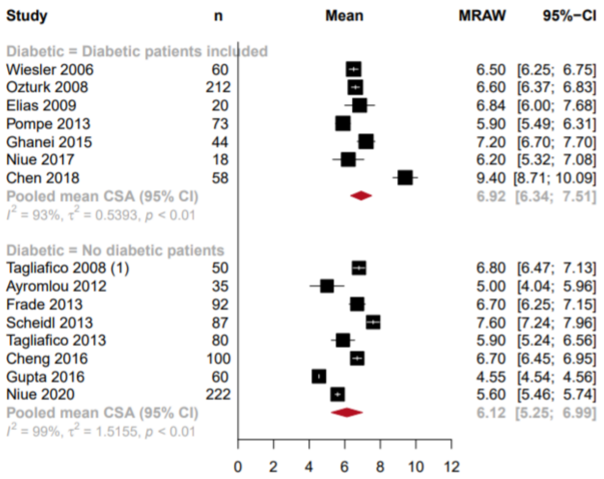

Position: flexion versus extension versus unknown

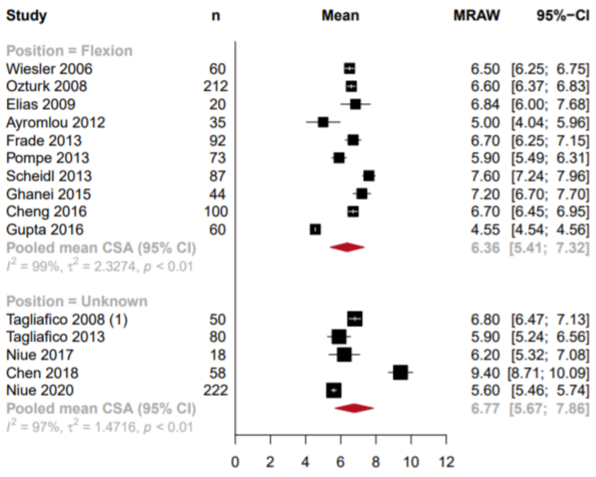

Ethnicity: Asian population versus other population

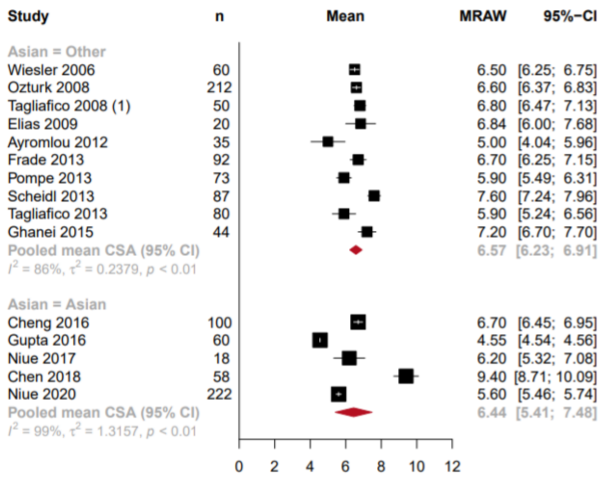

Gender: female versus male

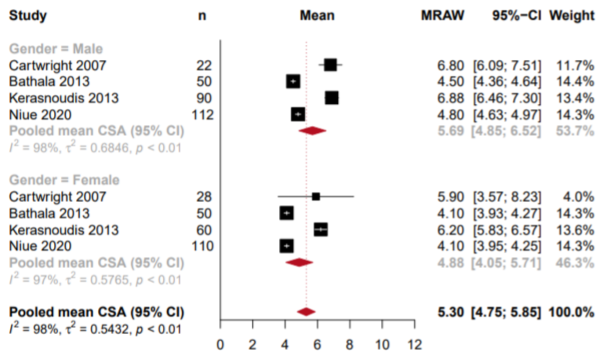

G. Medial epicondyle

<15 MHz versus ≥15 MHz

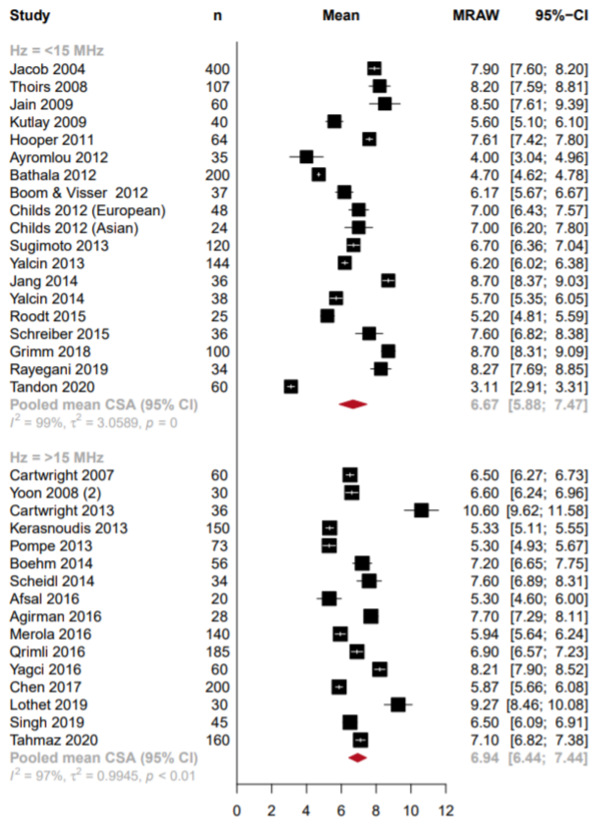

Diabetics: specifically excluded versus unknown or not excluded

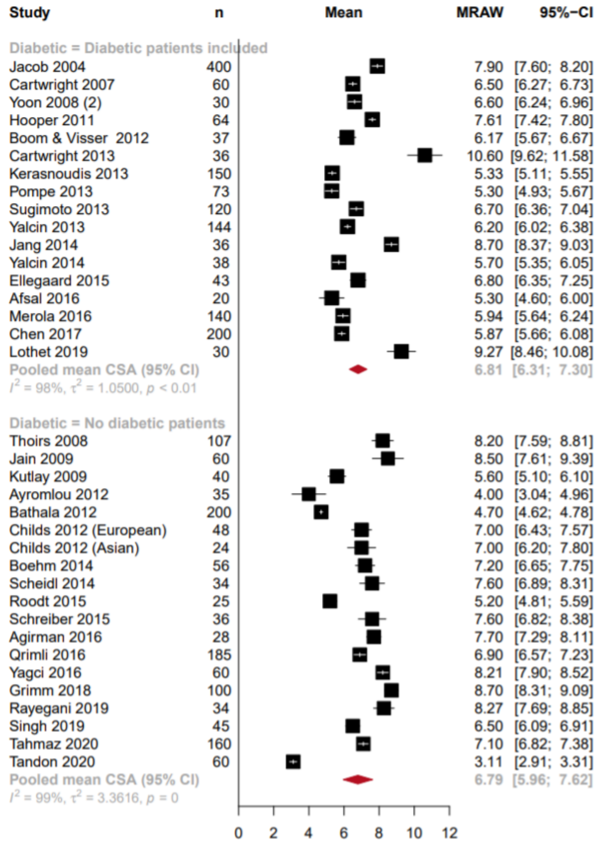

Position: flexion versus extension versus unknown

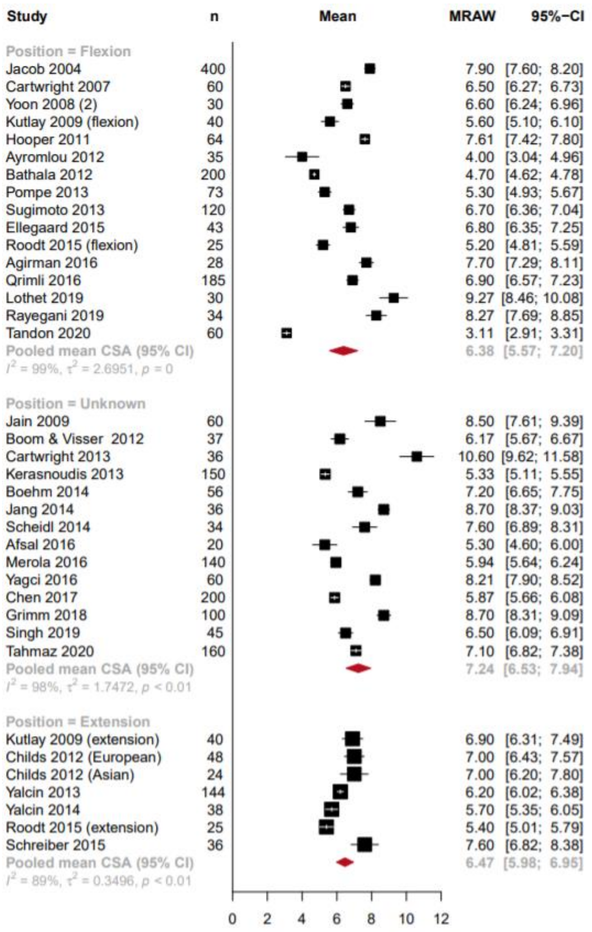

Ethnicity: Asian population versus other population

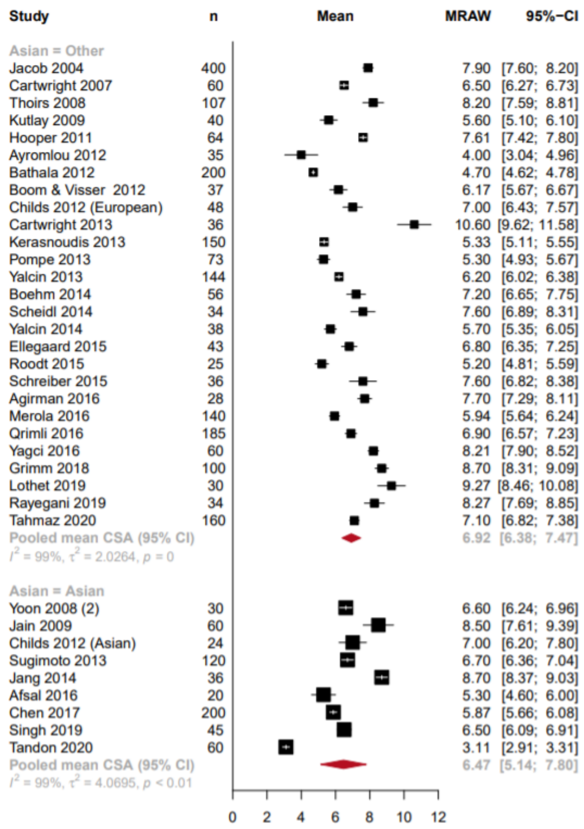

Gender: female versus male

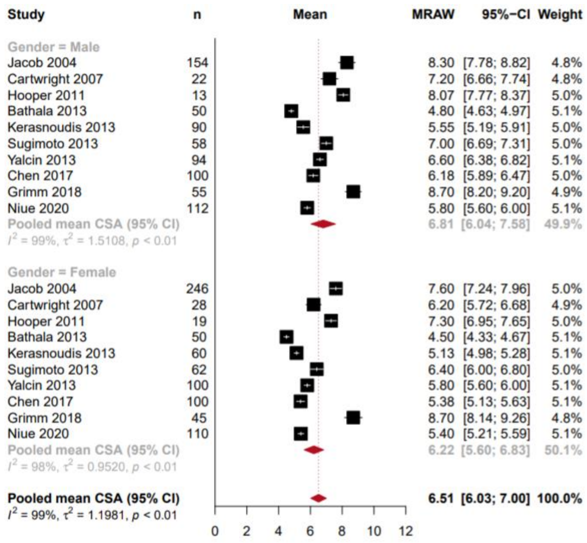

H. Cubital tunnel inlet

<15 MHz versus ≥15 MHz

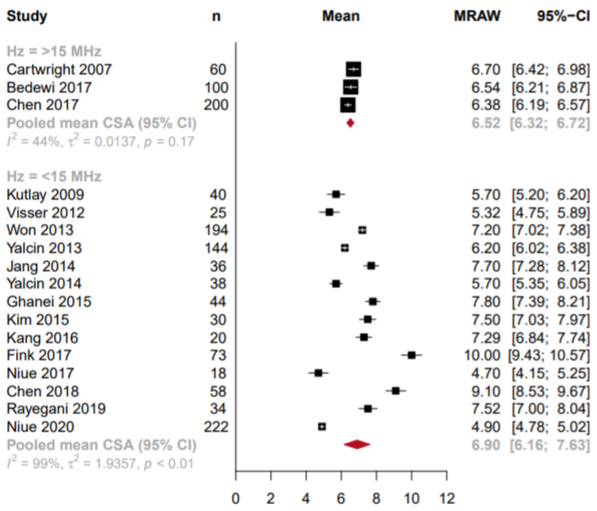

Diabetics: specifically excluded versus unknown or not excluded

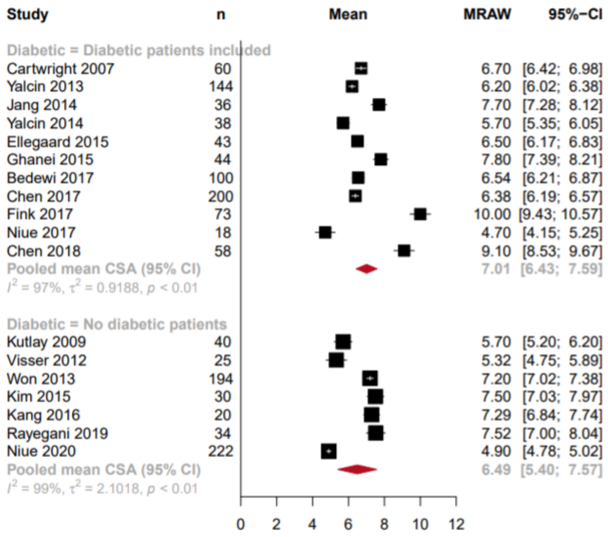

Position: flexion versus extension versus unknown

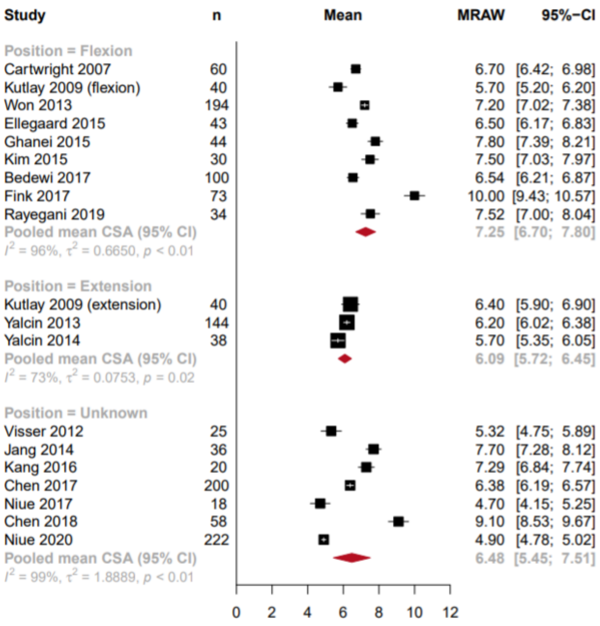

Ethnicity: Asian population versus other population

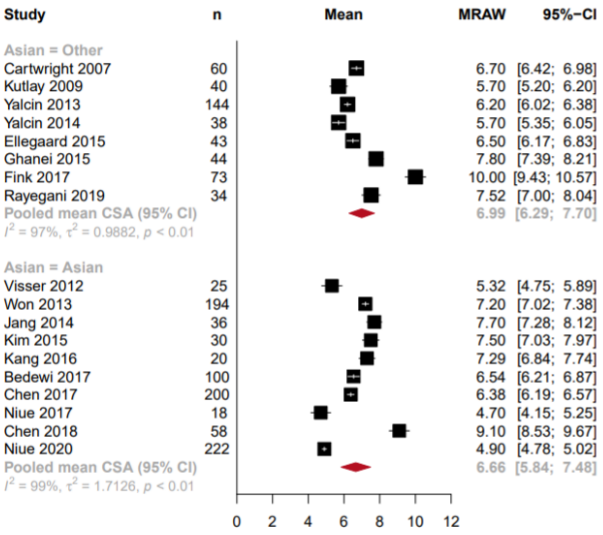

Gender: female versus male

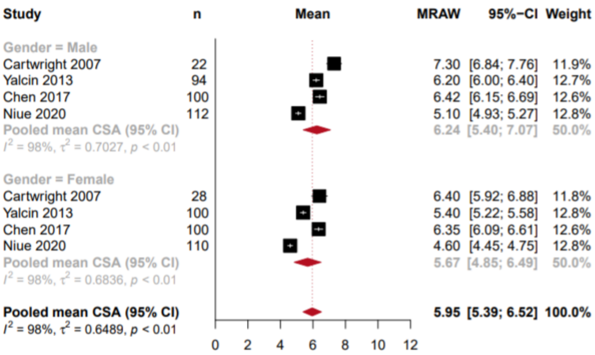

I. Distal upper arm

Diabetics: specifically excluded versus unknown or not excluded

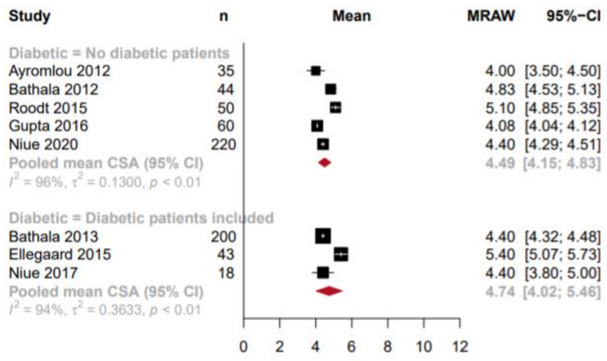

Ethnicity: Asian population versus other population

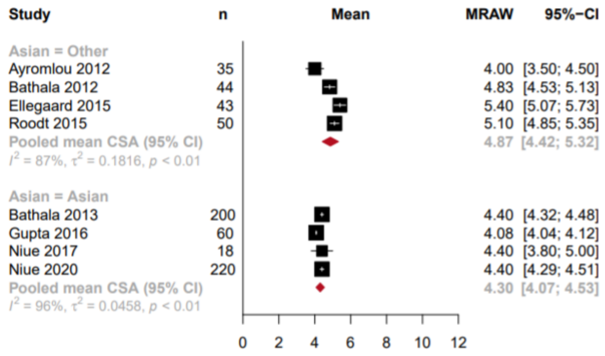

J. Mid-upper arm

<15 MHz versus ≥15 MHz

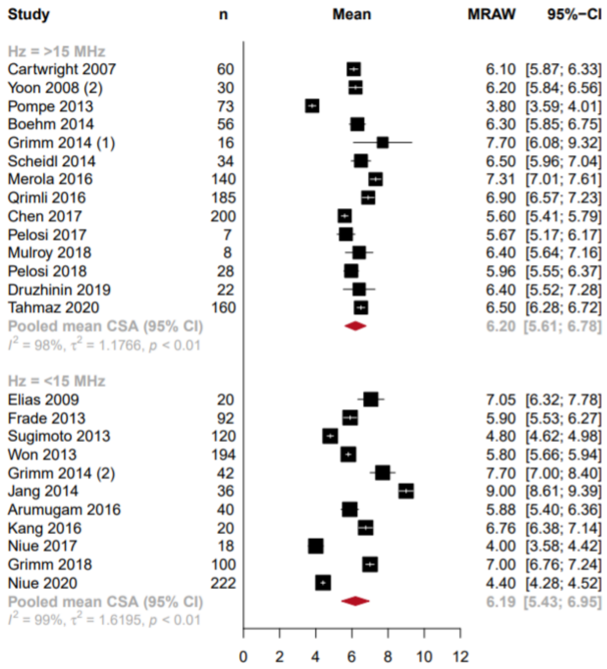

Diabetics: specifically excluded versus unknown or not excluded

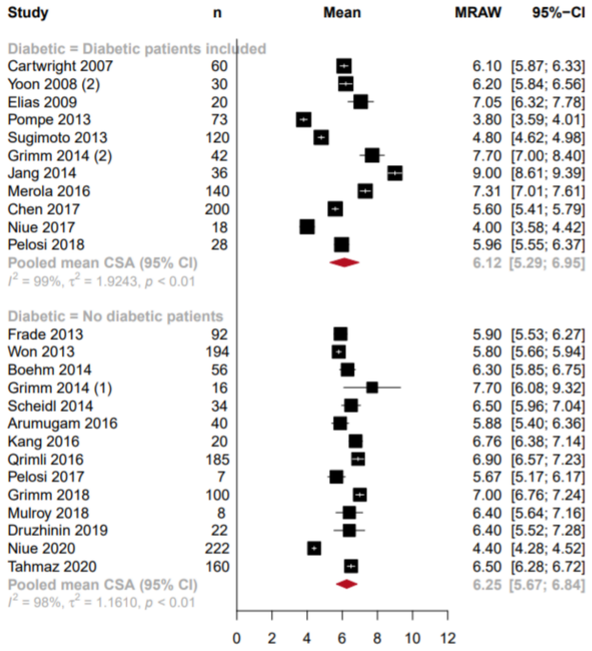

Position: flexion versus extension versus unknown

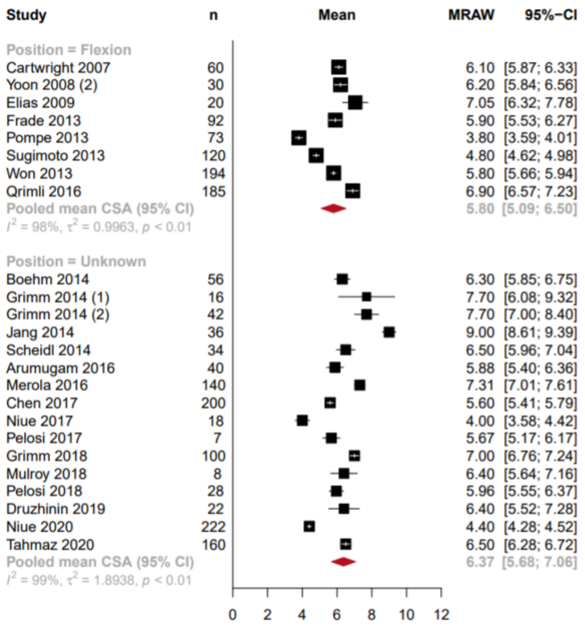

Ethnicity: Asian population versus other population

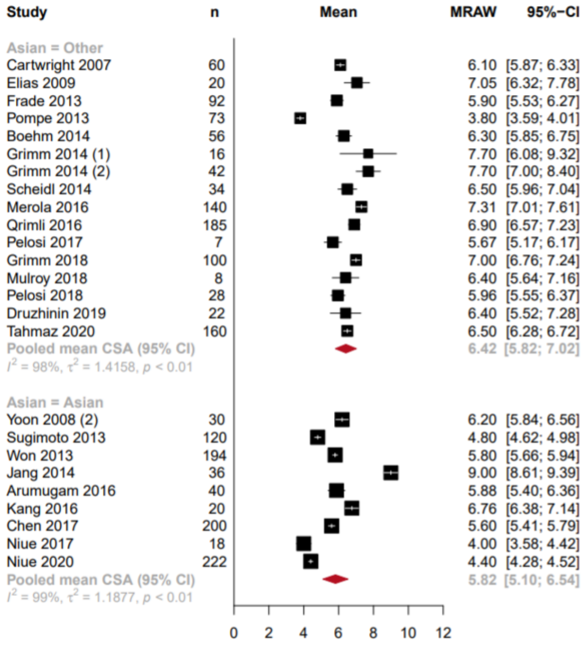

Gender: female versus male

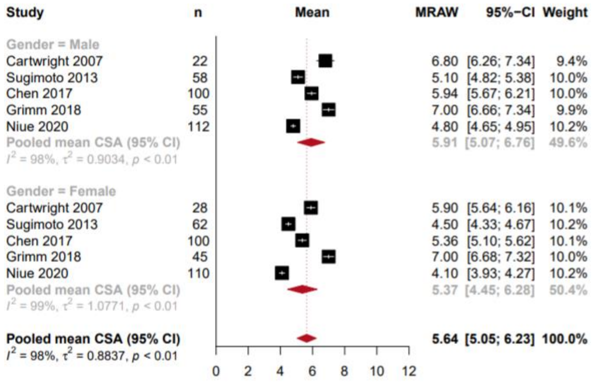

K. Axilla

Gender: female versus male

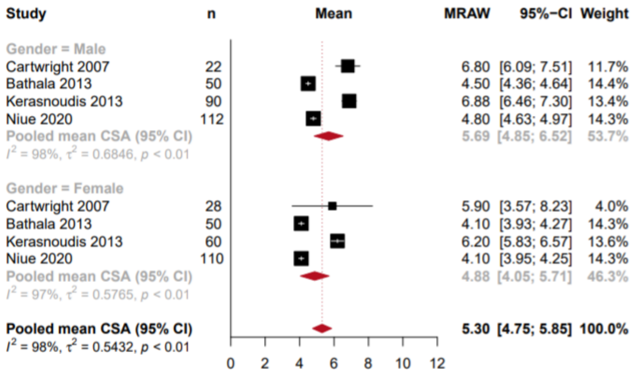

A: Includes measurements at the distal wrist crease and Guyon’s canal; B: Includes measurements taken 2 cm proximal to the distal crease to the distal 1/3th of the forearm; C: Includes measurements taken at the mid-forearm, where the ulnar artery and nerve made contact; D: Includes measurements taken 2 cm proximal to the contact point of ulnar artery and nerve up to 3 cm distal to tip of the medial epicondyle; E: Includes measurements taken at the cubital tunnel outlet , 1-2 cm distal to medial epicondyle, between the two heads of the FCU muscle; F: Includes

maximal CSA measurements between cubital tunnel inlet and outlet; G: Includes measurements taken at the tip of the medial epicondyle; H: Includes measurements at the cubital tunnel inlet, 1-2 cm proximal to medial epicondyle; I: Includes measurements 4-5 cm proximal to tip of the medial epicondyle; J. Includes measurements at the mid-upper arm.

95% CI = 95% confidence interval of the mean CSA value; CSA = cross-sectional area; MRAW = raw measurement; n = number
